# Comprehensive molecular interaction map of TGFβ induced epithelial to mesenchymal transition in breast cancer

**DOI:** 10.1038/s41540-024-00378-w

**Published:** 2024-05-17

**Authors:** Sai Bhavani Gottumukkala, Trivadi Sundaram Ganesan, Anbumathi Palanisamy

**Affiliations:** 1 Department of Biotechnology, National Institute of Technology Warangal, Warangal, India; 2 Department of Medical Oncology, Sri Ramachandra Institute of Higher Education and Research, Chennai, India

**Keywords:** Regulatory networks, Cancer

## Abstract

Breast cancer is one of the prevailing cancers globally, with a high mortality rate. Metastatic breast cancer (MBC) is an advanced stage of cancer, characterised by a highly nonlinear, heterogeneous process involving numerous singling pathways and regulatory interactions. Epithelial–mesenchymal transition (EMT) emerges as a key mechanism exploited by cancer cells. Transforming Growth Factor-β (TGFβ)-dependent signalling is attributed to promote EMT in advanced stages of breast cancer. A comprehensive regulatory map of TGFβ induced EMT was developed through an extensive literature survey. The network assembled comprises of 312 distinct species (proteins, genes, RNAs, complexes), and 426 reactions (state transitions, nuclear translocations, complex associations, and dissociations). The map was developed by following Systems Biology Graphical Notation (SBGN) using Cell Designer and made publicly available using MINERVA (http://35.174.227.105:8080/minerva/?id=Metastatic_Breast_Cancer_1). While the complete molecular mechanism of MBC is still not known, the map captures the elaborate signalling interplay of TGFβ induced EMT-promoting MBC. Subsequently, the disease map assembled was translated into a Boolean model utilising CaSQ and analysed using Cell Collective. Simulations of these have captured the known experimental outcomes of TGFβ induced EMT in MBC. Hub regulators of the assembled map were identified, and their transcriptome-based analysis confirmed their role in cancer metastasis. Elaborate analysis of this map may help in gaining additional insights into the development and progression of metastatic breast cancer.

## Introduction

Metastatic breast cancer (MBC) is a lethal form of breast cancer with high incidence and mortality rates^1,2^. MBC is a heterogenous disease characterised by differences between and within tumours. It is primarily distinguished based on a variety of characteristic markers such as ER^+/−^, PR^+/−^, HER^+/−^, Ki-67. MBC arises as a multistep process with cancer cells spreading to various organs such as bone, brain, lung, and liver^1,3,4^. Epithelial to mesenchymal transition (EMT) is a critical development process that significantly contributes to the initiation and progression of metastasis depending upon the microenvironmental cues^5^. During EMT, non-invasive breast cancer transforms into invasive breast cancer by losing their polarised epithelial assets and acquiring invasive migratory properties of mesenchymal stem cells^6–8^. The progression of EMT is characterised by the status of tight junctions, cell polarity, expression of various epithelial (E-Cadherin, β-Catenin, p-120, few miRNAs) and mesenchymal markers (SNAIL, ZEB, TWIST, N-Cadherin) regulated through multiple signalling pathways.

Transforming growth factor β (TGFβ) and its family of cytokines are well known for their stage-dependent dual role as tumour suppressors in the early stages of cancer and as promotors of proliferation at a later stage^9–21^. The role of TGFβ is recognised in many cancer types, if not all^12,16,19,22–27^. It regulates various physiological and biological processes such as cell proliferation, apoptosis, differentiation, and migration^11,19,21,28–31^. Experimental studies have identified the significant role of TGFβ signalling in promoting EMT in breast cancer epithelial cells leading to MBC^14,32–37^. This regulation emerges because of the activation of its serine/threonine receptors through phosphorylation. This consequently activates a signalling cascade downstream including SMAD-dependent and SMAD-independent pathways which are involved in modulating the process of EMT in breast cancer cells^11,19,28,38–41^. Thus, the initiation and progression of TGFβ induced metastasis in breast cancer are regulated through multiple signalling molecules and mechanisms.

Genetic heterogeneity plays a pivotal role in driving metastasis and therapeutic relapse in cancer. This is influenced by the spatio-temporal expression of a multitude of genes in response to external stress^42^. This vast molecular heterogeneity and cellular characteristics exhibited by MBC across different patients present a significant challenge in identifying a reliable biomarker for EMT. The current study provides a comprehensive understanding of TGFβ induced EMT in MBC. This work emphasises cancer as a network-level disease that involves multiple signalling pathways and their intricate molecular interactions. Pathway databases like IMEx consortium, Signor, IntAct, OMNIpath Cancer Genome Atlas provide diagrammatic representations of the mechanisms implicated in complex signalling pathways associated with various diseases^43–45^. The ever-increasing need to understand the molecular players, mechanisms in complex diseases such as cancer underscores the growing need for disease maps^46^. The rise in the number of disease maps in the past two decades indicates the need for such maps which may guide the computational pipelines, develop experimental hypothesis and support drug-related studies. There are consortiums that focus on the development, quality, standards and periodical update of such maps. The present study specifically focuses on the functional interactions between downstream effectors of TGF-β signaling that contribute to the development of EMT-driven MBC. The molecular interaction mechanisms were explored in detail. The map developed may serve the community to develop hypotheses and may assist in exploring them experimentally or theoretically.

## Results

### Pathway map of TGFβ induced EMT in MBC

**Fig. 1:**
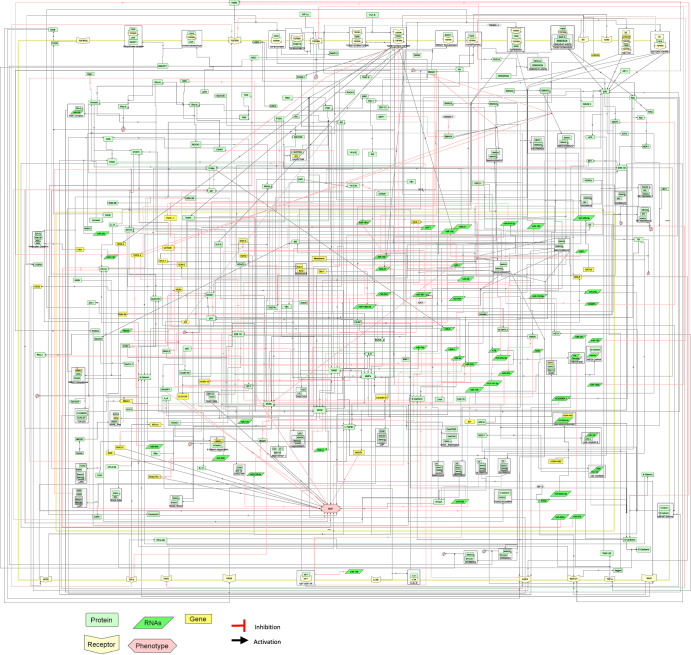
Molecular regulatory map of metastatic breast cancer. The map represents proteins in green, RNAs in lime green, genes in canary, and phenotypes in pink. Interactions among regulators are displayed in black and inhibitions are displayed in red. Compartments are distinguished as bounding boxes. The SBGN-compliant map consists of 312 species, 426 interactions built using Cell Designer V 4.4. The map captures the TGFβ induced epithelial-to-mesenchymal transition signalling network in metastatic breast cancer. TGFβ parallelly regulates other signaling pathways like TNF-α, integrins, EGFR, in modulating the regulators associated with mesenchymal phenotype and epithelial phenotype further moderating EM transition.

The comprehensive map developed for TGFβ induced EMT signalling in MBC is shown in Fig. 1. The map was assembled based on an extensive literature search (Fig. 10). The map captures the heterogeneous molecular processes and regulators implicated in TGFβ mediated signalling, particularly in the context of EMT and metastasis of breast cancer (Supplementary Table 1 and Supplementary Table 3). The MBC map comprises 312 distinct species interconnected through 426 reactions across two compartments (cytoplasm and nucleus). It also encompasses 160 proteins, 30 genes, 48 RNAs, 57 complexes, 11 degradations, and 5 unknowns altogether resulting in one end phenotype of epithelial–mesenchymal transition (EMT). A range of molecular processes including 309 state transitions (activation and inhibition), 48 complex associations, 2 dissociations, 10 transports, 22 unknown transitions, 29 logical activations were categorised among the species. The “unknown transitions” in the map indicate instances where regulatory interactions are acknowledged, but the precise underlying mechanisms require additional experimental exploration for a comprehensive understanding. “Unknown” regulators represent intermediates in these processes that warrant further investigation.

**Fig. 2:**
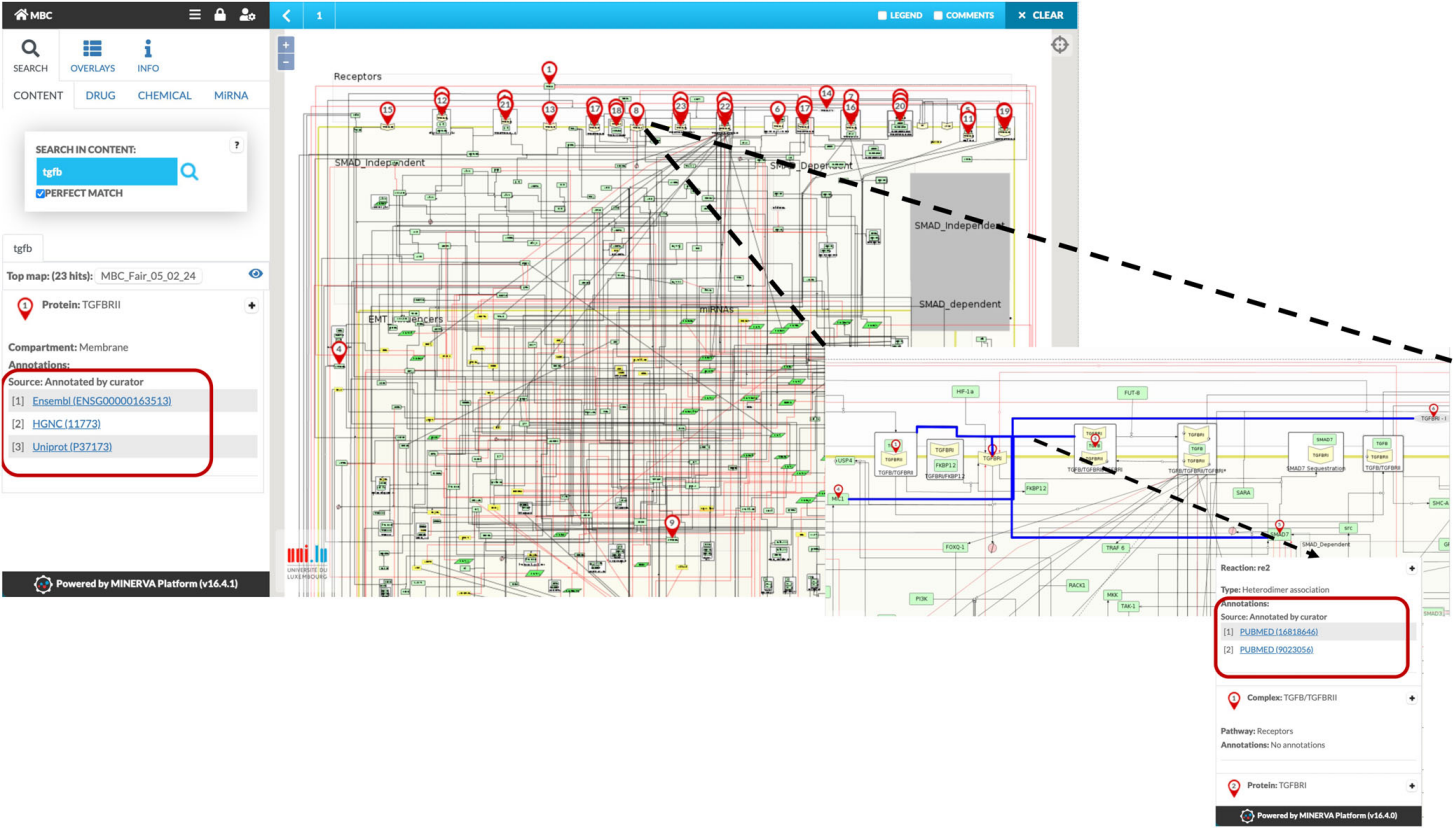
MBC Map in the MINERVA platform. Users can search for their regulator of interest from the search box. The results are shown as pins. The annotations of the corresponding regulators like HGNC ID, Uniport ID, Ensembl identifiers are displayed on the left upon selecting any element along with the PUBMED identifiers. Further users can also navigate interactions starting from a molecule of interest tracking the signal from TGFβ to the end phenotype. MAP is available at: http://35.174.227.105:8080/minerva/?id=Metastatic_Breast_Cancer_1.

The map was constructed using Cell Designer tool V 4.4 (http://celldesigner.org/). This map strictly adheres to the Systems Biology Graphics Notation (SBGN), ensuring an accurate representation of receptors, proteins, genes, RNAs, their modifications, as well as the complex associations and dissociations. The map portrays these entities as species, while their interactions in regulating the process of EMT are illustrated as reactions. Further, the storage of the map was implemented using Systems Biology Markup Language (SBML), an XML-based format widely used for communication^47^. All species names used in this study, their supporting literature and their corresponding HUGO names are shown (Supplementary Table 1). The map was further made available online using the MINERVA platform for active exploration, analysis and management (Fig. 2) and is available at: http://35.174.227.105:8080/minerva/?id=Metastatic_Breast_Cancer_1.

### Architecture of TGFβ induced EMT in MBC

In the developed map (Fig. 1), TGFβ stimulus acts as the input triggering the signalling cascade through activation of their serine/threonine kinase receptors. This subsequently regulates the SMAD-dependent as well as SMAD-independent signalling pathways such as RHO, RAS, P38, MAPK, STAT3, Wnt and EGFR. The map developed was further illustrated elaborately as zones (Supplementary Figs. 1–6). TGFβ induced SMAD-dependent and independent signalling pathways were observed to be integrating into the major regulators namely SNAIL, ZEB, E-Cadherin, N-Cadherin, β-Catenin, NF-kB, Twist, MMPs (Supplementary Figs. 7–10) in orchestrating the process of EMT and promoting metastasis of breast cancer. Supplementary Figs. 7–10 provide simplified illustrations around the major EMT regulators offering a clearer visual representation of the interactions and relationships between these key regulators. During the assembly process, efforts were made to maintain the closeness of components related to the SMAD-dependent and independent cascades. SMAD-independent pathway branches into multiple signalling pathways involving common regulators and interconnectedness between them. The regulators shared in both pathways makes the overall map highly nonlinear.

### SMAD-dependent signalling-based modulation of EMT in MBC

Mechanistically, TGFβ and its isoforms stimulate the SMAD signalling through the activation of its RTKs. TGFβ binds with its high-affinity TGFβRII receptor, causing a conformation change that creates a high-affinity binding site for TGFβRI^48^. This binding results in the activation of receptors through phosphorylation of TGFβRI in the juxta membrane subdomain by TGFβRII^49^. The activation of TGFβRI depends on the dissociation of FKBP12, a negative regulator of TGFβRI through the allosteric change in receptor confirmation^50,51^. Thus, the active form of the complex includes TGFβ ligand, and its phosphorylated type I and type II receptors. Upon activation, TGFβRI releases the SARA sequestered R-SMADs ensuing the phosphorylation of R-SMADs, particularly SMAD2 and SMAD3. The phosphorylated R-SMADs form a trimeric complex with Co-SMAD (SMAD4), which translocates into the nucleus modulating gene expression.

Subsequently, the translocated SMAD complex (R-SAMD/Co-SMAD) associates with co-factors to induce the effects of TGFβ stimulus either as tumour suppressor or as a promotor by regulating cell proliferation and/or migration. SMAD complex in association with FOXO, LAP (an inhibitory isoform of c/EBPb) activates the tumour suppressor role of TGFβ, while LIP is an isoform of c/EBPb which endorses the tumour promoting the role of TGFβ by inhibiting LAP^52^. Similarly, 14-3-3ζ was known to be overexpressed in malignant cells shifting the role of TGFβ from tumour suppressor to tumour activator through the activation of transcription factor Gli. Gli acts as a decisive partner of SMAD regulating PTHrP in fostering bone metastasis of breast cancer^53^. Elevated levels of PSPC1 in cancer cells result in the tumour-promoting role of TGFβ by its association with R-SMADs further promoting autocrine signalling through the activation of L-TGFβ^54^. Furthermore, GDF-10 activated by TGFβ/SMAD signalling, promotes the expression of SMAD7 and regulates the anti-proliferative effects suppressing EMT in TNBC cell lines^55^. SMAD complex associates with transcription factor AP-1 regulating the expression of genes like IL-11, CTGF, PTHrP, CXCR4 and thus promotes metastasis^33,56,57^. Mutant p53 as a cofactor of SMAD complex, regulates the role of SHARP, Cyclin G2 through p63 moderating metastasis^58^. ZO-1 an epithelial marker was known to be inhibited by TGFβ/SMAD signalling in promoting EMT^59^. MMPs regulated by TGFβ play a crucial role in maintaining the integrity of epithelial cells^60^. EMT was also observed to be altered by various negative feedback mechanisms regulated through TGFβ/SMAD-induced TMEPA1, SKI and Arkadia^37,42,61,62^. Thus, depending on the co-factors associated with SMAD-dependent signalling can support the anti-proliferative and proliferative role of TGFβ.

In addition to the direct interactions and feedback mechanisms discussed above, experimental evidence from TGFβ/SMAD signalling shows that the mutations in SMAD2 and SMAD4 are uncommon in breast cancer thus making SMAD3 a key player in the process of EMT^33,56^. However, the specific R-SMAD involved in the TGFβ/SMAD signalling is unknown. Thus, TGFβ/SMAD signalling modulates the process of EMT directly or indirectly in the presence of appropriate co-factors.

### SMAD-independent signalling-based Modulation of EMT in MBC

Numerous experimental studies have demonstrated the role of TGFβ-TGFβRII-TGFβRI complex in employing the SMAD-independent pathways in regulating EMT. These include the activation of PI3K/Akt^63–65^, STAT3^66,67^, c-Myc^52,68–70^, TNF-α/TRAF/TAK^36^, Annexin-2^66,67,71–75^-based signalling pathways. Phosphorylation of TGFβRII at Tyr residues by Src leads to the activation of Shc-A. This, in turn, facilitates the assembly of GRB2-SHC-A-SOS complex, which plays a crucial role in transducing downstream signalling through the p38/MAPK axis directly. Additionally, this pathway can also be influenced indirectly by β3 integrin signalling^76,77^. The functioning of TGFβ signalling in the participation of cytoskeletal rearrangement, cell polarity and migration by Rho GTPases can rapidly promote EMT in a SMAD2/3-independent manner^78^. Crosstalk between IGF1/PI3K signalling induced LTGFβ results in activation of TGFβ signalling promoting EMT in breast cancer^64^. This diverse upstream signalling modulated by TGFβ in SMAD-dependent and independent manner converges at regulators that are majorly associated with invasive properties of EMT in MBCs. These include SNAIL/SLUG, ZEB1/2, TWIST1/2, NF-kB, MMPs which are regulating the adherent junctions (E-Cadherin/β-Catenin) and further the process of EMT. This experimental evidence supports the critical role of TGFβ signalling in EMT.

SNAIL, a family of zinc finger transcription motifs representing SNAI1 (SNAIL), SNAI2 (SLUG) and SNAI3) are key regulators of TGFβ induced EMT in breast cancer cells^79–83^. TGFβ modulates the transcriptional activity of SNAIL through many paths (Supplementary Fig. 7). SNAIL/SLUG primarily functions as a repressor complex targeting the E-Boxes of E-Cadherin affecting its gene expression^79,84,85^. E-cadherin is a major epithelial marker that regulates the tight junctions when complexed with β-catenin. SNAIL promotes cancer cell invasion when it forms a complex with Sin3A-HDAC1/2 through SNAG domain repressing E-Cadherin. Conversely, SNAIL curbs invasion in the presence of p53, MDM2 in wild type^86–88^. The association of SNAIL with mutant p53 has been found to stabilise SNAIL, thereby promoting EMT^88,89^. However, when SNAIL is coupled with G9a, it facilitates the degradation of E-cadherin, leading to activation of invasive switch^90^. Additionally, SNAIL activity is amplified through the activation of NF-kB signalling regulated by TGFβ induced c-Myc, TRAF/TAK, TWIST, STAT3, ANXA2 and AKT^54,59,81,88,91–99^. SNAIL is also regulated through TGFβ induced ERK signalling in promoting EMT^36,100,101^^.^ GSK-3β suppresses EMT through SNAIL degradation, while AKT stabilises SNAIL from GSK-3β degradation promoting EMT. The combination of paths leading from TGFβ to SNAIL with varying signs, including both positive and negative interactions, is a result of the intricate network architecture inferred from experimental literature. The overall activation of SNAIL is determined by the collective effect of these combined interactions. Thus, SNAIL was observed to be a prominent player of EMT in MBC.

Similarly, ZEB, also a Zinc finger transcription motif comprising of ZEB1 and ZEB2 are important regulators of TGFβ induced EMT in breast cancer cells (Supplementary Fig. 8). Primarily regulated by SNAIL, ZEB was known to play a crucial role in maintaining the mesenchymal phenotype during the process of EMT^91,102–104^. ZEB recruits either YAP/AP-1 or CTBP along with other co-factors, which act as activators or repressors of mesenchymal or epithelial genes, respectively^105^. ZEB interacts with factors like OVOL2, GRHL2 in a mutually inhibitory loop resulting in multiple hybrid states^104,106,107^. Further, the presence of mutual inhibition between ZEB and GRHL2, as well as ZEB and miR-145, results in a hybrid with enhanced stability. Together, SNAIL and ZEB regulate the expression of the immune modulatory gene PDL1 in moderating the process of EMT^91^. In addition to SNAIL and ZEB families, the TWIST family of transcription factors plays an important role in the process of EMT and metastasis (Supplementary Fig. 8). Regulated through several SMAD-independent signalling pathways, TWIST is induced by AKT, NF-kB, SNAIL, ZEB. TWIST in turn activates FOXC2, AKT (though double positive feedback loop), LTGFβ, RHOC in regulating the expression of E-Cadherin thus modulating EMT. Thus, all the signalling upstream assists the key transcriptional factors in fine-tuning the process of EMT by either repressing epithelial phenotype-associated genes or by promoting the mesenchymal phenotype-associated genes.

Furthermore, various studies support that the augmentation of tumour invasion and metastasis is centred around autocrine TGFβ signalling mechanism^14,32,103,108^. TGFβRIII (beta glycan receptor family) a ubiquitously expressed coreceptor of TGFβ regulates the breast cancer progression and metastasis by either sequestering TGFβ or by interfering with PTEN inhibition of LTGFβ^32,109^. Above all, TGFβ signalling upstream also regulates the expression of matrix metalloproteinases (MMPs) (Supplementary Fig. 9)^64,110,111^ which are mainly involved in the cleaving of the E-Cadherin/β-Catenin tight junctions initiating the migratory and invasive phenotype of breast cancer cells as shown in Fig. 1. The progression of metastatic breast cancer in TGFβ simulated cells has also been linked to the expressions of stemness markers CD44^+^/CD24^−^ during the early stages of breast cancer^112^. Cells with an increased expression of CD44^+^/CD24^−^ further express high levels of EMT-associated genes thus promoting cell invasion and metastasis.

### RNAs in TGFβ induced MBC

TGFβ signalling regulates multiple RNAs through SMAD-dependent and SMAD-independent pathways during the emergence of MBC (Supplementary Fig. 10). Specifically, TGFβ regulates miR-190, miR-23a, miR-182, miR-106b in SMAD-dependent manner, promoting invasion and metastasis of breast cancer^113–116^. Regulation of miR-181a, miR-155, miR-21, miR-615, miR-1, by TGFβ suppresses EMT by altering the activity of SMADs^35,117–119^. Additionally, p53 plays an important role in regulating the expression of various miRNAs in maintaining the expression of epithelial phenotype^120^. Some of these miRNAs (miR-200, miR-34, miR-203, miR-204) are part of mutually inhibitory feedback loops with major mesenchymal markers ZEB1/2, SNAIL/SLUG which suppress EMT by preserving the E-Cadherin expression. Mechanistically TGFβ downregulates these miRNAs through SNAIL/SLUG, ZEB1/2 which actively target the genes involved in cell motility and invasion (Supplementary Fig. 10).

Although the majority of miRNAs are downregulated during malignant transformation, some miRNAs such as miR-520c, miR-133b, miR-153, miR-145 are observed to be elevated in the presence of TGFβ signalling which can negatively affect its signalling at the receptor level. On the contrary, TWIST-induced miR-10b has a context-dependent role in inducing EMT in different breast cancer cells^121–124^. Similarly, several other RNAs including miRNAs, mRNAs, LncRNAs were observed to be expressed in TGFβ induced EMT in breast cancer. Despite the evidence of several RNAs being expressed in TGFβ induced EMT of breast cancer cells, the mechanism of how several RNAs are regulated remains unknown.

### Tangled crosstalk and downstream response

The molecular classification of breast cancer cells is mainly based on the expression of oestrogen receptor (ER^+/−^), Progesterone receptor (PR+/–) and HER (+/-) receptors^125^. Stimulation of TGFβ triggers downstream signalling cascades, regulating HER status in breast cancer cells and enhancing the process of EMT, as depicted in Fig. 1. Wang et al.^126^, have reported the activation of ErBb3(HER3) signalling through TGFβ induced regulator TACE(ADAM17) which promotes EMT through PI3K signalling^126^. HER2 signalling through TGFβ induced TWIST and YB1 further regulates the expression of NFKB through STAT3 in modulating EMT^127–129^. In normal cells, ER-α exerts inhibitory effects on TGF-βRII and SNAIL through MTA1, contributing to the maintenance of epithelial structures. However, in malignant cells, TGF-β regulated SNAIL exhibits a feedback mechanism and inhibits ER-α, promoting the invasiveness of breast cancer cells^81,99,130^. Activation of TNF-α by p38 acts in parallel with TGFβ regulated TRAF in promoting transcription factor NF-kB regulating the process of EMT^101^. TGFβ signalling works collectively with other signalling axis in augmenting the process of EMT. These include avβ6 integrin, β3 integrin, UPA, IL-6, Wnt, EGFR (Supplementary Table 1). Additionally, TGFβ signalling is also observed to regulate several proinvasive genes (IL-8, IL-17, IL-11), cell cycle regulators (Cylin G2, p21, p27, p15, p53, p63) and stemness markers (CD24, CD44) in both SMAD-dependent and independent pathways. Recent studies have revealed a positive correlation between EMT and Androgen Receptor (AR) signalling influenced by ER signalling, specifically within the Triple-Negative Breast Cancer (TNBC) subtype^131^. Consequently, the induction of EMT in breast cancer cell lines by TGFβ and the cross-regulation observed underscores the distinct molecular composition of TNBC. Thus, TGFβ works in concert with other regulators and signalling pathways in moderating the EMT in breast cancer.

### Dynamic analysis of pathway Map of TGFβ induced EMT in MBC

**Fig. 3:**
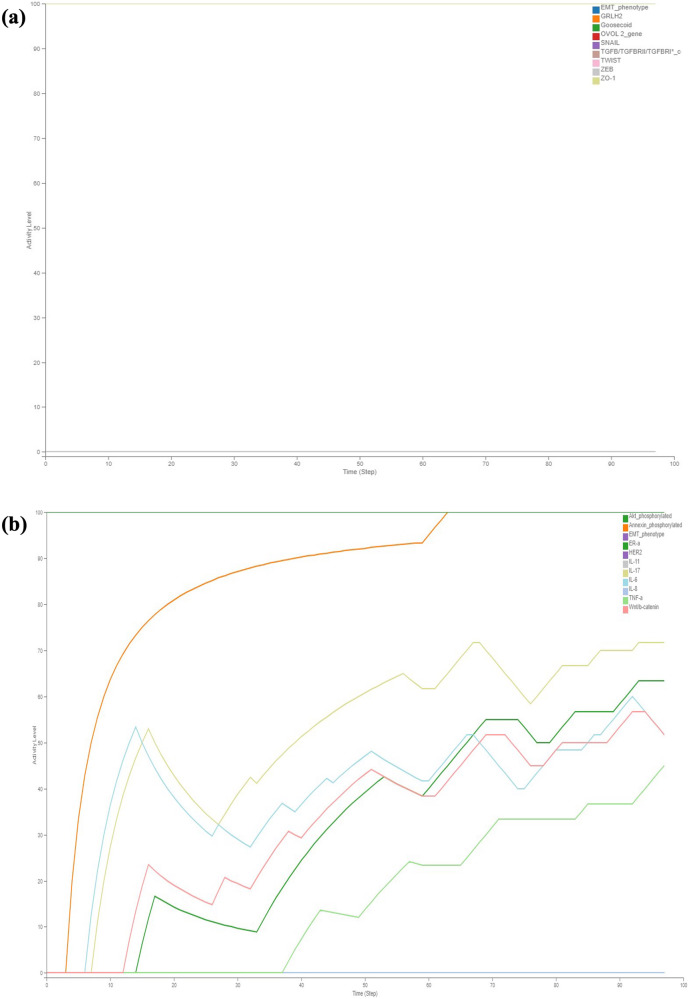
Discrete dynamic modelling and analysis of the assembled MBC map illustrating the activity of various EMT factors and receptors regulating them using Cell Collective in the absence of stimulus TGFβ. **a** The activity of all the mesenchymal markers was observed to be at their minimal levels and the epithelial markers were observed to be at maximal levels, **b** The activity of the receptors associated with epithelial phenotype remained at their maximum while the receptors associated with mesenchymal phenotype remain at their minimal level. The observation of moderate activity in Akt signaling and proinvasive genes suggests the presence of cross-regulation among the signaling molecules.

**Fig. 4:**
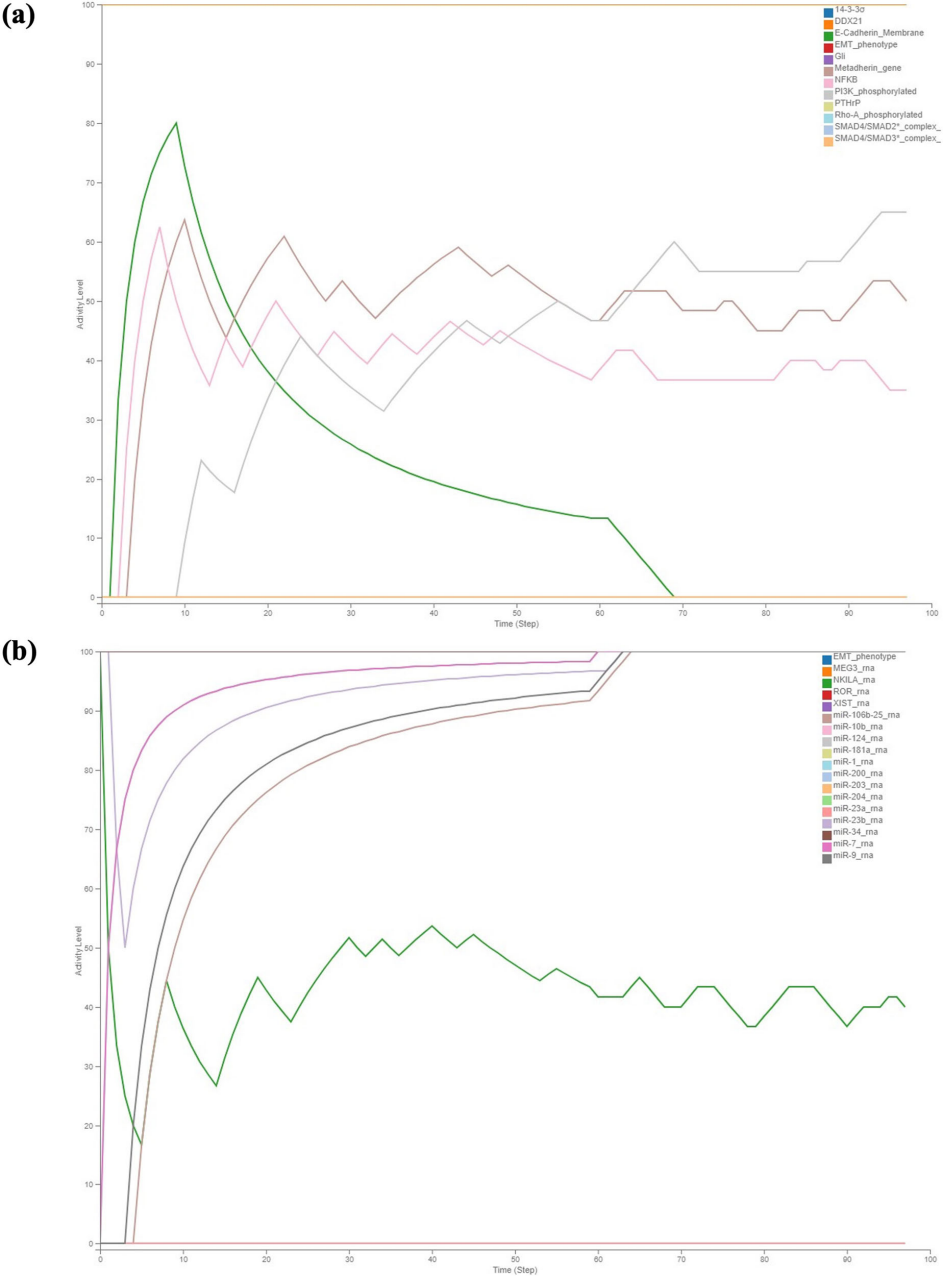
Discrete dynamic modelling and analysis of the assembled MBC map illustrating the activity of various key signaling pathways of EMT and miRNAs using Cell Collective in the absence of stimulus TGFβ. **a** The regulators involved in modulating the expressions of EMT markers exhibit moderate to minimum activity levels over time, with the notable exceptions of NF-kB, PI3K, and Metadherin regulators, **b** Epithelial miRNAs show maximum activity levels, while miRNAs regulating the mesenchymal phenotype were observed to exhibit minimum activity.

The pathway map of TGFβ induced EMT in MBC constructed using Cell Designer V 4.4 was stored as SBML file. The resulting SBML-Process Description file was converted into a Boolean inference model (SBML-*Qual*) utilising CaSQ. The executable SBML-*Qual* file obtained from CaSQ was imported into Cell Collective to capture the system dynamics during the process of MBC (Supplementary Information). To evaluate the dynamics, an epithelial state of the model was initialised with TGFβ^−^. The phenotypic characterisation was performed by the status of the nodes SNAIL, ZEB, TWIST, ZO-1, Goosecoid and EMT. These nodes are controlled by various epithelial and mesenchymal regulators like miR-200, miR-34, p53, PI3K, OVOL2, GRHL2, NF-kB, HER and ER towards maintaining the adherent cell-cell junctions (E-Cadherin/β-Catenin) upon TGFβ stimulation. Hence the regulation of these molecules results in the epithelial or mesenchymal or hybrid phenotypes. In the absence of TGFβ (Fig. 3a), the activity levels of most EMT markers and their regulators do not show any change in their activity*,* i.e., all the epithelial markers are at maximum activity levels and mesenchymal markers at minimum activity levels. However, some pathway regulators like PI3K, Akt Signalling, MTDH, NF-kB, Proinvasive genes like IL-11, IL-17, IL-6, IL-8, TNF-a, Wnt that stimulate the activity of EMT markers were observed to show low to moderate activity levels (Figs. 3b and 4a). E-Cadherin and β-catenin membrane levels were observed to be at their low activity suggesting the integrity of cell-cell junctions in the absence of TGFβ. From Fig. 3b it can be observed that other signalling pathways HER, ER were not regulated in the absence of TGFβ. Similarly, all the epithelial miRNAs involved were observed to be at their maximum activity and mesenchymal miRNAs at their minimum activity with the exemption of a few miRNAs (Fig. 4b). These findings shed light on the complex regulatory dynamics underlying the EMT process and provide valuable insights into the behaviour of key molecular components in the absence of TGFβ signalling.

**Fig. 5:**
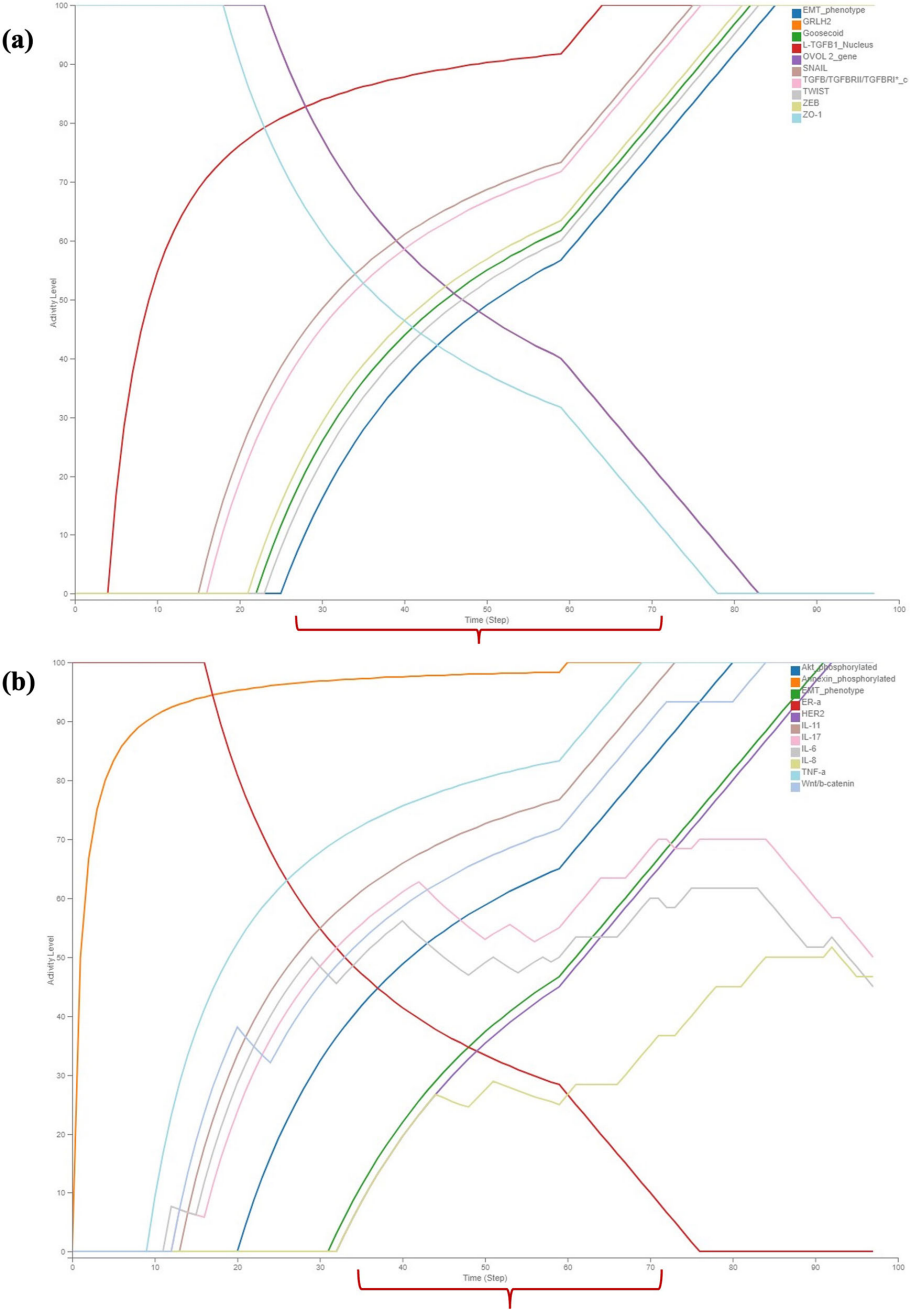
Discrete dynamic modelling and analysis of the assembled MBC map activity of various EMT factors and receptors regulating them using Cell Collective in the presence of stimulus TGFβ. **a** Shows maximum activation of core transcription factors of EMT like ZEB, SNAIL, and TWIST along with the upregulation of the EMT marker Goosecoid in regulating EMT. Simultaneously, the activity of the epithelial gene ZO-1 was observed to be reduced. **b** Induction of the other signaling pathways HER, TNF-α, Wnt, Akt and proinvasive genes along with the concurrent loss of ER-α was observed. Notably, the coexistence of epithelial and mesenchymal regulators (i.e., hybrid phenotype) was observed in the presence of TGFβ.

**Fig. 6:**
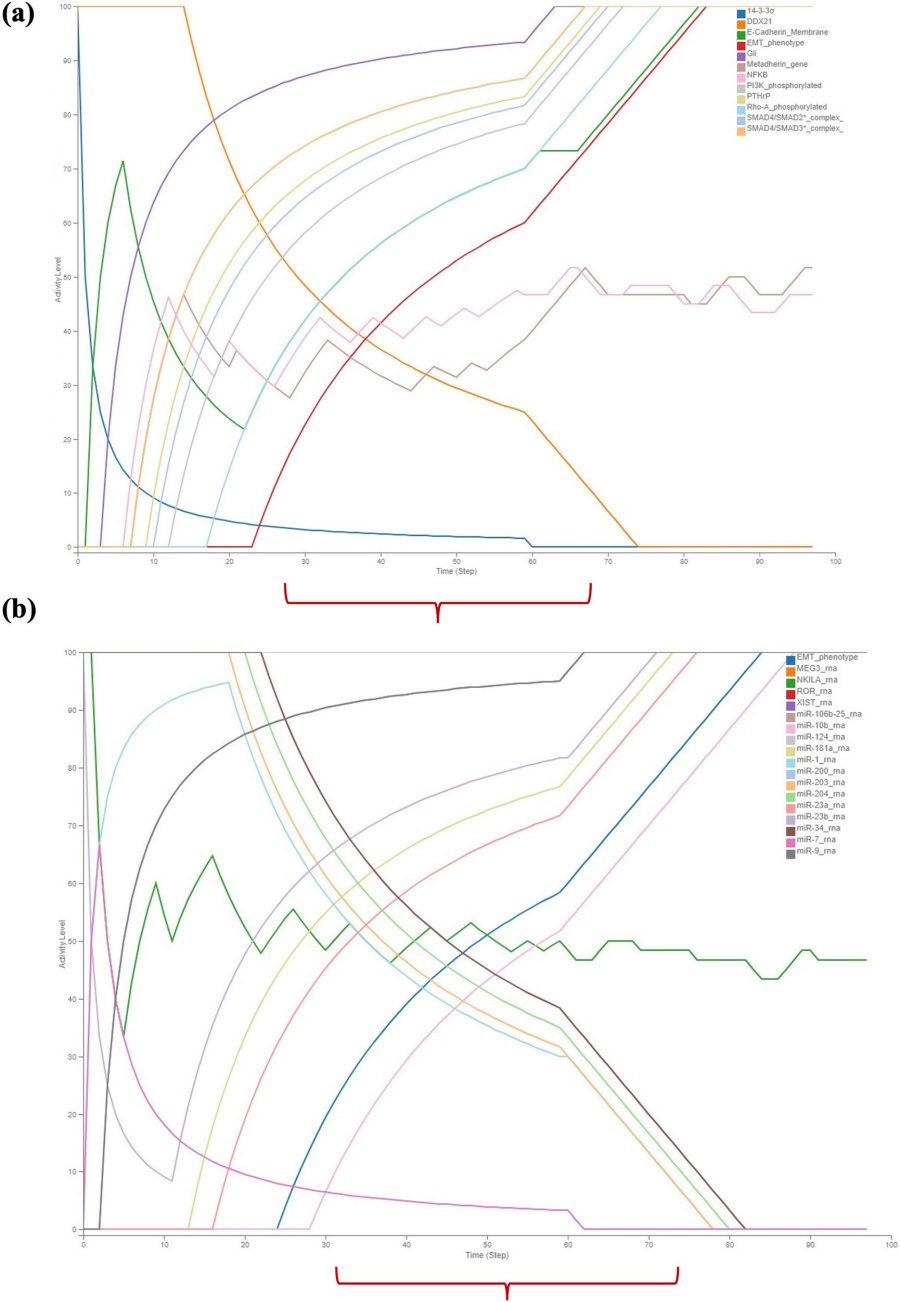
Discrete dynamic modelling and analysis of the assembled MBC map illustrating the activity of various key signaling pathways of EMT and miRNAs using Cell Collective in the presence of stimulus TGFβ. **a** The regulators responsible for maintaining the epithelial phenotype were observed to exhibit reduced activity, while those governing the mesenchymal phenotype were observed to display increased activity, driving EMT. **b** The activity of miRNAs governing epithelial phenotype was observed to decrease, while that of mesenchymal miRNAs was increased, with a few exceptions; Notably, the coexistence of epithelial and mesenchymal regulators (i.e., hybrid phenotype) was observed in the presence of TGFβ.

When TGFβ is active, all the regulators involved in modulating the EMT were observed to be expressed at their maximum activity levels (Figs. 5 and 6). This indicates that the presence of active TGFβ signalling induces a coordinated upregulation of the regulatory components that orchestrate the EMT process. These pathway components, exhibit peak activity levels, leading to the promotion of EMT and subsequent changes in cell behaviour, such as increased cell motility and invasion. Stimulation of TGFβ by setting its input to 100% activated its receptor complex, initiating downstream signalling through both the SMAD signalling pathway and SMAD-independent pathways, as shown in Figs. 5a and 6a. The activation of these pathways resulted in an increase in the activity levels of E-Cadherin_membrane and β-Catenin_membrane, indicating the disruption of cell-cell junctions (Fig. 5a). Similarly, all the epithelial miRNAs involved were observed to be at their minimum activity and mesenchymal miRNAs at their maximum activity with the exception of few miRNAs (Fig. 6b). The LncRNA NIKLA, in conjunction with NF-kB, was observed to exhibit oscillatory behaviour due to the presence of a negative feedback loop between these two nodes. This negative feedback mechanism leads to fluctuations in the activity levels of both LncRNA NIKLA and NF-kB, resulting in oscillations in their expression patterns over time. The oscillatory nature of this regulatory circuit may play a significant role in modulating cellular responses to TGFβ stimulus (Fig. 6b). The collective activity of all the regulators, including the TGFβ receptor complex, SMAD signalling pathway and SMAD-independent pathways modulate the activity of key EMT regulators. These regulators include SNAIL/SLUG, ZEB1/2, TWIST1, Goosecoid and ZO-1, as well as phenotype stability factors such as OVOL2 and GRHL2. (Fig. 5a). From Figs. 5a, 6a it is evident that the onset of EMT occurs when both EMT regulators and EMT markers reach their maximum activity levels. This observation suggests that the activation of EMT is tightly regulated and requires the simultaneous upregulation of key EMT regulators and markers. From Figs. 5, 6 coexistence of epithelial and mesenchymal markers was observed for a period of time indicating the hybrid phenotype. This suggests that the process of EMT is not advocated by a single regulator but by a group of regulators in action.

Supplementary Fig. 11 depicts the activity network of the comprehensive map assembled in the absence (Supplementary Fig. 11a) and presence (Supplementary Fig. 11b) of the stimulus TGFβ. The activity network provides a visual representation of the regulatory interactions and signalling pathways that are activated or suppressed under these two conditions. When TGFβ is absent (Supplementary Fig. 11a), certain regulators and pathways show low to moderate activity levels, while in the presence of active TGFβ (Supplementary Fig. 11b), all the regulators involved in modulating the EMT process exhibit their maximum activity levels. This comparison highlights the dynamic changes in the network and the impact of TGFβ signalling on the regulation of EMT-related processes. Thus, the activation of TGFβ initiates a cascade of molecular events, ensuring a synchronised and potent induction of EMT, contributing to cancer metastasis.

### Network analysis using Cytoscape

**Fig. 7:**
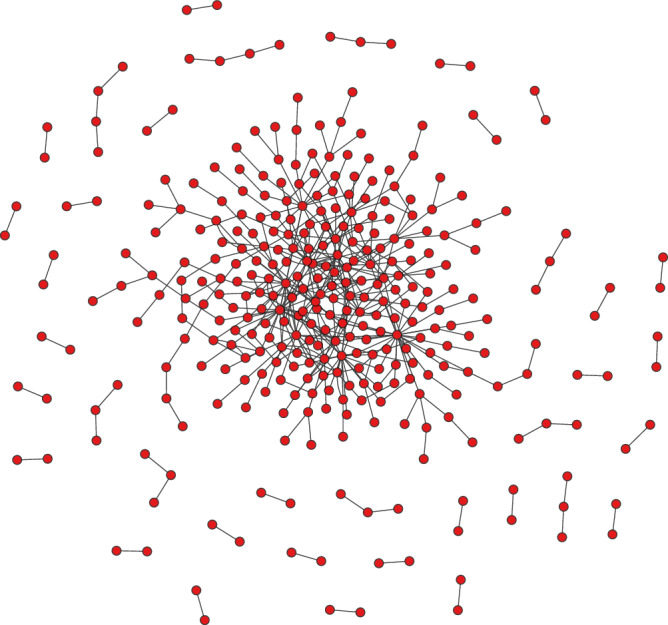
Visualisation of the assembled map using Cytoscape. The assembled map of metastatic breast cancer (MBC) visualised using the Compound Spring Embedder (CoSE) layout within the Cytoscape. This visualisation highlights the intricate relationships within the MBC network. The network is visualised as a complex structure comprising a single centrally connected network (graph) and 37 separate subgraphs.

Basic network analysis was performed to explore the properties of the map developed. XML and CSV tabular formats of MBC map were imported into the Cytoscape V3.10 and analysed further using the inbuilt plugin Network analyser for topological metrics. The XML file imported comprises of 733 nodes and 1023 interactions connected as one core component (Supplementary Fig. 12). Whereas the CSV file imported into Cytoscape has 340 nodes and 426 interactions which was utilised as input for further analysis (Fig. 7).

Topological analysis using the built-in plugin Network analyser of Cytoscape revealed that the network consists of 38 connected components corresponding to connecting subgraph with a core subgraph and 37 smaller subgraphs (Fig. 7). It was observed that all node degree distributions follow the power law, showing that the networks are scale-free (Supplementary Fig. 13). Topological parameters obtained for both directed and undirected graph of the MBC network assembled in this study were tabulated (Table 1). Each node in MBC network has an average of ~2.9 neighbours. The network density is 0.015 for the directed graph which implies that the network is dense. Directed graphs indicate the flow of information thus consider both the presence of edges and their direction. The clustering coefficient of the MBC network was 0.037 which implies that there are very few links between the neighbours of the node in the network.

**Table 1:** Simple topological parameters obtained with the network analyser tool of Cytoscape for the assembled map of MBC

Property	Directed	Undirected
Number of nodes	340	340
Number of edges	426	426
Avg. number of neighbours	2.418	2.847
Network diameter	14	14
Network radius	1	7
Characteristic path length	5.375	4.786
Clustering coefficient	0.015	0.037
Network density	0.004	0.011
Connected Components	38	38
Multi-edge node pairs/network heterogeneity	15	1.143
Number of Self Loops/network centralisation	0	0.092
Analysis time (sec)	1,693,824,729.066	0.061

There are 15 multi-edge node pairs that provide information about the frequency of multiple connections between neighbouring nodes in the network. The longest shortest path between any two nodes, i.e., the network diameter was 14 suggesting that the signal originating from TGFβ ligand-receptor complexes in the membrane can reach a significant portion of the network within 14 steps. The characteristic path length of the network, which signifies the average distance between connected nodes, was approximately 4.7. This indicates that the response to a signal and its propagation can occur with moderate efficiency within the network. The highly connected nodes with a large number of incoming and outgoing edges indicate the network’s tendency to have hub nodes (These are referred to as ‘multi-edge node pairs’ in directed graphs and ‘network heterogeneity’ in undirected graphs).

**Fig. 8:**
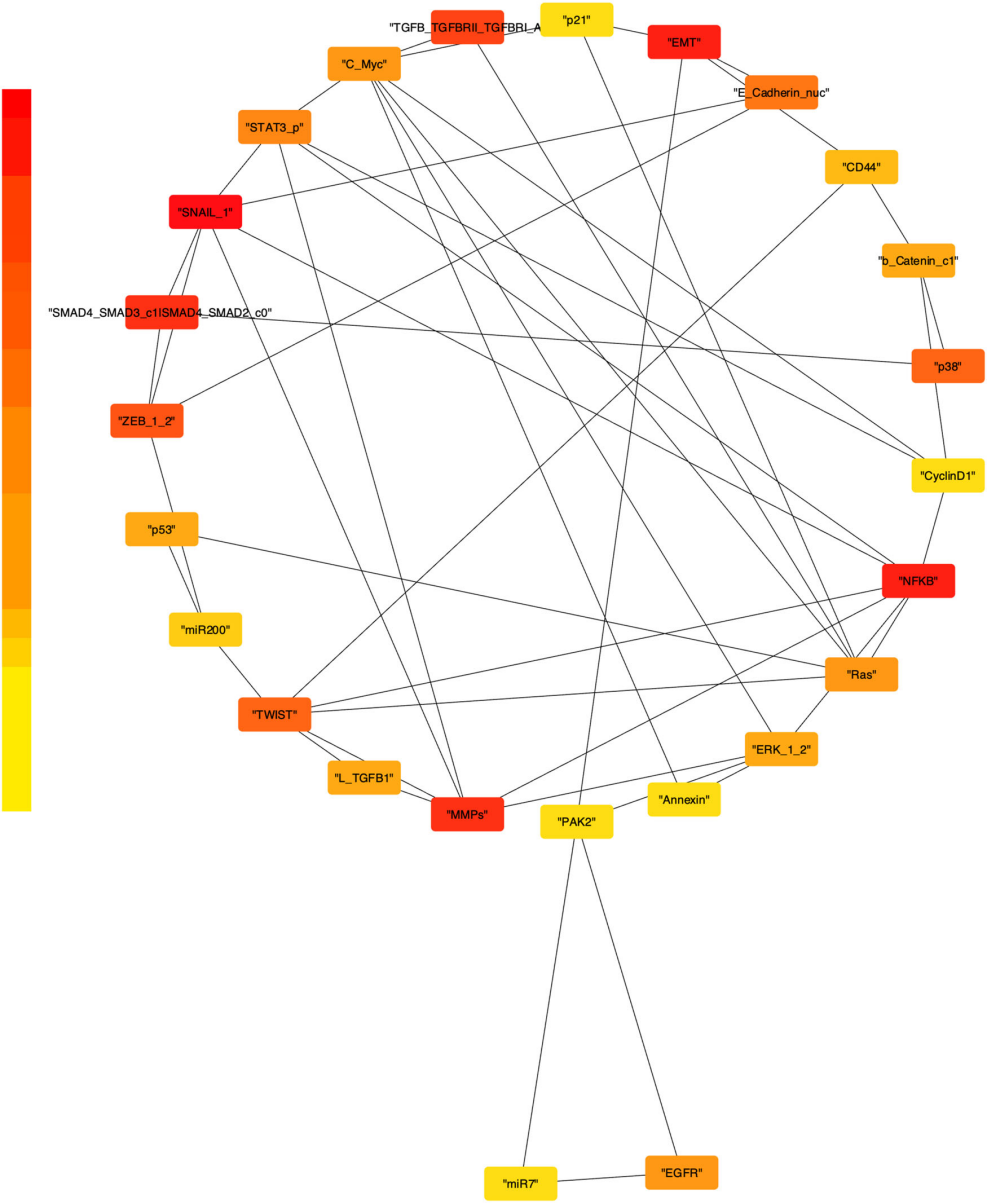
Hub genes identified using Cytohubba. Top 25 influential genes in the assembled MBC as identified by the cytohubba tool employing the MCC (Maximal Clique Centrality) method. Hub genes are those genes that are members of the largest cliques within the network. The level of importance for these hubs was visually represented using a colour scale from highly significant to significant genes (red to yellow).

Cystoscape plug-in Cytohubba-based analysis of the converted network (Fig. 7) resulted in the identification of the 25 most influential genes (Fig. 8) of the MBC map assembled (Fig. 1). The gradient from yellow to red implies significant to the highly significant genes. SNAIL, NF-kB, MMPs, SMAD4/SMAD2, SMAD4/SMAD3 complexes, TGFB_TGFBRII_TGFBRI_A complex, ZEB1/2, TWIST, p38, E-Cadherin, MDM2, STAT3, RAS, C-Myc, EGFR, ERK, β-Catenin, p53, L-TGFB1, CD44, miR-200, Annexin, Cyclin D1, PAK2, miR-7 and p21 were these 25 most influential genes. These Hub genes represent key players within the MBC map. All the hub genes identified were found to be driver/ oncogenes/tumour suppressor genes promoting MBC^58,69,86,100,132^. Most of the identified hub genes were observed to contribute to invasiveness (Supplementary Table 2). Supplementary Table 2 provides a detailed list of identified hub genes. Some of these genes are also part of interesting network motif architecture that plays a crucial role in driving emergent behaviour.

### Transcriptome-based validation

The prognostic significance of identified hub genes in MBC and their clinical relevance was further assessed through survival analysis. For survival analysis, the invasive breast cancer patients (BRCA) from TCGA and GTEx databases were evaluated using the GEPIA database (Supplementary Figs. 17 and 18). SNAI1, NF-kB2, KRAS, MMP7, MAPK1, LTBP1, EGFR, TP53, PAK2, and CDKN1A (p21) exhibited significant prognostic differences in their overall survival with *p* < 0.05 (Table 2). Additionally, LTBP1, SNAI1, STAT3 were observed to be significant in terms of disease-free survival. Notably, low expressions of CDKN1A, TP53 are associated with better overall survival, while high expression of LTBP1 is associated with better overall survival respectively. Moreover, High expressions of LTBP1, SNAI1 are observed to be associated with better disease-free survival.

**Table 2:** Transcriptome analysis of the hub genes

Gene	Overall survival	Disease-free survival
TGFBR1	0.36	0.26
TGFBR2	0.52	0.78
TGFB1	0.34	0.77
LTBP1	0.069*	0.059*
SNAI1	0.017*	0.062*
NFKB1	0.96	0.84
NFKB2	0.042*	0.32
SMAD2	0.33	0.55
SMAD3	0.97	0.14
SMAD4	0.42	0.57
MMP2	0.32	0.82
MMP3	0.93	0.5
MMP7	0.041*	0.82
MMP9	0.2	0.8
ZEB1	0.44	0.99
ZEB2	0.27	0.37
TWIST1	0.16	0.61
TWIST2	0.82	0.7
MAPK14	0.66	0.43
CDH1	0.4	0.99
STAT3	0.85	0.035*
KRAS	0.031*	0.78
HRAS	0.28	0.55
MYC	0.61	0.48
EGFR	0.091*	0.61
MAPK3	0.37	0.12
MAPK1	0.029*	0.95
TP53	0.073*	0.67
CD44	0.61	0.43
PAK2	0.075*	0.34
CDKN1A	0.058*	0.9
ANXA2	0.14	0.55
CTNNB1	0.31	0.27
CCND1	0.75	0.16

Expression levels of the hub genes and their correlation with patient survival in invasive breast carcinoma (BRCA) obtained from GEPIA database along with *p*-value is shown. Genes with significant *p*-values (*p* < 0.05) were indicated by *. They indicate the genes that exhibit significant associations with patient survival.

**Fig. 9:**
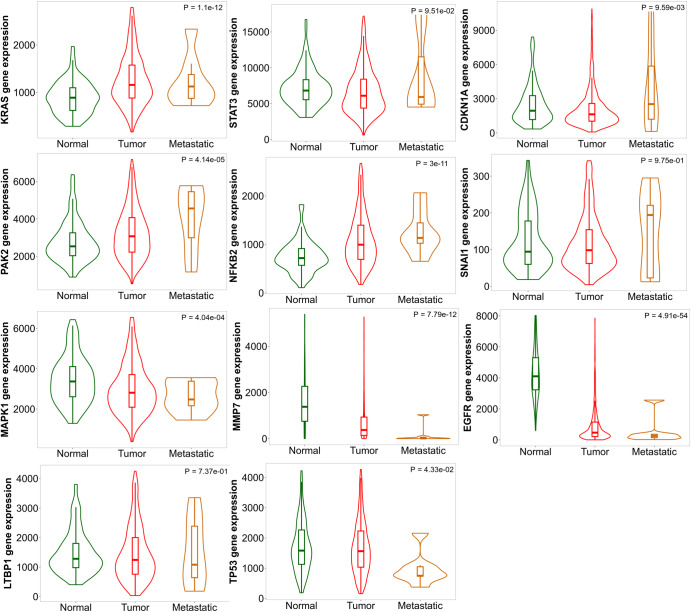
Transcriptome-based analysis of the significant hub genes. Violin plots illustrating the expression patterns of prognostically significant hub genes identified in invasive breast carcinoma. These plots compare the gene expression across tumour, normal, and metastatic RNA-seq data. The bars within the plots represent the proportions of samples, and the statistical analysis was conducted based on Kruskal–Wallis test for the regulators with log *p* < 0.05 from survival analysis (Table 2).

Further, the significance of these prognostically relevant genes was evaluated by comparing the RNA-seq expression patterns between normal, tumour, and metastatic samples. TNMplot database was utilised for this analysis and the statistical significance was determined based on Kruskal–Wallis test (Fig. 9). The expressions of KRAS, STAT3, CDKN1A, PAK2, NFkB2, SNAI1, were observed to be significantly higher in metastatic samples compared to tumour and normal samples. However, the expression of MAPK1, MMP7, EGFR, LTBP1, TP53 was observed to be lower in the metastatic samples compared to normal and tumour samples. This highlights the distinct gene expression states of prognostically relevant genes in metastatic samples compared to both tumour and normal samples.

## Discussions

In this work, a comprehensive map illustrating the TGFβ induced migration of breast cancer cells leading to metastatic breast cancer was compiled. The regulatory map illuminates the role of numerous individual regulators and their associated pathways activated because of TGFβ. The map comprises of both SMAD-dependent and SMAD-independent pathways regulating EMT in MBC. These diverse regulatory components and their interconnected pathways highlight the complexity of the signalling mechanisms involved in promoting MBC. This network perspective allows viewing the complex phenomenon of EMT as a function of all the regulators and their interplay involved which provides a unique view of the emergence of MBC.

TGFβ plays a crucial role in orchestrating various regulators and regulatory networks, triggering a cascade of molecular events contributing to cell migration, invasion, and ultimately metastasis^11,21^. Several experimental studies have identified the role of TGFβ induced regulators and their mechanistic roles in regulating EMT and cancer metastasis. Activation of the developmental process of EMT is fundamental for several cellular functions^6,133^. Within the spectrum of EMT, Type I and type II EMT are associated with the physiological functions of a cell, including organ development, embryogenesis, organ fibrosis, and tissue development, whereas type III EMT is implicated in the pathophysiological functions of a cell, contributing to the progression of neoplasia and metastasis^6,133^. The scope of the comprehensive map assembled in this study was to enumerate such experimental information focusing particularly on the process of TGFβ induced EMT in metastatic breast cancer.

Similar comprehensive maps are available in literature focusing on the underlying complex molecular processes presenting a consensus view on the network-level processes and diseases. Examples of such focused studies include an illustration of cell cycle regulatory events^134^, EGFR signalling^135^, RB/E2F signalling^136^, G-protein coupled receptor signalling architecture^137^, Alzheimer’s disease map^138^, cancer signalling^139^, COVID-19 map^46,140^, atlas of inflammation resolution^141^, rheumatoid arthritis map^142^. Systems biology-based studies of molecular interaction networks of numerous biological processes have led to unique experimental studies that have revealed various molecular mechanisms^143–147^. This demonstrates the potential value of such disease maps and future prospects for such maps in designing experiments. The true potential of disease maps is yet to be realised.

The systems-level elucidation of TGFβ pathway map (Fig. 1) introduced in this work adds to this collection and provides systematic information of TGFβ induced EMT in the emergence of MBC. Lurking in these intricate signalling networks are key interactions and motifs that orchestrate the TGFβ stimulus in inducing EMT and cancer metastasis^104,148–151^. Exploring these underlying motifs is essential in understanding the contribution of TGFβ signalling activation of downstream effectors and transcription factors that drive the phenotypic changes associated with EMT and cancer metastasis.

Network modelling and analysis can explain the diverse contexts in which the TGFβ signal governs the process of EMT. Outcomes from the discrete dynamic modelling and simulation studies using Cell Collective have demonstrated that the model developed can reproduce the known biological dynamics of TGFβ signalling in metastatic breast cancer. The constitutive presence of TGFβ was sufficient to regulate the SMAD signalling, HER signalling, TNF signalling, Wnt signalling, ER-α signalling thereby regulating EMT-associated regulators and markers. These include SNAIL, ZEB, TWIST, ZO-1, E-Cadherin_Membrane, L-TGFβ which further orchestrate EMT. It should be noted that these regulators and signalling pathways have been individually implicated in EMT (Supplementary Table 1). The systems-level impact of TGFβ in promoting EMT is not attributable to TGFβ alone but is dependent on the intricate interplay within the entire system that TGFβ signal participates with (Steinway et al.,^152^). Gaining insights into the dynamic behaviour of the TGFβ signalling pathway is crucial in identifying molecular events that drive cancer progression and metastasis.

The topological analysis of the disease map has shown scale free nature of the MBC map assembled. The topological properties of the cancer networks, including one connected component, moderate connectivity, network density, and efficient signal propagation, suggest a highly interconnected and information-rich network architecture. Thus, illustrating the complex structure and its potential to facilitate the dynamic signal transmission in deciphering complex disease mechanisms of EMT in MBC. Further, the topological analysis also assists in identifying the significantly influential nodes (hubs). 25 hubs genes of the MBC map thus identified as seen in Fig. 8 were characterised by their implications in the disease. Survival analysis using GEPIA, and expression validation in metastatic samples using TNMplot highlighted the prognostic relevance of TP53, LTBP1, NF-kB2, CDKN1A (p21), MMP7, KRAS, PAK2 based on the transcriptome data. The set of transcriptome analysis performed on the hub genes of the assembled map, validates their role in the context of cancer data available in public databases like TCGA, GTEx. Specific molecular biology-based experiments could assist in finding the mechanistic roles of these regulators and their impact in altering the pathways during the breast cancer progression.

Many publicly available pathway databases like Reactome^153^, KEGG^154^, STRING^155^, IPA^156^, GeneMANIA^157^ collate data from multiple experimental and omics studies. These databases provide insights into different aspects of cellular interactome at various levels. However, one of the key challenges lies in ensuring the exclusiveness of the specific molecular interactions within pathways. We have observed that the pathways obtained from STRING, Reactome gives an overview of the gene regulatory network for a given query, they may be a shortfall in various information including exclusive molecular mechanism, source of information, cross-regulation between the signalling molecules (Supplementary Figs. 14–16). The map (Fig. 1) derived from an extensive literature survey addresses various inconsistencies encountered within these pathway databases thereby offering a more accurate and reliable representation of the molecular process under study. This rigorous curation process along with the validation through logical model-based validation illustrates the authenticity and usefulness of the map by answering specific questions for further research and understanding of cancer metastasis. This comprehensive map of TGFβ induced EMT allows exploration of underlying mechanistic processes contributing towards the emergence of MBC.

In summary, through an extensive literature survey, a comprehensive regulatory network encompassing both TGFβ induced SMAD-dependent and SMAD-independent signalling pathways involved in MBC has been constructed. This has led to the identification and integration of a wide range of regulators and their interactions involved in the malignant transformation of epithelial cells in MBC. The map developed (http://35.174.227.105:8080/minerva/?id=Metastatic_Breast_Cancer_1) serves as a valuable knowledgebase, consolidating information from various sources and facilitating a deeper understanding of the complex regulatory mechanisms underlying MBC. While the complete molecular mechanism of MBC remains incompletely understood, this work provides valuable insights into the regulatory networks governing TGFβ induced MBC. Further to validate, the map assembled was translated into a logical model and simulations of which have captured the known experimental outcomes of TGFβ induced Epithelial to Mesenchymal Transition in MBC. The identified hub regulators of the map and their transcriptome-based analysis have confirmed their role in breast cancer metastasis. By shedding light on the complex interplay of signalling pathways in MBC, this work contributes to advancing the knowledge and potential therapeutic approaches for combating MBC. Ultimately, the findings of this study have the potential to guide further research, promote data-driven analysis, and inspire new strategies for personalised cancer therapy.

## Methods

### Curation of EMT network

**Fig. 10:**
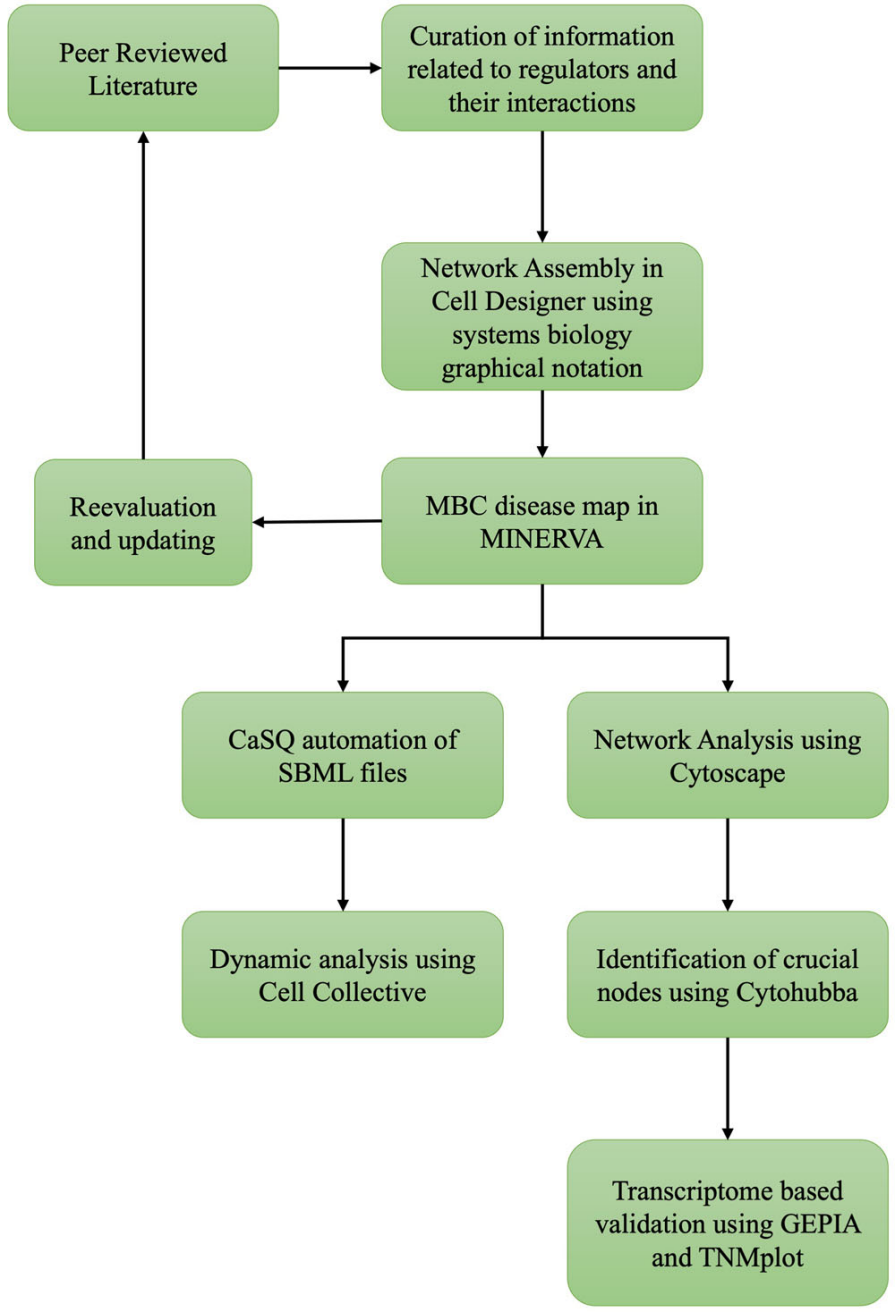
Workflow for the development and analysis of a comprehensive disease map of TGFβ induced EMT in MBC. This figure illustrates the workflow for developing a comprehensive disease map of the signaling pathways involved in TGFβ induced epithelial-to-mesenchymal transition (EMT) in metastatic breast cancer (MBC). The development of a map is an iterative process involving the integration of information from various experimental studies found in the scientific literature. The final map is made publicly available through the MINERVA platform and is subjected to further validation through various analyses.

Metastatic breast cancer (MBC) is modulated through various pathways and diverse regulators stimulated by TGFβ signalling. Several in vitro and in silico studies have been performed previously to identify the regulators and their mechanisms involved in MBCs. The overall approach followed for developing the map (Fig. 1) is shown in Fig. 10. Disease map of TGFβ induced EMT in breast cancer was constructed by performing an extensive literature survey that accounts for various regulators involved in disease emergence. The survey primarily focused on the experimental studies that identify the regulators involved in TGFβ induced EMT. Specifically, genes, proteins, RNAs other receptor signalling pathways regulated by TGFβ or regulating TGFβ and their interactions between them in inducing EMT were explored. The focus was majorly on TGFβ and its association with EMT in metastatic breast cancer. The information gathered largely consists of studies from the human cell lines-based experiments with a few exceptions based on mouse model-based experiments. The literature obtained is thoroughly cross-checked multiple times for the role of the regulators and their specific mechanism of action. HUGO nomenclature was acquired (https://www.genenames.org) for all the regulators involved in the disease map to avoid uncertainty in terminology.

The MBC regulatory network map was assembled using Cell Designer V 4.4 (http://celldesigner.org/) and follows the graphical notation system proposed by Kitano et al.^158^. Cell Designer supports the systems biology graphical notation (SBGN) and the constructed map can be exported into systems biology markup language (SBML) format for preferred computational analysis. MINERVA platform^159–161^ was utilised to create a navigation-friendly web version of the assembled MBC map. It allows for visual exploration, analysis and management of assembled MBC Map (Fig. 2). It also provides automated annotation (Pubmed, Ensembl, Uniprot, RefSeq, miRbase, HGNC ID) of species and reactions of the networks.

### Insilco simulations

To further capture the dynamic behaviour of TGFβ induced EMT in metastatic breast cancer Insilco, Boolean network modelling and analysis were performed. Boolean models are well suited to handling large sizes of data described in molecular interaction maps where there is a lack of kinetic data^162^. Boolean formalisms are scale-free, simplest forms of logical models where nodes represent the species (Protein, Genes, RNA, Complex) and edges (Activation, Inhibition, Association, Dissociation) represent the interactions between them. Each node is associated with a binary variable determining its qualitative level (0 False or 1 True) and its ability to influence its target (0 Inactive or 1 Active). The state of each node (Regulator) is determined by the states of neighbouring nodes (Regulators) represented by a set of regulatory functions that updates with time. Regulatory functions for each node (Regulator) were formulated depending on their upstream nodes (regulators) using logical operators AND, OR, NOT and are known to produce recognisable biological outcomes as obtained from experimental literature. The state of each node remains the same or changes depending on the regulatory function that updates with time. Thus, the state of a regulator follows a dynamic trajectory based on discrete variables 0 through 1. The updating of the rules can be synchronous i.e., all the nodes are updates at the same time, or asynchronous i.e., where only one node can be updated every time^163–165^.

To perform the logical modelling and analysis of the TGFβ induced EMT in MBC, the SBML file of the MBC interaction map obtained from the Cell Designer was converted into an SBML-*Qual* file using CaSQ, (https://github.com/soli/casq) to obtain a Boolean model. SBML-*Qual* is an extension of Systems Biology Markup Language (SBML) Level 3 Standard, represented for the qualitative models of biological networks^166^. The SBML-*Qual* file obtained is compatible for further analysis with the modelling tool Cell Collective (https://cellcollective.org/). The SBML*-Qual* file was imported into Cell Collective, a web-based modelling platform to perform real-time stimulations. Regulators of the interaction map are inferred as internal and external components by the Cell Collective under ‘Model’ tab along with a panel for regulatory expression for each selected regulator. The dynamics of the TGFβ induced MBC were performed using the ‘Simulations’ tab on Cell Collective using asynchronous update under epithelial initial environment conditions. The outcomes are measured based on the activity of EMT along with several epithelial and mesenchymal regulators using the ‘simulation graph’ panel under ‘Simulations’ tab.

### Topological analysis

The MBC map assembled (Fig. 1) was imported into Cytoscape for topological analysis using the built-in plugin Network Analyzer^167^. Cytoscape is an open-source platform for visualising and integrating molecular interaction networks with annotations, gene expression profiles and other data types. The MBC network was first analysed as an undirected network to obtain the overall degree distribution, then as a directed network to obtain the indegree, outdegree distribution and other topological properties. Subsequently, to obtain the influential genes of the MBC map Cytoscape plugin Cytohubba was utilised^168^. Specifically maximal clique centrality (MCC) of Cytohubba was used to identify the potential hub genes involved and to obtain the subnetwork.

### Transcriptome-based analysis and validation

To validate the prognostic relevance of identified hub genes, survival analysis was performed with invasive breast cancer (BRCA) datasets using GEPIA database^169^. GEPIA performs the Kaplan–Meier survival analysis by analysing the relative expression of hub genes. GEPIA comprises the data of the normal (112) and tumour (1085) samples from the TCGA and GTEx databases. For the analysis all the parameters were set to default values, with quartile cutoff. Overall survival and disease-free survival were evaluated using the Mantel-Cox test with a 95% confidence interval and Cox proportional hazardous ratio. A log *p*-value of less than 0.05 was considered as statistically relevant to identify the prognostically relevant genes. Further, the validation of the expression of prognostically relevant genes between tumour, normal and metastatic samples was analysed using the web platform TNMplot that integrates the transcriptome data from NCBI-GEO, TCGA, TARGET, GTEx repositories^170^. Specifically, RNA-seq data of invasive breast carcinoma was opted to compare the datasets to perform Kruskal–Wallis test. The combination of survival analysis using GEPIA and DE analysis using TNM plots could further help in coarse-graining and validating the prognostically relevant genes of the MBC map.

### Reporting summary

Further information on research design is available in the Nature Research Reporting Summary linked to this article.

### Supplementary information

Supplementary Information

Reporting summary

## Data Availability

All the files generated and analysed during the current study are available in the GitHub repository, https://github.com/gsb-sai/Metastatic-Breast-Cancer.
