# Supplementary material for: Comprehensive molecular interaction map of TGFβ induced epithelial to mesenchymal transition in breast cancer
Source: NPJ Syst Biol Appl. 2024 May 17;10:53. doi: 10.1038/s41540-024-00378-w (PMC11101644; doi:10.1038/s41540-024-00378-w)
Supplement: Supplementary file 1 — Supplementary Information [file 41540_2024_378_MOESM1_ESM.pdf]

Supplementary Information

Comprehensive Molecular Interaction Map of TGFβ Induced Epithelial to Mesenchymal Transition in Breast Cancer

Sai Bhavani Gottumukkala[1], Trivadi S Ganesan[2], Anbumathi Palanisamy[1] *,

[1]Department of Biotechnology, National Institute of Technology Warangal, India.

[2]Department of Medical Oncology, Sri Ramachandra Institute of Higher Education and Research, Chennai, India.

*Correspondence and requests for materials should be addressed to AP(anbu@nitw.ac.in)
Author's ORCID ID: 0000-0002-9076-5271; 0000-0002-4671-8099; 0000-0002-2870-6385

List of Contents

Supplementary Information : Links to Boolean Modelling and Map Developed - Minerva Database

Supplementary Figures 1-6 (Zone wise description of the map developed)

Supplementary Figures 7-18

Supplementary Tables 1-3

Supplementary References

Supplementary Information

MINERVA Database:

The assembled MAP of TGFβ induced EMT in MBC is available at : http://35.174.227.105:8080/minerva/?id=Metastatic_Breast_Cancer_1

The Boolean model of the map developed using cell collective can be found here: https://research.cellcollective.org/#b45902c2-3958-47e7-a1bb-2712dba8c453

The initial conditions used for the Dynamic model simulations were specified in simulations section under the tab 'Initial State' labelled as Epithelial.

Supplementary Figures

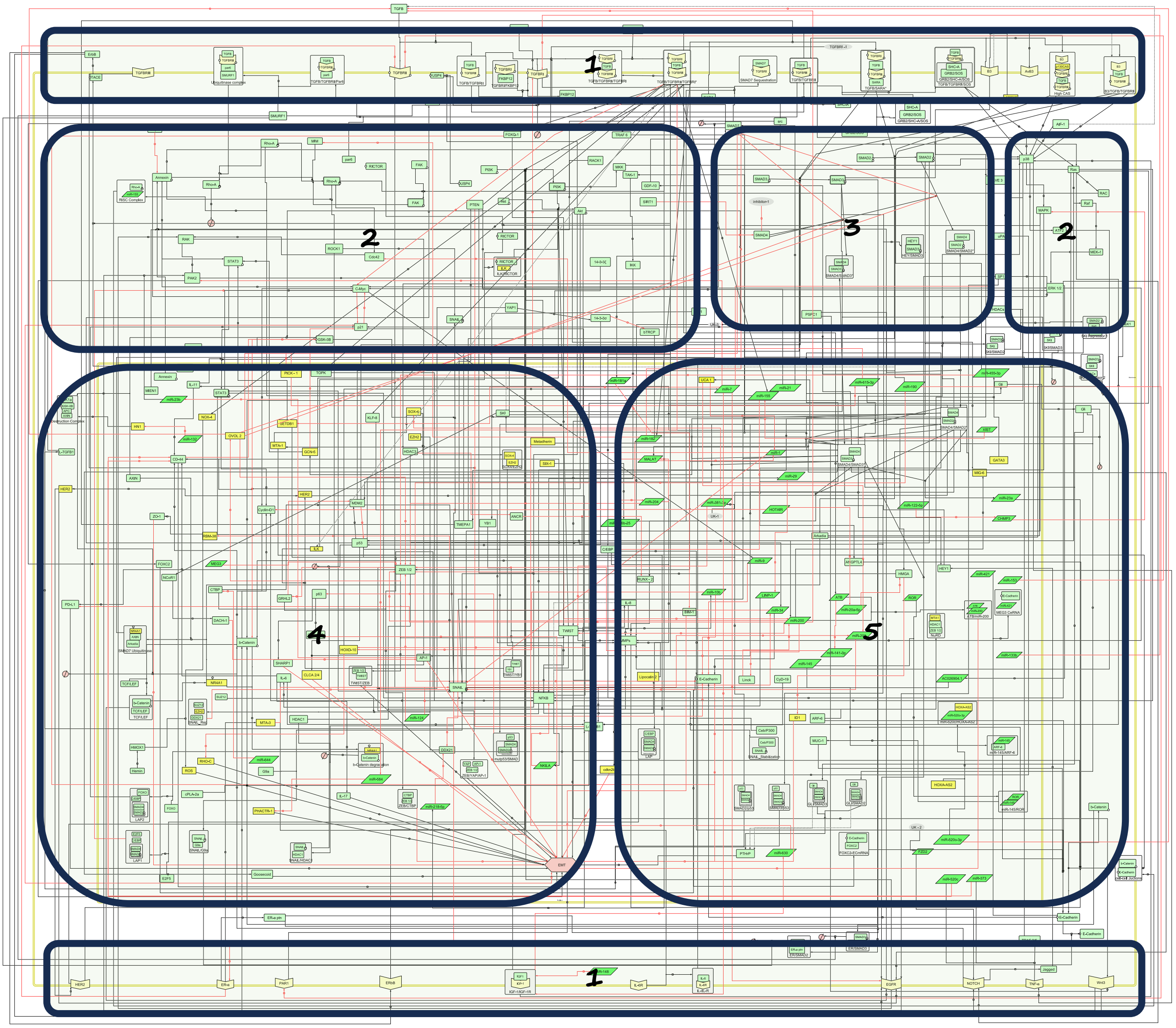

Supplementary Figure 1. The complete orchestration of TGFβ induced regulators and signaling pathways during EMT promoting MBC visualised in Zones

Zone	Regulators Involved
1	Cell Surface Receptors
2	SMAD Independent Pathways
3	SMAD Dependent Pathways
4	Major Influencers of EMT
5	miRNAs

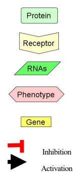

Zone	Regulators Involved
1	Cell Surface Receptors

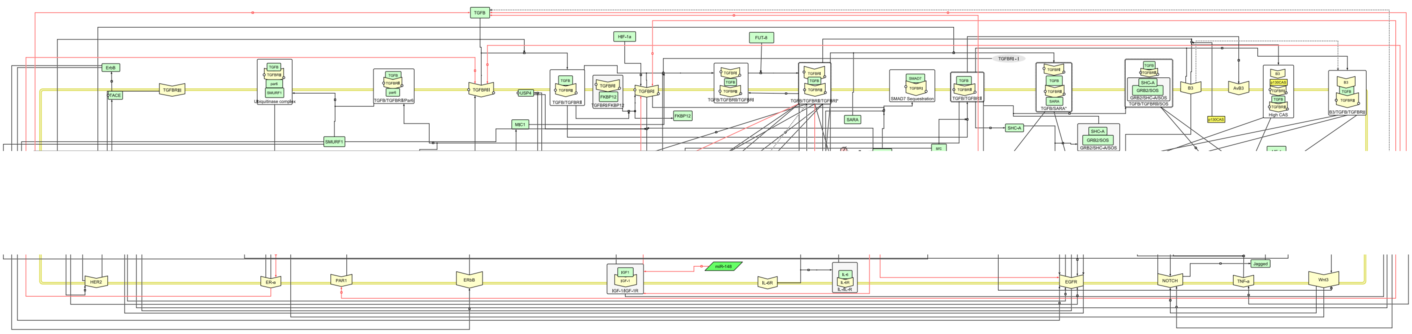

Supplementary Figure 2. Sums up all the receptors activated by TGF-β and those involved in regulating TGF-β (cross-regulation) towards modulating TGFβ induced EMT in MBC

Zone	Regulators Involved
2	SMAD Independent Pathways

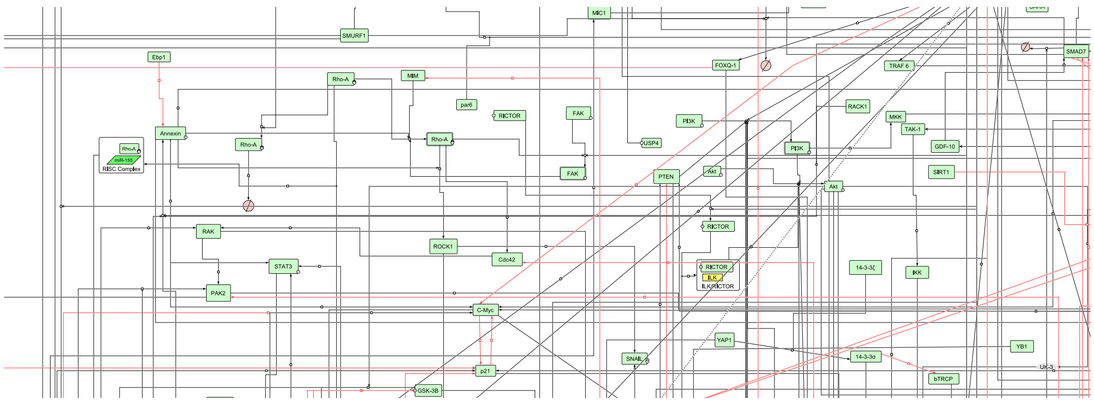

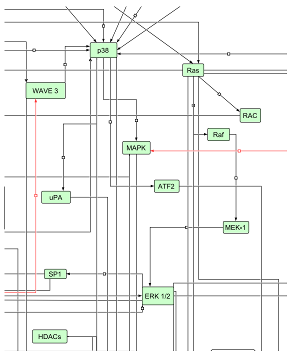

Supplementary Figure 3. Captures the SMAD independent signaling pathways Such as c-MYC, PI3K, P38, Rho, FAK involved in TGFβ induced EMT

Zone	Regulators Involved
3	SMAD Dependent Pathways

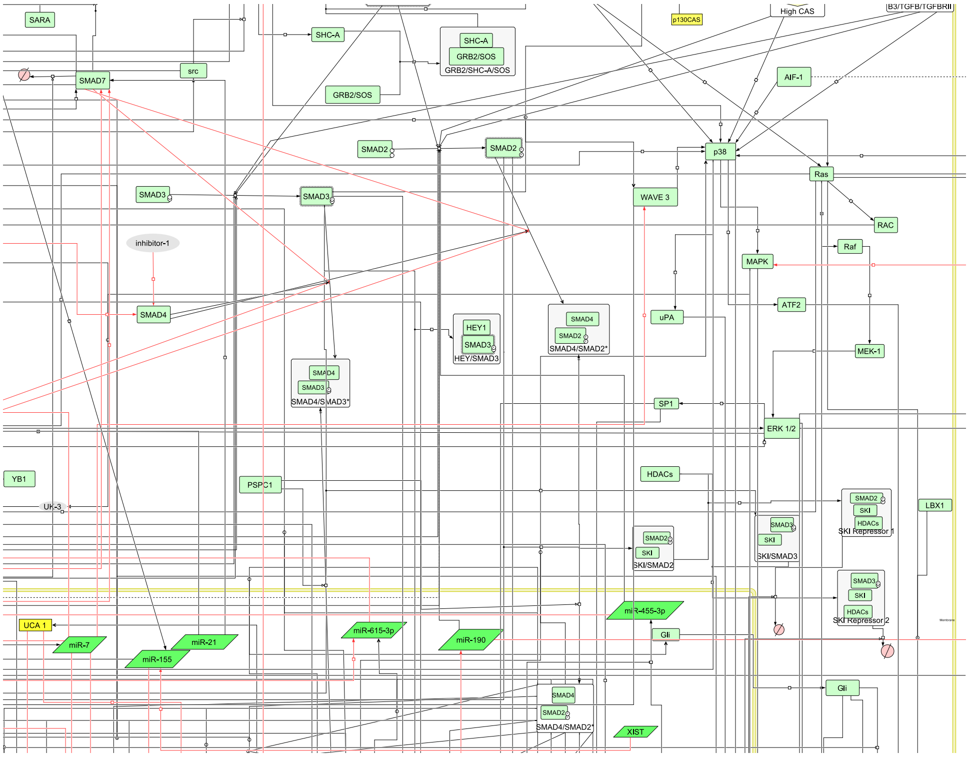

Supplementary Figure 4. Portrays the mechanism of SMAD dependent signaling pathway within TGFβ induced EMT

Zone	Regulators Involved
4	Major Influencers of EMT

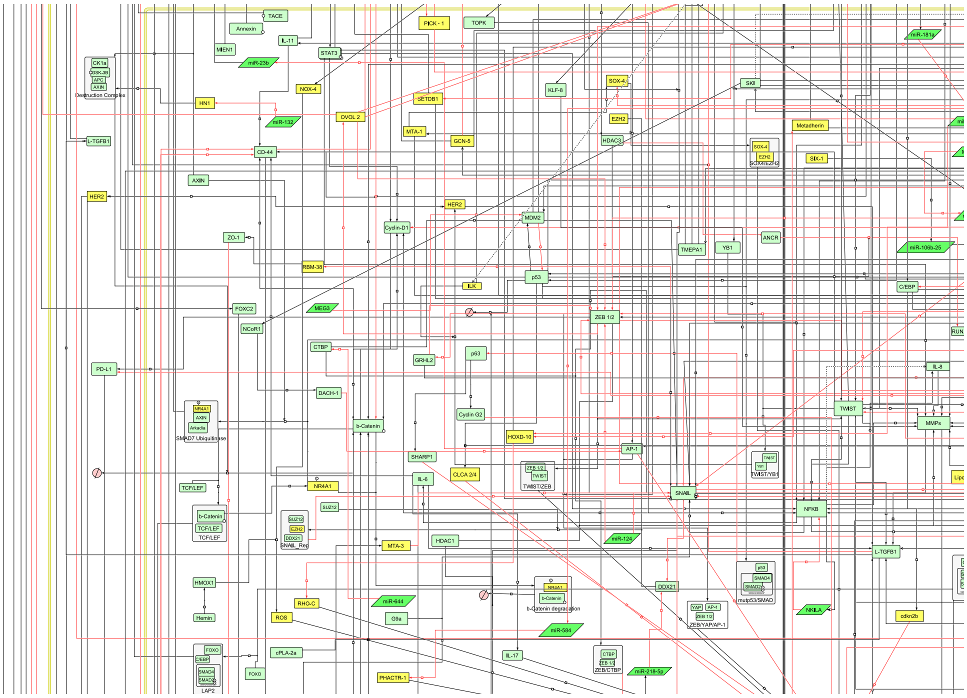

Supplementary Figure 5. Depicts the activation of major EMT regulators that subsequently guide the process of TGFβ induced EMT in MBC

Zone	Regulators Involved
5	miRNAs

Supplementary Figure 6. Portrays the activation of RNAs (miRNAs, LncRNAs) that are further involved in the process of TGFβ induced EMT in MBC

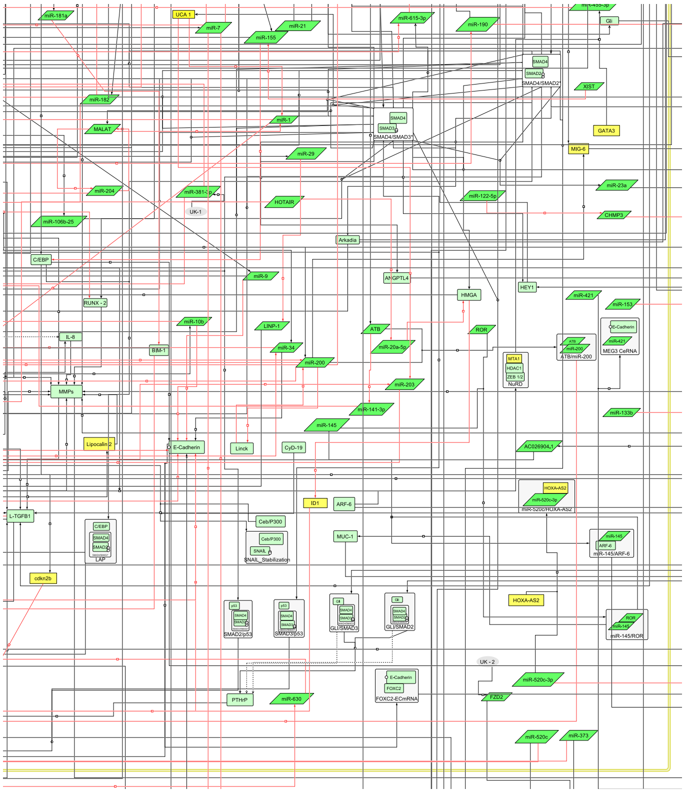

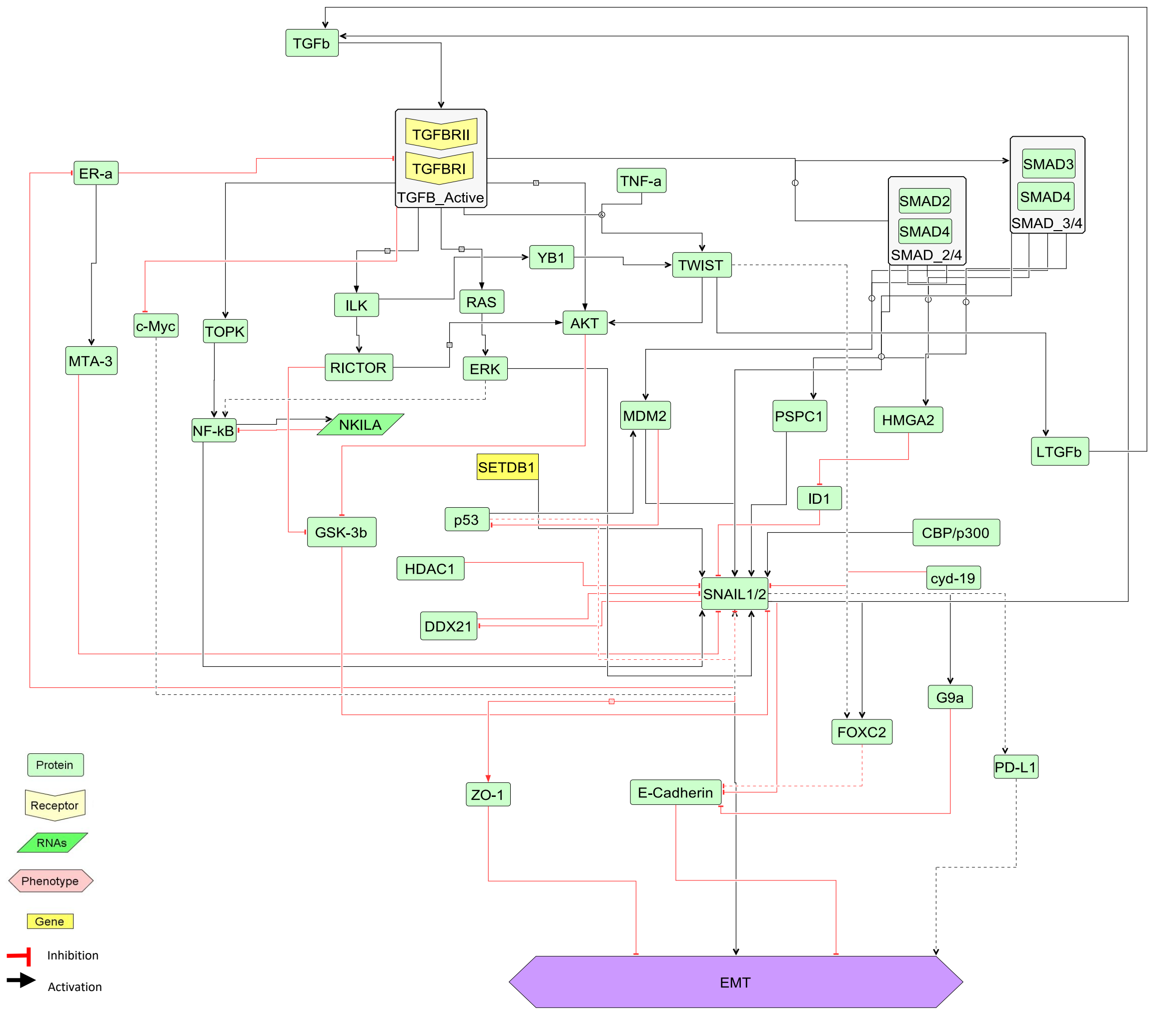

Supplementary Figure 7. TGF**β** by binding to its receptors can trigger the activation of SMAD dependent and SMAD independent signaling which in turn leading to the activation of EMT related gene SNAIL1/2 promoting tumor metastasis. Black arrows, red arrows indicate the activation, inhibition respectively. TGF**β** dependent activation of SNAIL 1/2 which in turn regulates genes like E-Cadherin, ZO-1 and p53 in inducing EMT.

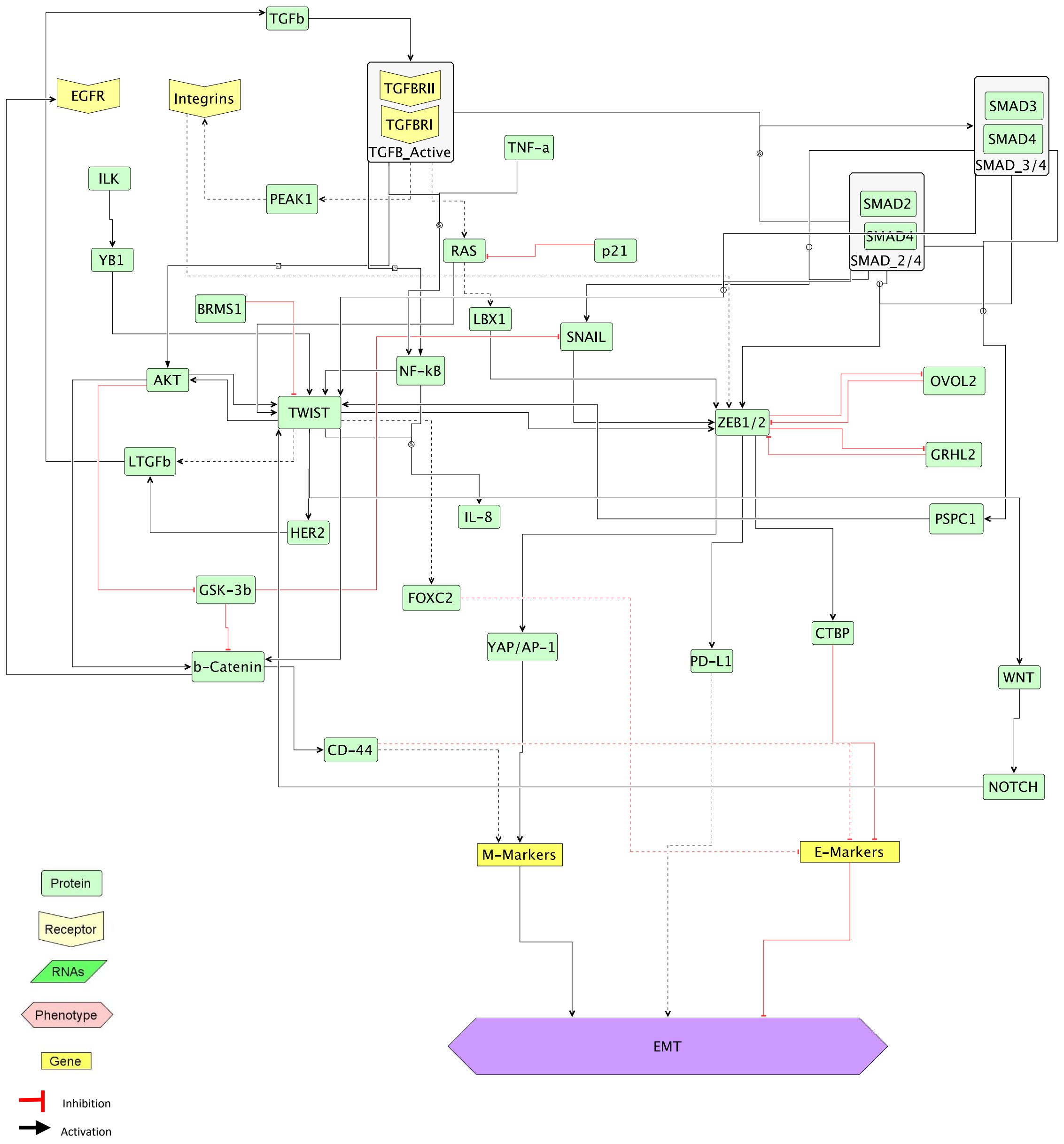

Supplementary Figure 8. TGF**β** by binding to its receptors can trigger EMT related genes TWIST1/2 and ZEB1/2. Black arrows, red arrows indicate the activation, inhibition respectively. TGF**β** parallely regulates other signaling pathways like TNF-**α**, integrins, EGFR, in modulating the regulators associated with mesenchymal phenotype and epithelial phenotype further moderating EM transition.

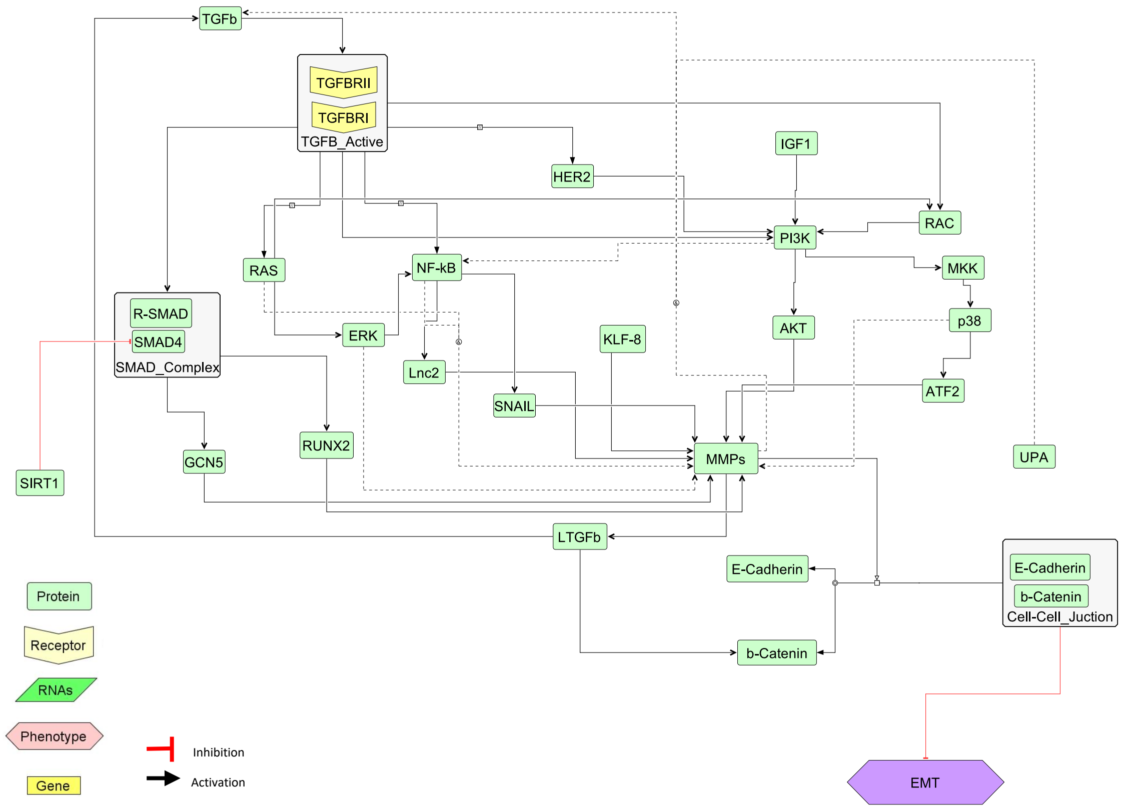

Supplementary Figure 9. TGF**β** regulates the expression of MMPs transcriptionally. This transcriptional activation occurs in a SMAD dependent or SMAD independent manner. Active MMPs further promotes Latent TGF**β** that amplifies (positive feedback) the signal and plays a crucial role in cleaving the cell-cell adhesive junctions leading to cell invasion and motility. Black arrows, red arrows indicate the activation, inhibition respectively.

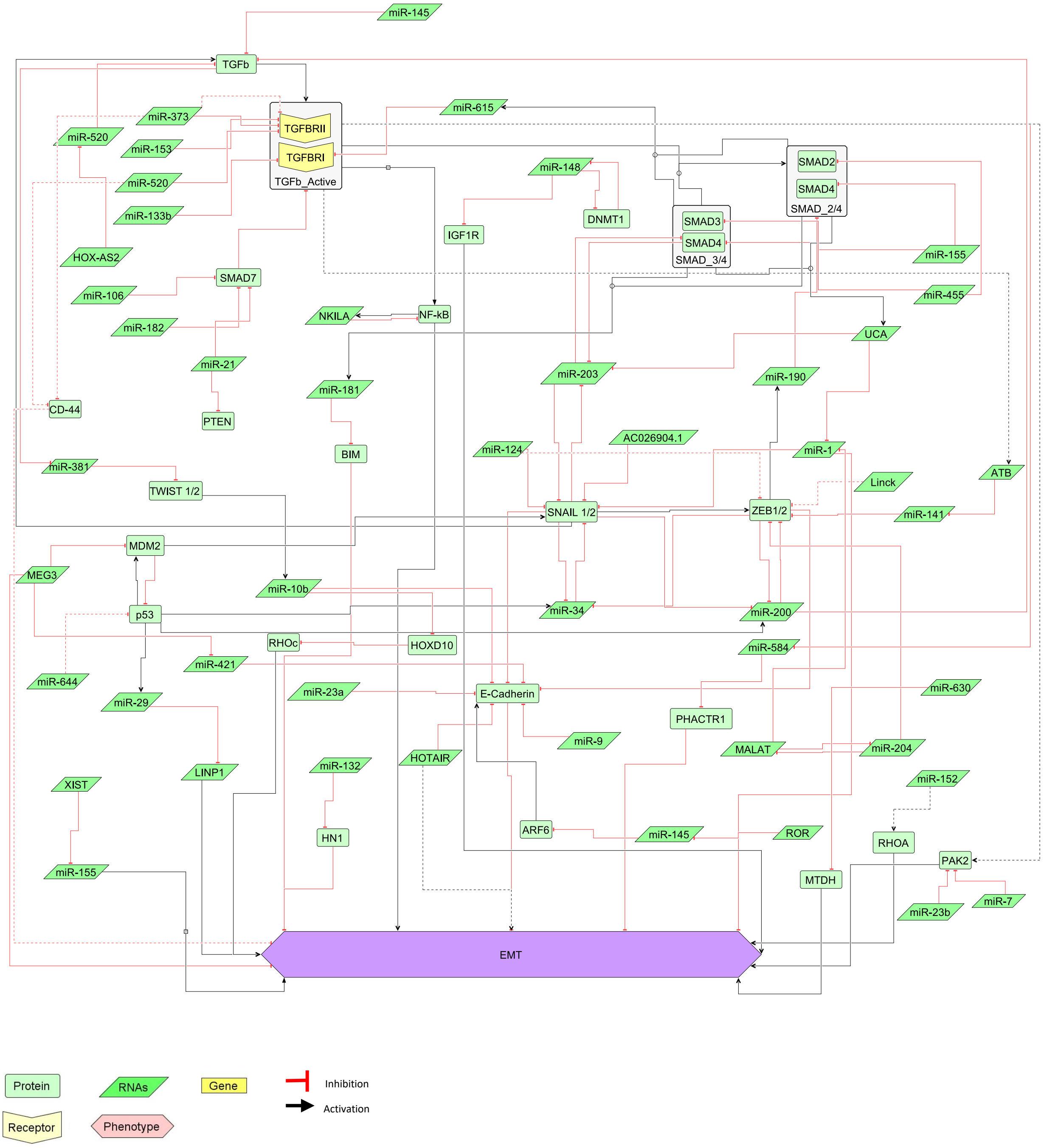

Supplementary Figure 10. TGF**β** regulates the expression of several mRNAs and miRNAs which in turn modulate TGF**β** and its receptors in modulating EM transition. While the involvement of various RNAs in EMT is understood, the precise molecular mechanisms through which TGF**β** regulates these RNAs remain an ongoing area of investigation. Black arrows, red arrows indicate the activation, inhibition respectively.

(a)

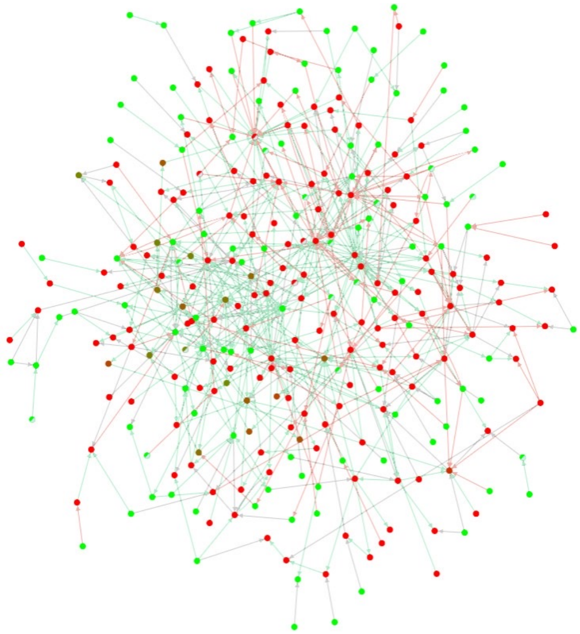

(b)

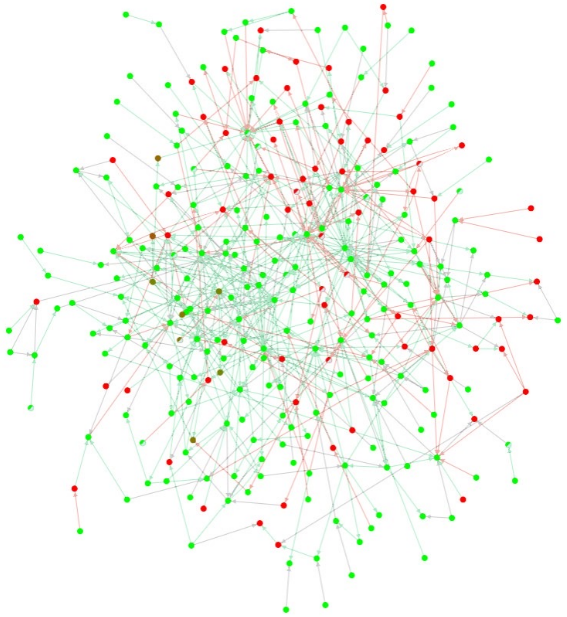

Supplementary Figure 11. Activity network of TGF**β** induced EMT in metastatic breast cancer (a) in absence of TGF**β** ,(b) in presence of TGF**β**. Green, red nodes represent the active and inactive nodes respectively. There are more active (Green) nodes observed in the presence of TGFβ. While simulating in Cell Collective the transition between E to M state can be observed clearly.

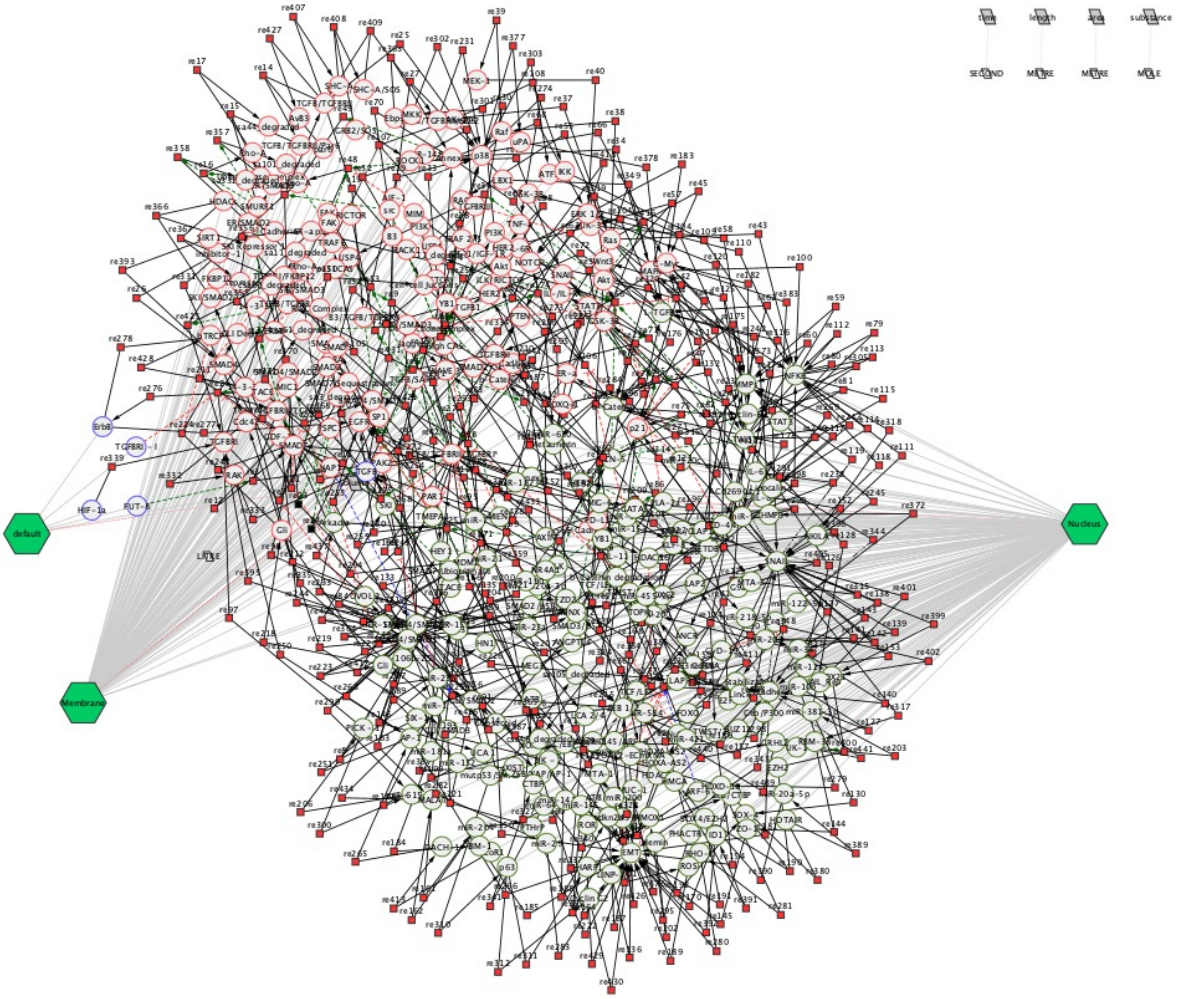

Supplementary Figure 12. The assembled network map of Metastatic Breast Cancer (MBC), imported and visualized in Cytoscape. This network comprises 746 nodes and 1344 edges, highlighting the intricate interactions and relationships within the MBC. State Transitions were observed in red squares which are the reasons for increased nodes compared to the original map shown in the article Figure 1.

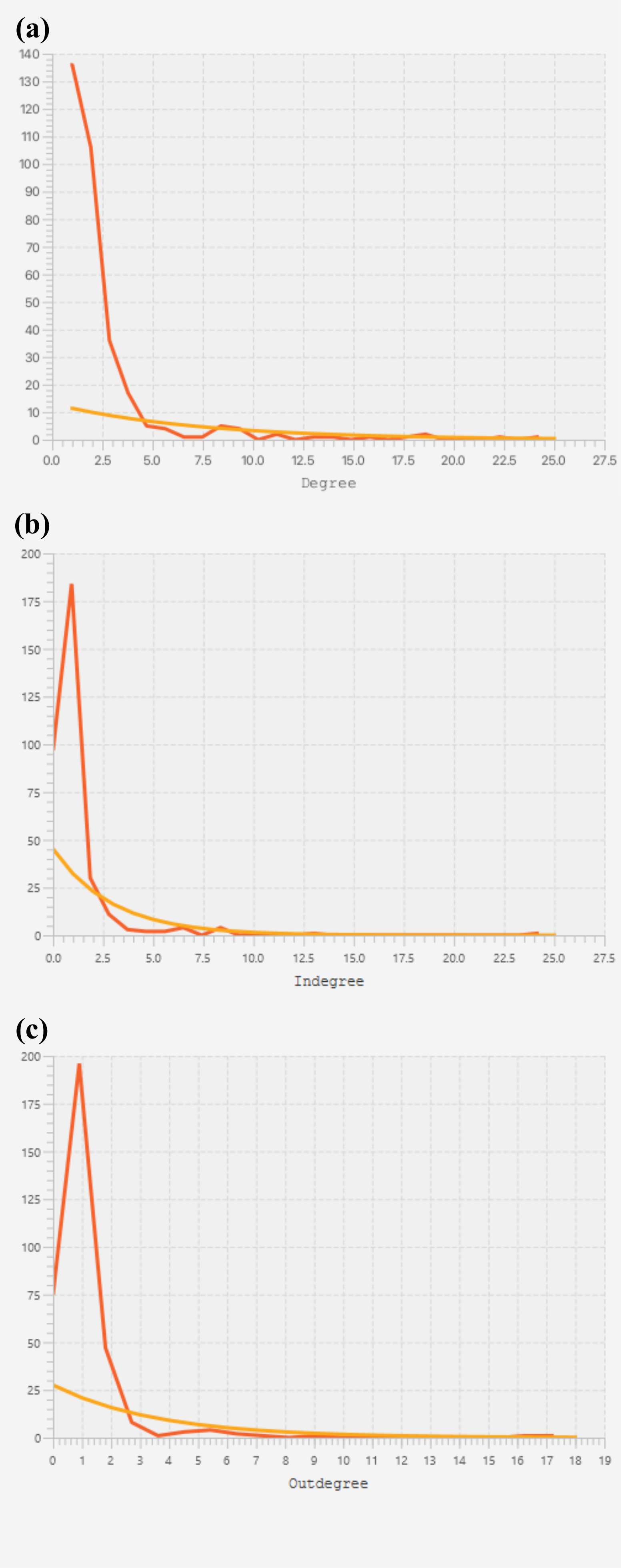

Supplementary Figure 13. Node Degree Distribution analyzed for the assembled MBC network using network analyzer plugin of Cytoscape (a) Overall degree Distribution, (b) In degree Distribution, (c) Out-Degree Distribution.

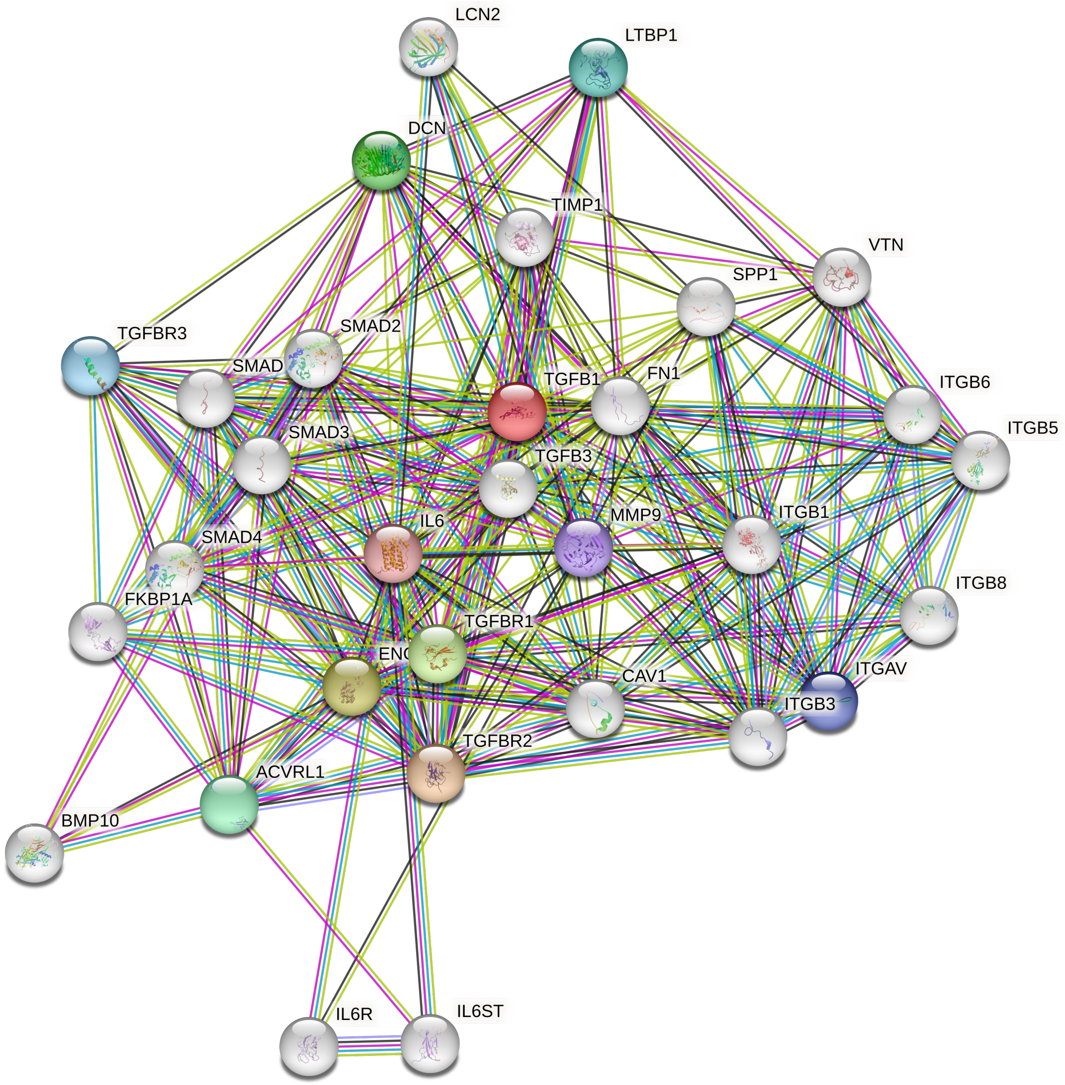

Supplementary Figure 14. TGF**β** signal transduction attained from STRING database for the automated search query of protein by name TGF**β**1 in homosapiens. Nodes represent proteins and edges represent protein-protein associations from both curated and experimentally determined databases. STRING retrieves all the associations of search query with other proteins and their associations. However, the mechanism of interactions are not provided by STRING or any similar database.

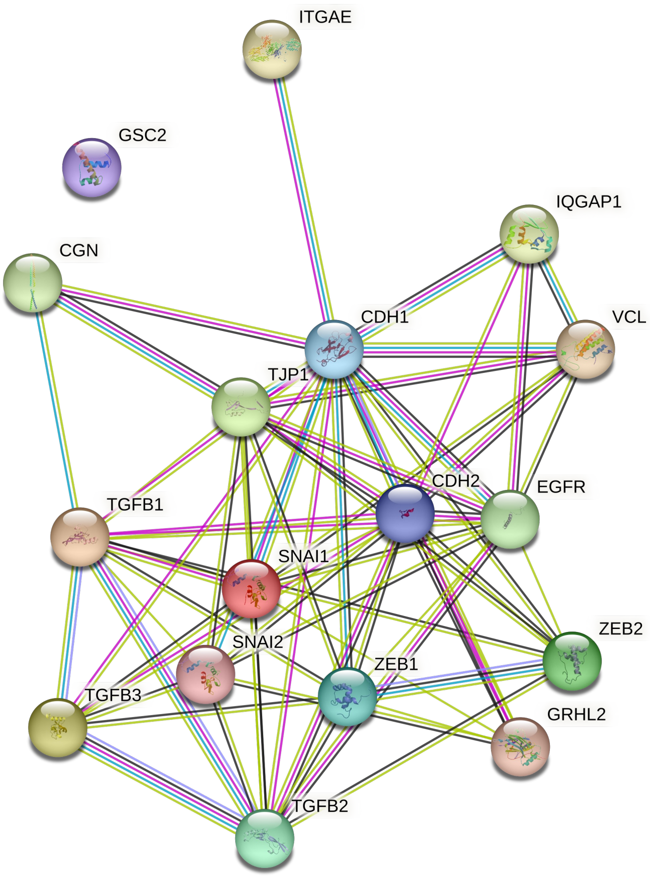

Supplementary Figure 15. TGF**β** signal transduction attained from STRING database for the automated search query of multiple proteins (TGF**β**1, TGF**β**2, TGF**β**3, SNAIL, SLUG, ZEB1, ZEB2, CDH1, ZO-1, Goosecoid, GRHL2) in homosapiens. Nodes represent proteins and edges represent protein-protein associations from both curated and experimentally determined databases. STRING retrieves the information only between the search query and their associated interactions. However, the mechanism of interactions are not provided by STRING or any similar database.

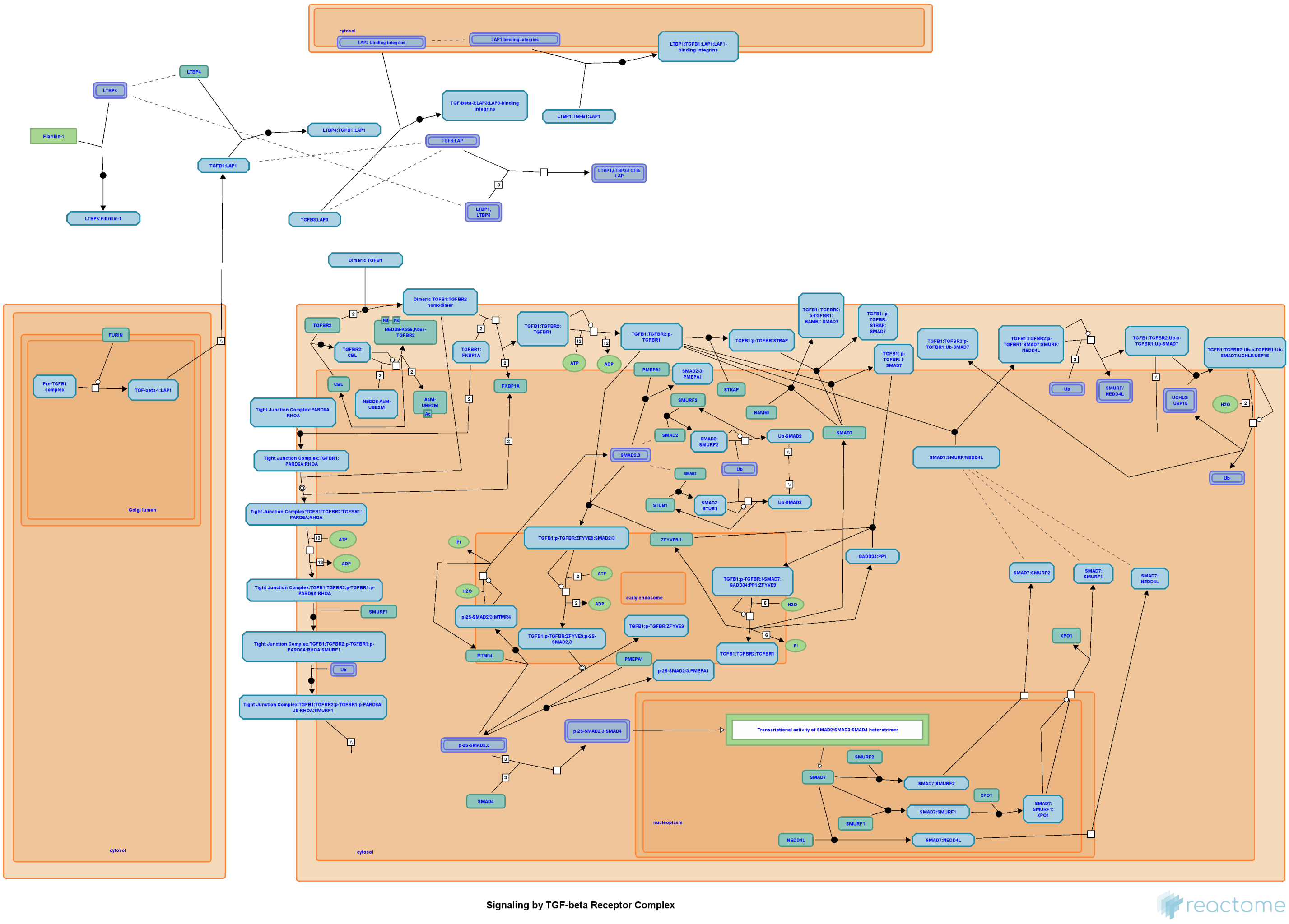

Supplementary Figure 16. The TGFβ induced EMT signal transduction network acquired from Reactome database for the search query ‘Signaling by TGFB family members’. The network contains 107 molecules and is curated from homosapiens. However, it is incomplete.

Overall Survival Analysis for the Hub Genes

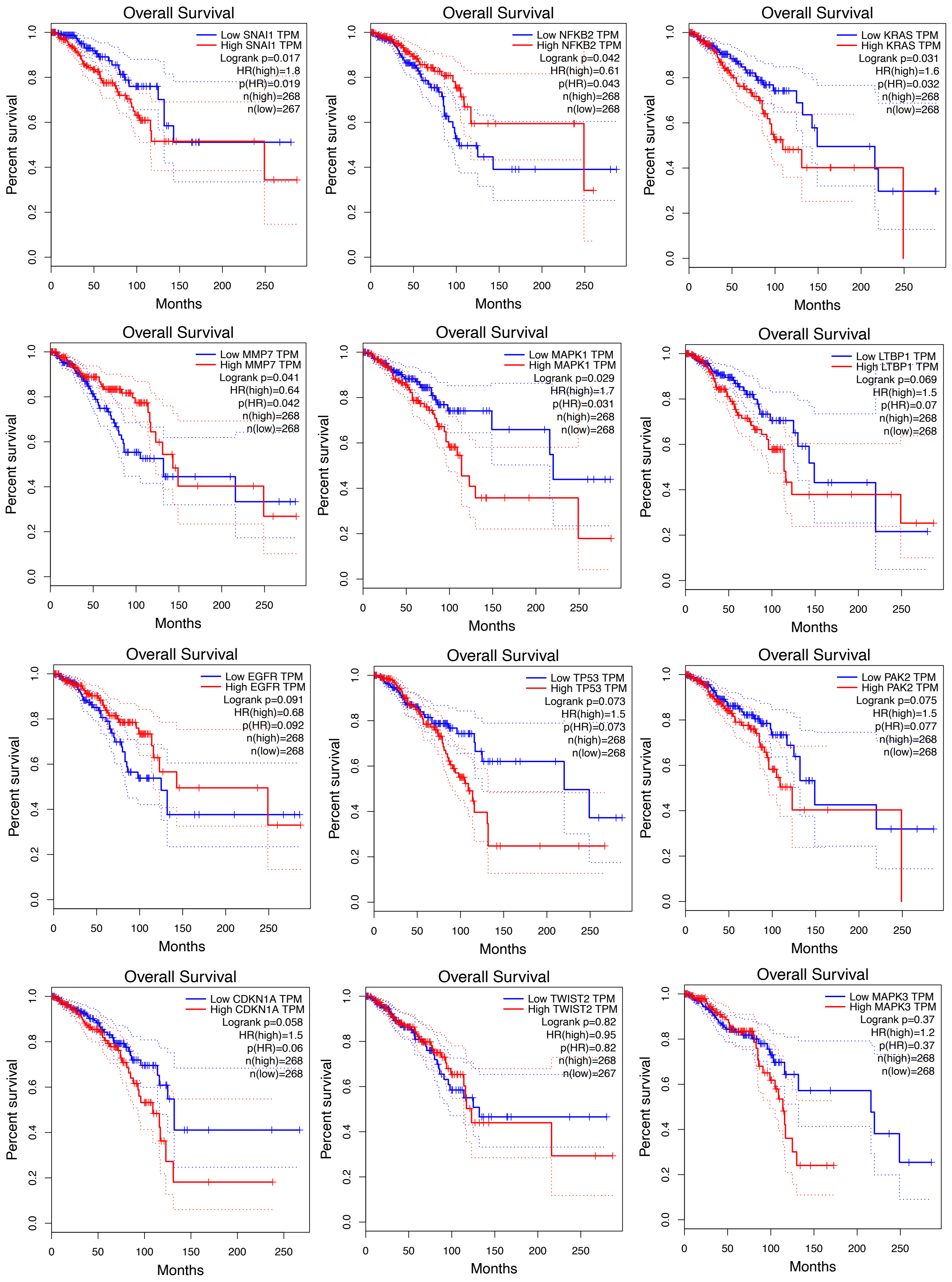

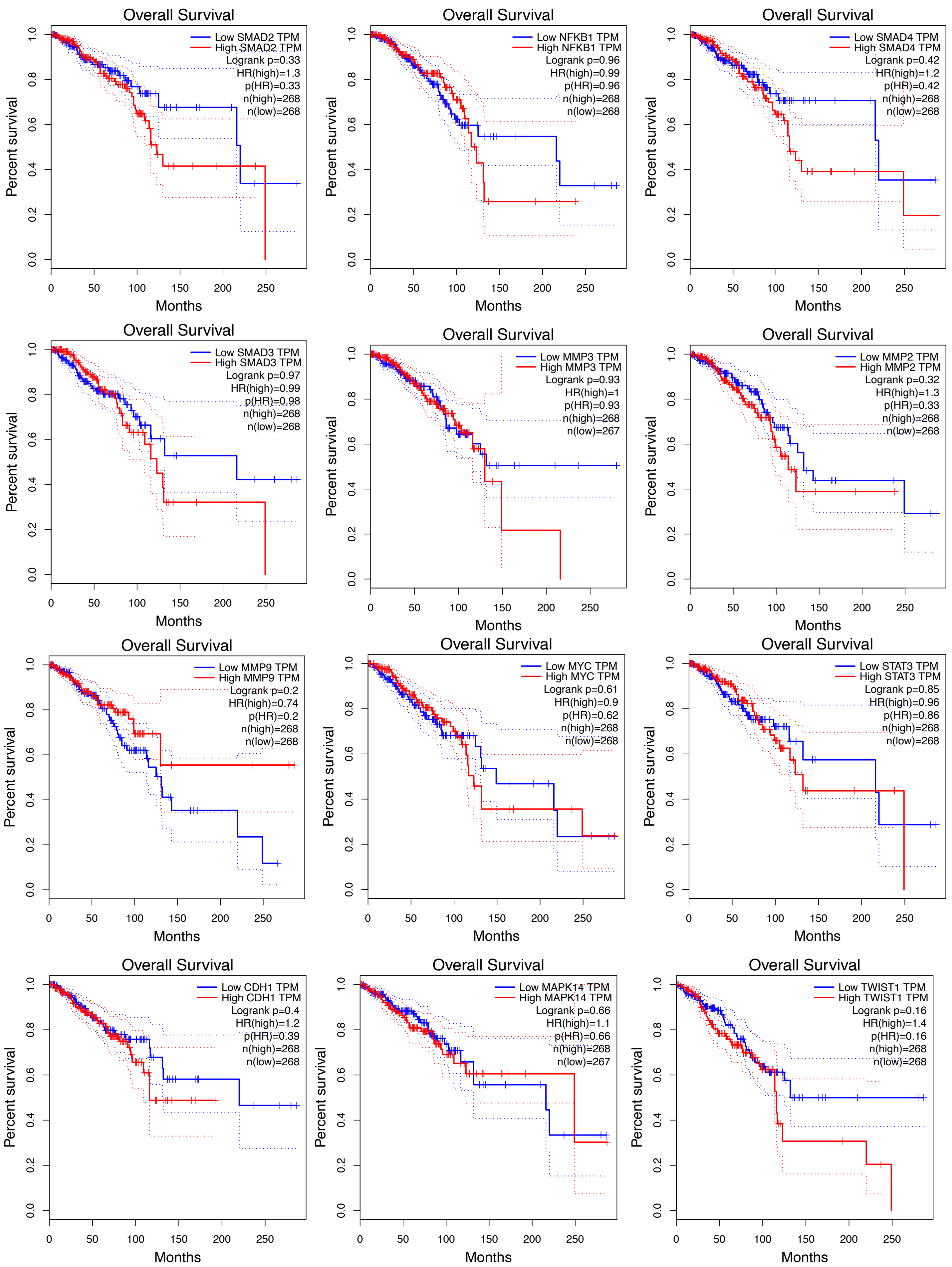
Overall Survival
Low SMAD2 TPM
High SMAD2 TPM
Logrank p=0.33
HR(high)=1.3
p(HR)=0.33
n(high)=268
n(low)=268
Overall Survival
Low NFKB1 TPM
High NFKB1 TPM
Logrank p=0.96
HR(high)=0.99
p(HR)=0.96
n(high)=268
n(low)=268
Overall Survival
Low SMAD4 TPM
High SMAD4 TPM
Logrank p=0.42
HR(high)=1.2
p(HR)=0.42
n(high)=268
n(low)=268
Overall Survival
Low SMAD3 TPM
High SMAD3 TPM
Logrank p=0.97
HR(high)=0.99
p(HR)=0.98
n(high)=268
n(low)=268
Overall Survival
Low MMP3 TPM
High MMP3 TPM
Logrank p=0.93
HR(high)=1
p(HR)=0.93
n(high)=268
n(low)=267
Overall Survival
Low MMP2 TPM
High MMP2 TPM
Logrank p=0.32
HR(high)=1.3
p(HR)=0.33
n(high)=268
n(low)=268
Overall Survival
Low MMP9 TPM
High MMP9 TPM
Logrank p=0.2
HR(high)=0.74
p(HR)=0.2
n(high)=268
n(low)=268
Overall Survival
Low MYC TPM
High MYC TPM
Logrank p=0.61
HR(high)=0.9
p(HR)=0.62
n(high)=268
n(low)=268
Overall Survival
Low STAT3 TPM
High STAT3 TPM
Logrank p=0.85
HR(high)=0.96
p(HR)=0.86
n(high)=268
n(low)=268
Overall Survival
Low CDH1 TPM
High CDH1 TPM
Logrank p=0.4
HR(high)=1.2
p(HR)=0.39
n(high)=268
n(low)=268
Overall Survival
Low MAPK14 TPM
High MAPK14 TPM
Logrank p=0.66
HR(high)=1.1
p(HR)=0.66
n(high)=268
n(low)=267
Overall Survival
Low TWIST1 TPM
High TWIST1 TPM
Logrank p=0.16
HR(high)=1.4
p(HR)=0.16
n(high)=268
n(low)=268
Percent survival
Months

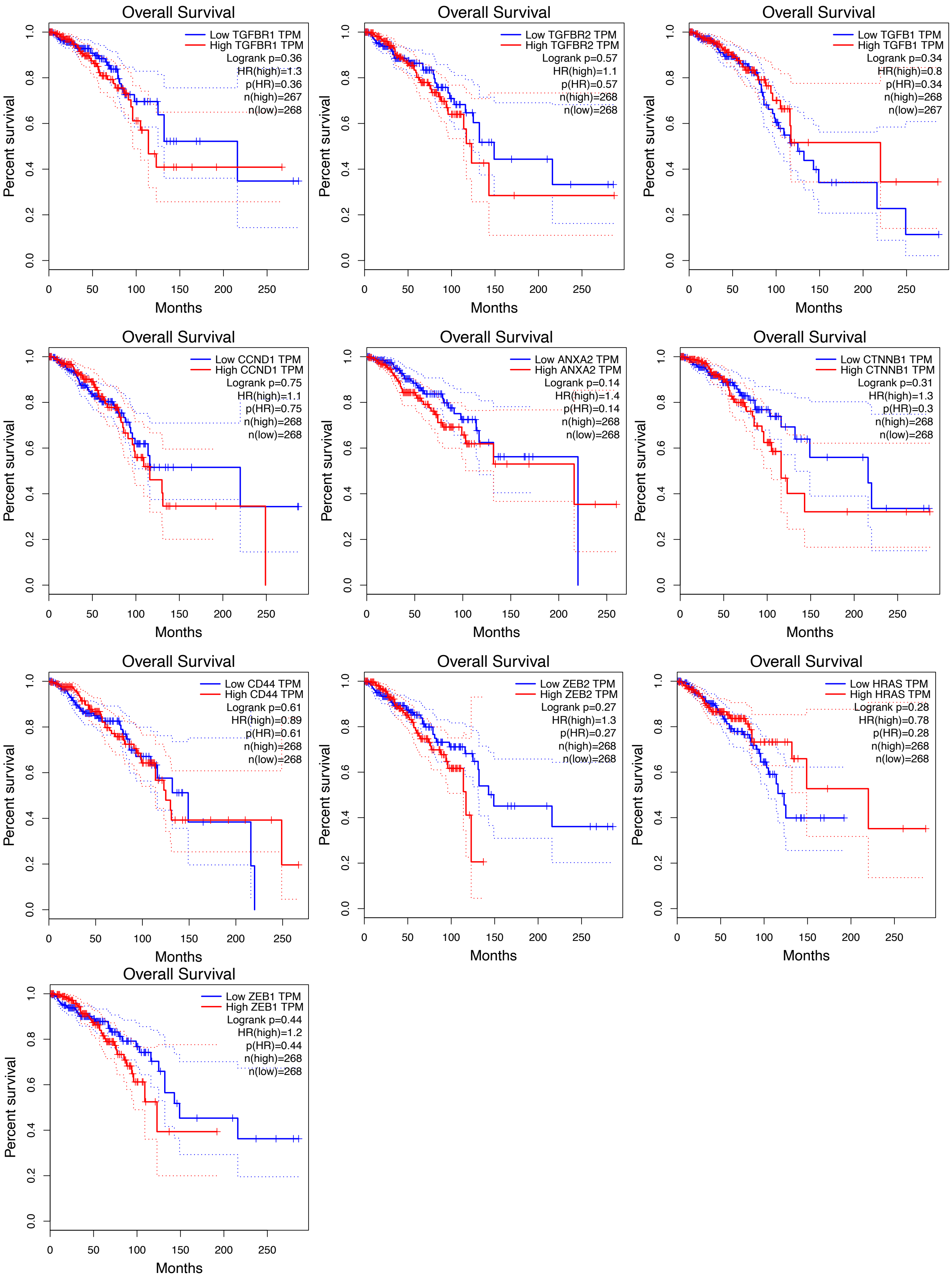

Supplementary Figure 17. Transcriptome analysis of the hub genes. Expression levels of the hub genes and their correlation with overall patient survival in invasive breast carcinoma (BRCA) obtained from the GEPIA database. Genes with log p-value of less than 0.05 were considered to be prognostically significant genes.

Disease free Survival Analysis for the hub genes

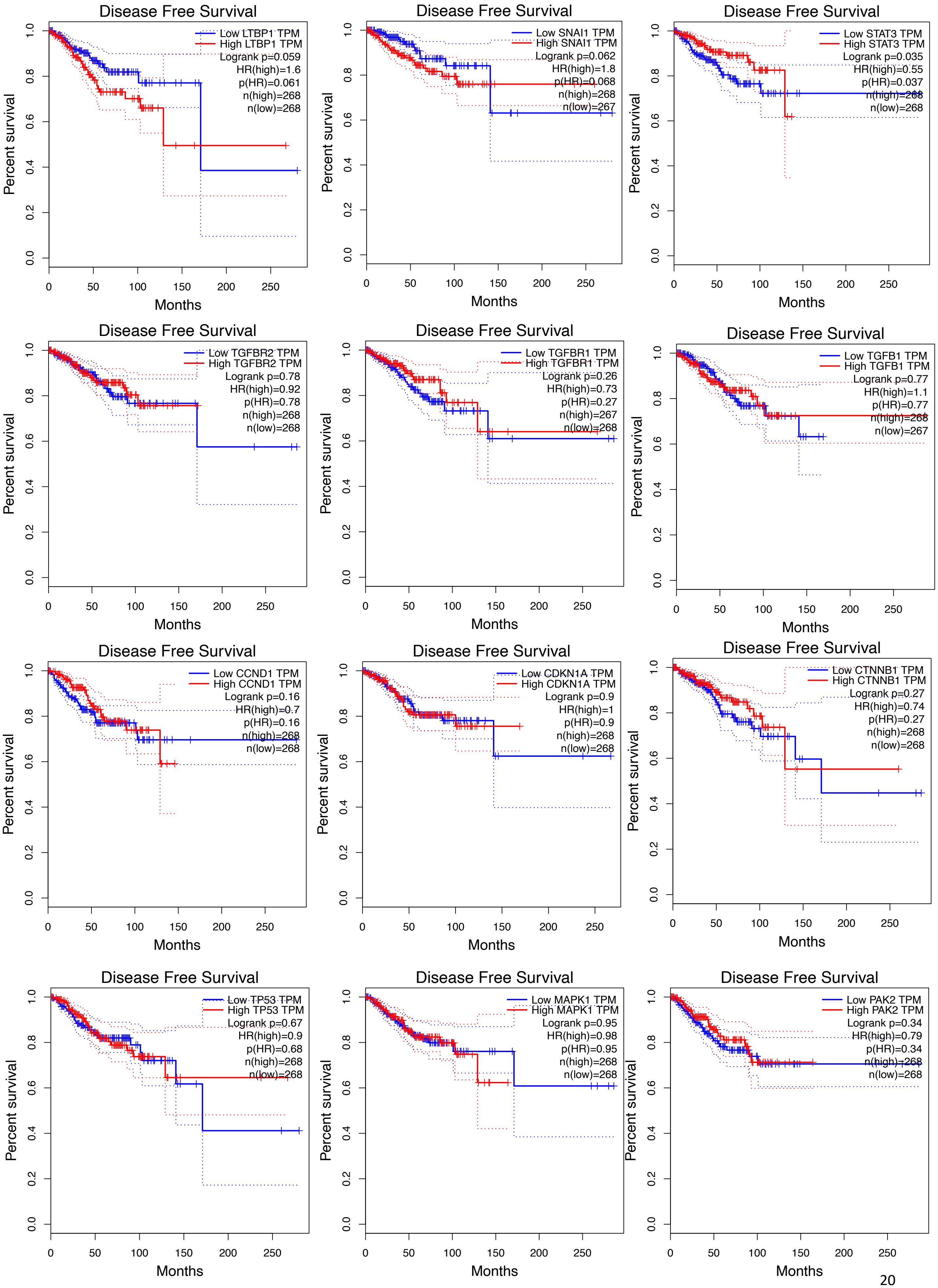

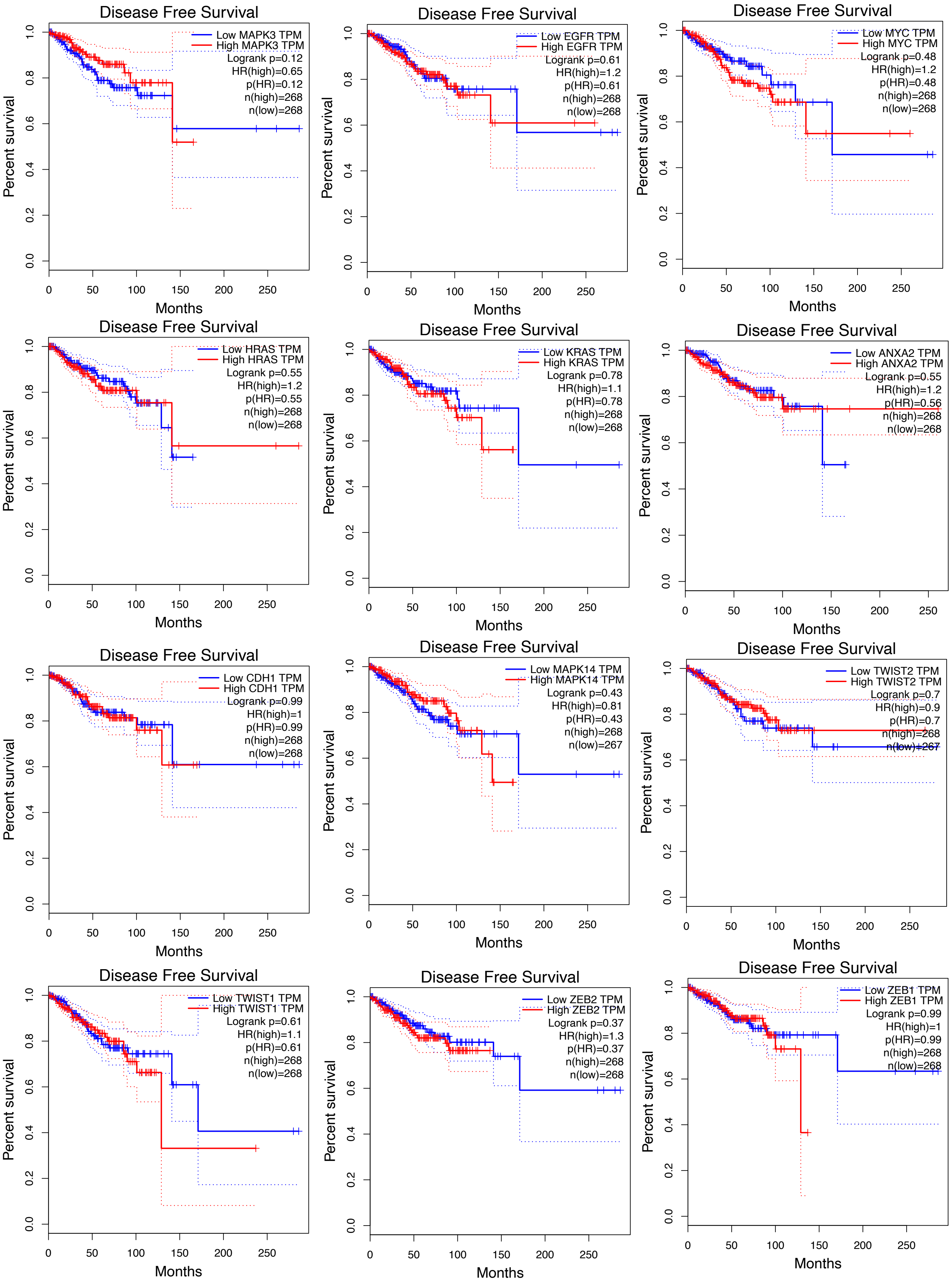
Disease Free Survival
Low MAPK3 TPM
High MAPK3 TPM
Logrank p=0.12
HR(high)=0.65
p(HR)=0.12
n(high)=268
n(low)=268
Disease Free Survival
Low EGFR TPM
High EGFR TPM
Logrank p=0.61
HR(high)=1.2
p(HR)=0.61
n(high)=268
n(low)=268
Disease Free Survival
Low MYC TPM
High MYC TPM
Logrank p=0.48
HR(high)=1.2
p(HR)=0.48
n(high)=268
n(low)=268
Disease Free Survival
Low HRAS TPM
High HRAS TPM
Logrank p=0.55
HR(high)=1.2
p(HR)=0.55
n(high)=268
n(low)=268
Disease Free Survival
Low KRAS TPM
High KRAS TPM
Logrank p=0.78
HR(high)=1.1
p(HR)=0.78
n(high)=268
n(low)=268
Disease Free Survival
Low ANXA2 TPM
High ANXA2 TPM
Logrank p=0.55
HR(high)=1.2
p(HR)=0.56
n(high)=268
n(low)=268
Disease Free Survival
Low CDH1 TPM
High CDH1 TPM
Logrank p=0.99
HR(high)=1
p(HR)=0.99
n(high)=268
n(low)=268
Disease Free Survival
Low MAPK14 TPM
High MAPK14 TPM
Logrank p=0.43
HR(high)=0.81
p(HR)=0.43
n(high)=268
n(low)=267
Disease Free Survival
Low TWIST2 TPM
High TWIST2 TPM
Logrank p=0.7
HR(high)=0.9
p(HR)=0.7
n(high)=268
n(low)=267
Disease Free Survival
Low TWIST1 TPM
High TWIST1 TPM
Logrank p=0.61
HR(high)=1.1
p(HR)=0.61
n(high)=268
n(low)=268
Disease Free Survival
Low ZEB2 TPM
High ZEB2 TPM
Logrank p=0.37
HR(high)=1.3
p(HR)=0.37
n(high)=268
n(low)=268
Disease Free Survival
Low ZEB1 TPM
High ZEB1 TPM
Logrank p=0.99
HR(high)=1
p(HR)=0.99
n(high)=268
n(low)=268
Percent survival
Months

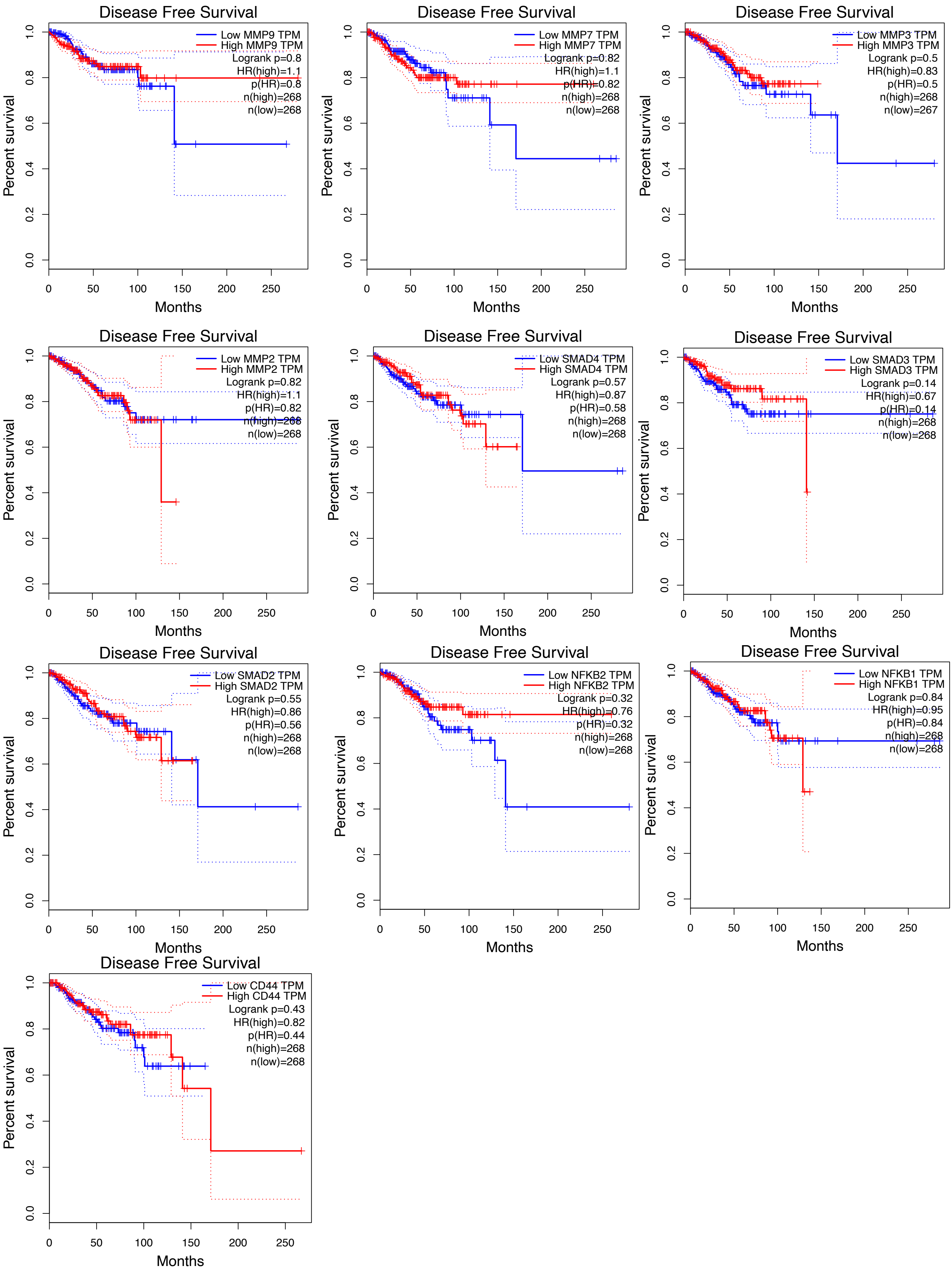

Supplementary Figure 18. Transcriptome analysis of the hub genes. Expression levels of the hub genes and their correlation with disease-free patient survival in invasive breast carcinoma (BRCA) obtained from GEPIA database. Genes with log p-value of less than 0.05 were considered to be prognostically significant genes.

Supplementary Tables

Supplementary Table 1. Downstream mediators of TGFβ signaling involved and their corresponding HGNC nomenclature in Metastatic Breast Cancer.

Given Symbol	Approved Symbol	Name	ID	Reference
MDM2	MDM2	MDM2 proto-oncogene	HGNC:6973	1-3
MEK-1	MAP2K1	mitogen-activated protein kinase 1	HGNC:6840	4,5
MIC1	GDF15	growth differentiation factor 15	HGNC:30142	6-8
MIEN1	MIEN1	migration and invasion enhancer 1	HGNC:28230	9
MIG-6	ERRFI1	ERBB receptor feedback inhibitor 1	HGNC:18185	10
MIM	MTSS1	MTSS I-BAR domain containing 1	HGNC:20443	11
MKK	MAP2K3	mitogen-activated protein kinase 3	HGNC:6843	4,5
	MAP2K4	mitogen-activated protein kinase 4	HGNC:6844	
MMP 9	MMP9	Matrix Metallopeptidase9	HGNC:7176	4,5,12-22
MMP 7	MMP7	Matrix Metallopeptidase7	HGNC:7174	
MMP 3	MMP3	Matrix Metallopeptidase3	HGNC:7173	
MMP 2	MMP2	Matrix Metallopeptidase2	HGNC:7166	
MTA-3	MTA3	metastasis associated 1 family member 3	HGNC:23784	23
MUC-1	MUC1	mucin 1, cell surface associated	HGNC:7508	24
Metadherin	MTDH	metadherin	HGNC:29608	25
NCoR1	NCOR1	nuclear receptor corepressor 1	HGNC:7672	26
NFKB	NFKB1	nuclear factor kappa B subunit 1	HGNC:7794	13,14,19,27-32
	NFKB2	nuclear factor kappa B subunit 2	HGNC:7795	
NOTCH	NOTCH 1	notch receptor 1	HGNC:7881	33-36
	NOTCH 2	notch receptor 2	HGNC:7882	

	NOTCH 3	notch receptor 3	HGNC:7883	
	NOTCH 4	notch receptor 4	HGNC:7884	
NOX-4	NOX4	NADPH oxidase 4	HGNC:7891	37,38
NR4A1	NR4A1	nuclear receptor subfamily 4 group A member 1	HGNC:7980	39
OVOL 2	OVOL2	ovo like zinc finger 2	HGNC:15804	40
	OVOL1	ovo like transcriptional repressor 1	HGNC:8525	
PAK2	PAK2	p21 (RAC1) activated kinase 2	HGNC:8591	41-43
PAR1	F2R	coagulation factor II thrombin receptor	HGNC:3537	44
PD-L1	CD274	CD274 molecule	HGNC:17635	45
PHACTR-1	PHACTR1	phosphatase and actin regulator 1	HGNC:20990	46
PI3K	PIK3CA	phosphatidylinositol-4,5-bisphosphate 3-kinase catalytic subunit alpha	HGNC:8975	4,5,12,27,47-50
PICK - 1	PICK1	protein interacting with PRKCA 1	HGNC:9394	51,52
PSPC1	PSPC1	paraspeckle component 1	HGNC:20320	53
PTEN	PTEN	phosphatase and tensin homolog	HGNC:9588	27,54,55
PTHrP	PTHLH	parathyroid hormone like hormone	HGNC:9607	56
RAC	RAC1	Rac family small GTPase 1	HGNC:9801	4,43
RACK1	RACK1	receptor for activated C kinase 1	HGNC:4399	57,58
RAK	FRK	fyn related Src family tyrosine kinase	HGNC:3955	41
RBM-38	RBM38	RNA binding motif protein 38	HGNC:15818	59
RHO-C	RHOC	ras homolog family member C	HGNC:669	60
ROCK1	ROCK1	Rho associated coiled-coil containing protein kinase 1	HGNC:10251	61
ROS	ROS1	ROS proto-oncogene 1, receptor tyrosine kinase	HGNC:10261	62

RUNX - 2	RUNX2	RUNX family transcription factor 2	HGNC:10472	18,63
Raf	RAF1	Raf-1 proto-oncogene, serine/threonine kinase	HGNC:9829	4,5
Ras	KRAS	KRAS proto-oncogene, GTPase	HGNC:6407	19,64-66
	HRAS	HRas proto-oncogene, GTPase	HGNC:5173	
Rho-A	RHOA	ras homolog family member A	HGNC:667	11,43,61,67
SARA	ZFYVE9	zinc finger FYVE-type containing 9	HGNC:6775	68-70
SETDB1	SETDB1	SET domain bifurcated histone lysine methyltransferase 1	HGNC:10761	71,72
SHARP1	BHLHE41	basic helix-loop-helix family member e41	HGNC:16617	65
SIRT1	SIRT1	sirtuin 1	HGNC:14929	21,73
SIX-1	SIX1	SIX homeobox 1	HGNC:10887	74
SKI	SKI	SKI proto-oncogene	HGNC:10896	75-77
SMAD2	SMAD2	SMAD family member 2	HGNC:6768	78-80
SMAD3	SMAD3	SMAD family member 3	HGNC:6769	
SMAD4	SMAD4	SMAD family member 4	HGNC:6770	
SMAD7	SMAD7	SMAD family member 7	HGNC:6773	81,82
SMURF1	SMURF1	SMAD specific E3 ubiquitin protein ligase 1	HGNC:16807	83,84
SNAIL	SNAI1	snail family transcriptional repressor 1	HGNC:11128	1,23,30,31,45,53,59,85-90
	SNAI2	snail family transcriptional repressor 2	HGNC:11094	
	SNAI3	snail family transcriptional repressor 3	HGNC:18411	
SOX-4	SOX4	SRY-box transcription factor 4	HGNC:11200	91-93
SP1	SP1	Sp1 transcription factor	HGNC:11205	94
STAT3	STAT3	signal transducer and activator of transcription 3	HGNC:11364	95-97

TACE	ADAM17	ADAM metallopeptidase domain 17	HGNC:195	50
TAK-1	MAP3K7	mitogen-activated protein kinase kinase kinase 7	HGNC:6859	28,29
TCF				39
TGFB	TGFB1	transforming growth factor beta 1	HGNC:11766	70,98
	TGFB2	transforming growth factor beta 2	HNGC:11768	
	TGFB3	transforming growth factor beta 3	HNGC:11769	
LEF	LEF1	lymphoid enhancer binding factor 1	HGNC:6551	12,39
par6	PAR-6	par-6 family cell polarity regulator alpha	HGNC:15943	99
TGFBRI<br>TGFBRI - I	TGFBR1	transforming growth factor beta receptor 1	HGNC:11772	100
				101
TGFBRII	TGFBR2	transforming growth factor beta receptor 2	HGNC:11773	29,102-104
TGFBRIII	TGFBR3	transforming growth factor beta receptor 3	HGNC:11774	105
TMEPA1	PMEPA1	prostate transmembrane protein, androgen induced 1	HGNC:14107	68,69
TNF-a	TNF	tumor necrosis factor	HGNC:11892	28,106
TOPK	PBK	PDZ binding kinase	HGNC:18282	31
TRAF 2	TRAF2	TNF receptor associated factor 2	HGNC:12032	28,29
TRAF 6	TRAF6	TNF receptor associated factor 6	HGNC:12036	
TWIST	TWIST1	twist family bHLH transcription factor 1	HGNC:12428	36,106-108
	TWIST2	twist family bHLH transcription factor 2	HGNC:20670	
UCA 1	UCA1	urothelial cancer associated 1	HGNC:37126	109
TRAF 5	TRAF5	TNF receptor associated factor 5	HGNC:12035	28,29
USP	USP4	ubiquitin specific peptidase 4	HGNC:12627	83

	USP11	ubiquitin specific peptidase 11	HGNC:12609	
	USP15	ubiquitin specific peptidase 15	HGNC:12613	
	USP19	ubiquitin specific peptidase 19	HGNC:12617	
WAVE 3	WASF3	WASP family member 3	HGNC:12734	110,111
WNT				36
YAP1	YAP1	Yes1 associated transcriptional regulator	HGNC:16262	112
YB1	YBX1	Y-box binding protein 1	HGNC:8014	113
CTBP	CTBP1	C-terminal binding protein 1	HGNC:2494	112,114
ZEB	ZEB1	zinc finger E-box binding homeobox 1	HGNC:11642	64,115-117
	ZEB2	zinc finger E-box binding homeobox 2	HGNC:14881	
ZO-1	TJP1	tight junction protein 1	HGNC:11827	59
b-Catenin	CTNNB1	catenin beta 1	HGNC:2514	12,36,118,119
bTRCP	BTRC	beta-transducin repeat containing E3 ubiquitin protein ligase	HGNC:1144	56
cPLA-2a	PLA2G4A	phospholipase A2 group IVA	HGNC:9035	120
cdkn2b	CDKN2B	cyclin dependent kinase inhibitor 2B	HGNC:1788	121
GRHL2	GRHL2	grainyhead like transcription factor 2	HGNC:2799	122-124
14-3-3s	YWHA group			56
14-3-3z	YWHAZ	KCIP-1,	HGNC:12855	
AIF-1	AIF1	allograft inflammatory factor 1	HGNC:352	125,126
ANGPTL4	ANGPTL4	angiopoietin like 4	HGNC:16039	127
AP-1	FOS	Fos proto-oncogene, AP-1 transcription factor subunit	HGNC:3796	128-130
	JUN	Jun proto-oncogene, AP-1 transcription factor subunit	HGNC:6204	

	JUND	JunD proto-oncogene, AP-1 transcription factor subunit	HGNC:6206	
	FOSB	FosB proto-oncogene, AP-1 transcription factor subunit	HGNC:3797	
ARF-6	ARF6	ADP ribosylation factor 6	HGNC:659	131
ATF2	ATF2	activating transcription factor 2	HGNC:784	4,5
AXIN	AXIN1	axin 1	HGNC:903	39
	AXIN2	axin2	HGNC:904	
Akt	AKT1	AKT serine/threonine kinase 1	HGNC:391	17,27,50,132,133
	AKT2	AKT serine/threonine kinase 2	HGNC:392	
	AKT3	AKT serine/threonine kinase 3	HGNC:393	
Annexin	ANXA2	annexin A2	HGNC:537	9,57,95,134
Arkadia	RNF111	ring finger protein 111	HGNC:17384	75,76
AvB3	ITGA5	integrin subunit alpha 5	HGNC:6141	104,135
B3	ITGB3	integrin subunit beta 3	HGNC:6156	
BIM-1	BCL2L11	BCL2 like 11	HGNC:994	136,137
C-Myc	MYC	MYC proto-oncogene, bHLH transcription factor	HGNC:7553	53,107,138-140
C/EBP	CEBPA	CCAAT enhancer binding protein alpha	HGNC:1833	121,140
	CEBPB	CCAAT enhancer binding protein beta	HGNC:1834	
	CEBPD	CCAAT enhancer binding protein delta	HGNC:1835	
CD-44	CD44	CD44 molecule (Indian blood group)	HGNC:1681	141-143
CLCA 2	CLCA2	chloride channel accessory 2	HGNC:2016	144
Cdc42	CDC42	cell division cycle 42	HGNC:1736	43
Ceb/P300	EP300	E1A binding protein p300	HGNC:3373	89,121,140
CyD-19				89

Cyclin G2	CCNG2	cyclin G2	HGNC:1593	65
Cyclin-D1	CCND1	cyclin D1	HGNC:1582	95,118,125,126,145
DACH-1	DACH1	dachshund family transcription factor 1	HGNC:2663	26
DDX21	DDX21	DExD-box helicase 21	HGNC:2744	146
CLCA 4	CLCA4	chloride channel accessory 4	HGNC:2018	144
E-Cadherin	CDH1	cadherin 1	HGNC:1748	73,147,148
E2F5	E2F5	E2F transcription factor 5	HGNC:3119	140
EGFR	EGFR	epidermal growth factor receptor	HGNC:3236	94,134
ER-aR	ESR1	estrogen receptor 1	HGNC:3467	84,86,87
ER-a ptn				
ERK 2	MAPK1	mitogen-activated protein kinase 1	HGNC:6871	4,28,29
ERK 1	MAPK3	mitogen-activated protein kinase 3	HGNC:6877	
ERbB	ERBB2	erb-b2 receptor tyrosine kinase 2	HGNC:3430	27,113
EZH2	EZH2	enhancer of zeste 2 polycomb repressive complex 2 subunit	HGNC:3527	146
Ebp1	PA2G4	proliferation-associated 2G4	HGNC:8550	145
FAK	PTK2	protein tyrosine kinase 2	HGNC:9611	149
FKBP12	FKBP1A	FKBP prolyl isomerase 1A	HGNC:3711	150-152
FOXC2	FOXC2	forkhead box C2	HGNC:3801	153
SOS	SOS2	SOS Ras/Rho guanine nucleotide exchange factor 2	HGNC:11188	19,135
	SOS1	SOS Ras/Rac guanine nucleotide exchange factor 1	HGNC:11187	
FOXO	FOXO1	forkhead box O1	HGNC:3819	140
	FOXO3	forkhead box O3	HGNC:3821	

	FOXO4	forkhead box O4	HGNC:7139	
FOXQ-1	FOXQ1	forkhead box Q1	HGNC:20951	148
FUT-8	FUT8	fucosyltransferase 8	HGNC:4019	103
G9a	EHMT2	euchromatic histone lysine methyltransferase 2	HGNC:14129	147
GATA3	GATA3	GATA binding protein 3	HGNC:4172	154
GCN-5	KAT2A	lysine acetyltransferase 2A	HGNC:4201	17
	KAT2B			
GDF-10	GDF10	growth differentiation factor 10	HGNC:4215	81
GRB2	GRB2	growth factor receptor bound protein 2	HGNC:4566	19,135
SHC-A	SHC1	SHC adaptor protein 1	HGNC:10840	
GSK-3B	GSK3B	glycogen synthase kinase 3 beta	HGNC:4617	132
Gli	GLI1	GLI family zinc finger 1	HGNC:4317	56
	GLI2	GLI family zinc finger 2	HGNC:4318	
	GLI3	GLI family zinc finger 3	HGNC:4319	
	GLI4	GLI family zinc finger 4	HGNC:4320	
Goosecoid	GSC	goosecoid homeobox	HGNC:4612	155
HDAC1	HDAC1	histone deacetylase 1	HGNC:4852	63,75-77,88,156
HDAC3	HDAC3	histone deacetylase 3	HGNC:4854	
HER3	ErBb3		HGNC:3431	50
HEY1	HEY1	hes related family bHLH transcription factor with YRPW motif 1	HGNC:4880	33,34
HIF-1a	HIF1A	hypoxia inducible factor 1 subunit alpha	HGNC:4910	157
HMGA	HMGA1	high mobility group AT-hook 1	HGNC:5010	90
	HMGA2	high mobility group AT-hook 2	HGNC:5009	
HMOX1	HMOX1	heme oxygenase 1	HGNC:5013	62

HN1	JPT1	Jupiter microtubule associated homolog 1	HGNC:14569	158
HOXA-AS2	HOXA-AS2	HOXA cluster antisense RNA 2	HGNC:43745	159
HOXD-10	HOXD10	homeobox D10	HGNC:5133	60
Hemin	EIF2AK1	eukaryotic translation initiation factor 2 alpha kinase 1	HGNC:24921	62
ID1	ID1	inhibitor of DNA binding 1	HGNC:5360	90
IGF-1	IGF1	insulin like growth factor 1	HGNC:5464	12
IKK	CHUK	component of inhibitor of nuclear factor kappa B kinase complex	HGNC:1974	28,29,32
IGF-1R	IGF1R	insulin like growth factor 1 receptor	HGNC:5465	12
IL-11	IL11	interleukin 11	HGNC:5966	128-130
IL-17	IL17A	interleukin 17A	HGNC:5981	160
IL-6	IL6	interleukin 6	HGNC:6018	27,143
IL-6R	IL6R	interleukin 6 receptor	HGNC:6019	
IL-8	CXCL8	C-X-C motif chemokine ligand 8	HGNC:6025	32
ILK	ILK	integrin linked kinase	HGNC:6040	113,132
RICTOR	RICTOR	RPTOR independent companion of MTOR complex 2	HGNC:28611	132
Jagged	JAG1	jagged canonical Notch ligand 1	HGNC:6188	33,34,161
	JAG2	jagged canonical Notch ligand 2	HGNC:6189	
KLF-8	KLF8	Kruppel like factor 8	HGNC:6351	15
L-TGFB1	LTBP1	latent transforming growth factor beta binding protein 1	HGNC:6714	12,20,105,108
LBX1	LBX1	ladybird homeobox 1	HGNC:16960	64
Linck				162
Lipocalin 2	LNC2	lipocalin 2	HGNC:6526	13,163

MAPK			HNGC Group ID 651	22
p130CAS	BCAR1	BCAR1 scaffold protein, Cas family member	HGNC:971	164
p21	CDKN1A	cyclin dependent kinase inhibitor 1A	HGNC:1784	107,138,139
p38	MAPK14	mitogen-activated protein kinase 14	HGNC:6876	22
p53	TP53	tumor protein p53	HGNC:11998	6-8,56,165
p63	TP63	tumor protein p63	HGNC:15979	65
src	SRC	SRC proto-oncogene, non-receptor tyrosine kinase	HGNC:11283	57,104,117,135,166
uPA	PLAU	plasminogen activator, urokinase	HGNC:9052	20,143
MTA-1	MTA1	metastasis associated 1	HGNC:7410	154
SUZ12	SUZ12	SUZ12 polycomb repressive complex 2 subunit	HGNC:17101	146
CK1a	CSNK1A1	casein kinase 1 alpha 1	HGNC:2451	167
APC	APC	APC regulator of WNT signaling pathway	HGNC:583	75,77,158
CHMP3	CHMP3	charged multivesicular body protein 3	HGNC:29865	168
FZD2	FZD2	frizzled class receptor 2	HGNC:4040	35
HOTAIR	HOTAIR	HOX transcript antisense RNA	HGNC:33510	16,71,169,170
AC026904.1	LINC02599	long intergenic non-protein coding RNA 2599	HGNC:53931	109
ATB	LNCRNA-ATB	lncRNA activated by TGF-beta	HGNC:52657	171,172
LINP-1	LINP1	lncRNA in non-homologous end joining pathway 1	HGNC:53170	173,174
MALAT	MALAT1	metastasis associated lung adenocarcinoma transcript 1	HGNC:29665	43,116
MEG3	MEG3	maternally expressed 3	HGNC:14575	7,8,175
NKILA	NKILA	NF-kappaB interacting lncRNA	HGNC:51599	30

ROR	LINC-ROR	long intergenic non-protein coding RNA, regulator of reprogramming	HGNC:43773	131
XIST	XIST	X inactive specific transcript	HGNC:12810	176
ANCR	DANCR	differentiation antagonizing non-protein coding RNA	HGNC:28964	63
miR-1	MIR1-1	microRNA 1-1	HGNC:31499	43
miR-106b-25	MIR106B	microRNA 106b	HGNC:31495	74,137
miR-10b	MIR10B	microRNA 10b	HGNC:31498	60,177,178
miR-122-5p	MIR122	microRNA 122	HGNC:31501	168
miR-124	MIR124-1	microRNA 124-1	HGNC:31502	179,180
	MIR124-2	microRNA 124-2	HGNC:31503	
	MIR124-3	microRNA 124-3	HGNC:31504	
miR-132	MIR132	microRNA 132	HGNC:31516	158
miR-133b	MIR133B	microRNA 133b	HGNC:31759	181
miR-141-3p	MIR141	microRNA 141	HGNC:31528	172,182
miR-145	MIR145	microRNA 145	HGNC:31532	24,131,183
miR-148	MIR148A	microRNA 148a	HGNC:31535	184
miR-153	MIR153-1	microRNA 153-1	HGNC:31539	185
miR-155	MIR155	microRNA 155	HGNC:31542	67,121
miR-181a	MIR181A2	microRNA 181a-2	HGNC:31549	136,186
miR-182	MIR182	microRNA 182	HGNC:31553	11,82
miR-190	MIR190A	microRNA 190a	HGNC:31560	44
miR-200	MIR200A	microRNA 200a	HGNC:31578	45,115,187
	MIR200B	microRNA 200b	HGNC:31579	
	MIR200C	microRNA 200c	HGNC:31580	

miR-203	MIR203A	microRNA 203a	HGNC:31581	85,188
miR-204	MIR204	microRNA 204	HGNC:31582	116
miR-20a-5p	MIR20A	microRNA 20a	HGNC:31577	16,170
miR-21	MIR21	microRNA 21	HGNC:31586	54,55,189
miR-218-5p	MIR218-1	microRNA 218-1	HGNC:31595	146
	MIR218-2	microRNA 218-2	HGNC:31596	
miR-23a	MIR23A	microRNA 23a	HGNC:31605	119
miR-23b	MIR23B	microRNA 23b	HGNC:31606	41
miR-29	MIR29A	microRNA 29a	HGNC:31616	173,174
	MIR29C	microRNA 29c	HGNC:31621	
miR-34	MIR34A	microRNA 34a	HGNC:31635	16,190,191
	MIR34B	microRNA 34b	HGNC:31636	
	MIR34C	microRNA 34c	HGNC:31637	
miR-373	MIR373	microRNA 373	HGNC:31787	102,192
miR-381-3p	MIR381	microRNA 381	HGNC:31874	91
miR-421	MIR421	microRNA 421	HGNC:32793	175
miR-455-3p	MIR455	microRNA 455	HGNC:32344	154
miR-520c	MIR520C	microRNA 520c	HGNC:32108	102,159,192
miR-584	MIR584	microRNA 584	HGNC:32840	46
miR-615-3p	MIR615	microRNA 615	HGNC:32871	51
miR-630	MIR630	microRNA 630	HGNC:32886	25,193
miR-644	MIR644A	microRNA 644a	HGNC:32900	114
miR-7	MIR7-1	microRNA 7-1	HGNC:31638	42,71
	MIR7-2	microRNA 7-2	HGNC:31639	
	MIR7-3	microRNA 7-3	HGNC:31640	

miR-9	MIR9-1	microRNA 9-1	HGNC:31641	194
	MIR9-2	microRNA 9-2	HGNC:31642	
	MIR9-3	microRNA 9-3	HGNC:31646	

Supplementary Table 2. Hub regulators contributing to the invasiveness

Regulator	References
P21	107,138,139
CD44	141-143
E-Cadherin	73,147,148
β-Catenin	12,36,118,119
P38	4,22,125,135,195
Cyclin D1	95,118,125,126,145
NF-kB	13,14,19,27-32
RAS	19,64-66
ERK	4,28,29
Annexin 2	9,57,95,134
PAK2	41-43
EGFR	94,134
MMPs	4,5,12-22
miR-7	42,71
L-TGFB1	12,20,105,108
TWIST	36,106-108
miR-200	45,115,187
P53	6-8,56,165
ZEB	64,115-117
SMAD2_SMAD4	78-80
SMAD3_SMAD4	78-80

SNAIL	1,23,30,31,45,53,59,85-90
STAT3	95-97
c-MYC	53,107,138-140
TGFβ	70,98 100 29,102-104

Supplementary Table 3: List of cell lines used in experiments from which the regulators and the regulatory mechanisms are curated

Cell Line	Origin (Human or Other animal)	Tissue	Cell Type	Tumor Status	Triple Negative Breast Cancer Status (ER, PR, HER)	Gene Expression Data (CCLE)	Resource
MCF-10A	Human	Breast, Mammary Gland	Epithelial	Primary	TNBC -	yes	ATCC
BT474	Human	Breast, Duct,Mammary Gland	Epithelial	Primary	TNBC(ER,PR,HER)	yes	ATCC
MDA-MB-231	Human	Breast, Mammary Gland	Epithelial	Metastasis	TNBC -	yes	ATCC
Hs578T	Human	Breast, Mammary Gland	Epithelial	Primary	TNBC -	yes	ATCC
T47D	Human	Breast, Mammary Gland	Epithelial	Metastasis	TNBC(ER)	yes	ATCC
MCF7	Human	Breast, Mammary Gland	Epithelial	Metastasis	TNBC(ER)	yes	ATCC
MDA-MB-468	Human	Breast, Mammary Gland	Epithelial	Metastasis	TNBC -	yes	ATCC
4T1	mouse	Breast, Mammary Gland	Epithelial	Metastasis	TNBC -	no	ATCC
ZR75B	Human	Breast, Mammary Gland	Epithelial	Metastasis	TNBC(ER)	yes	OLS
BT549	Human	Breast, Mammary Gland	Epithelial	Primary	TNBC -	yes	ATCC
MDA-MB-436	Human	Breast, Mammary Gland	Epithelial	Metastasis	TNBC -	yes	ATCC
SKBR3	Human	Breast, Mammary Gland	Epithelial	Metastasis	TNBC(HER)	yes	ATCC
NMuMG	Mus musculus, mouse	Breast, Mammary Gland	Epithelial	Metastasis		no	ATCC
BT-20	Human	Breast, Mammary Gland	Epithelial	Primary	TNBC -	yes	ATCC
MDA-MB-453	Human	Breast, Mammary Gland	Epithelial	Metastasis	TNBC(HER)	yes	ATCC
MDA-MB-157	Human	Breast; Mammary gland; Medulla	Epithelial	Metastasis	TNBC -	yes	ATCC
HCC70	Human	Breast; Duct; Mammary gland	Epithelial	Primary	TNBC -	yes	ATCC
HCC1937	Human	Breast; Duct; Mammary gland	lymphoblast	Primary	TNBC -	yes	ATCC

MDA-MB-361	Human	Breast, Mammary Gland	Epithelial	Metastasis	TNBC(ER,partial PR,HER)	yes	ATCC
MCF-12A	Human	Breast, Mammary Gland	Epithelial			yes	ATCC
ZR-75-1	Human	Breast; Duct; Mammary gland	Epithelial	Metastasis	TNBC(ER,partial PR)	yes	ATCC
BT-483	Human	Breast, Mammary Gland	Epithelial	Primary	TNBC(ER,partial PR)	yes	ATCC
CAMA-1	Human	Breast, Mammary Gland	Epithelial	Metastasis	TNBC(ER,partial PR)	yes	ATCC
AU565	Human	Breast, Mammary Gland	Epithelial	Metastasis	TNBC(HER)	yes	ATCC
SUM1315	Human	Breast	Epithelial	Metastasis	TNBC -	yes	Cellosaurus
HBL-100	Human	Breast	Epithelial			yes	Cellosaurus
67NR	Mus musculus (Mouse)	Mammary Gland	Epithelial	Primary	TNBC(ER)	no	Cellosaurus
168FARN	Mus musculus (Mouse)	Mammary Gland	Epithelial	Primary		no	Cellosaurus
4TO7	Mus musculus (Mouse)	Mammary Gland	Epithelial	Primary		no	Cellosaurus
EpH4	Mus musculus (Mouse)	Breast,Mammary Gland	Epithelial			no	Cellosaurus
MCF10CA1a	Human	Breast	Epithelial	Metastasis		no	Cellosaurus
MCF10CA1d	Human	Breast	Epithelial			no	Cellosaurus
MCF10CA1h	Human	Breast	Epithelial			no	Cellosaurus
SUM159	Human	Breast	Epithelial	Primary	TNBC-	yes	Cellosaurus
MCF7/LCC9	Human	Breast	Epithelial	Metastasis		no	Cellosaurus
MDA-MB-435	Human	Breast	hypodermis	Metastasis	TNBC(HER)	no	Cellosaurus
MDA-MB-231-lucD3H2LN	Human	Breast	Epithelial	Metastasis	TNBC-	no	Cellosaurus
SKBR3-LR	Human	Breast	Epithelial	Metastasis		no	Cellosaurus
HCC1954-LR	Human	Breast	Epithelial			no	Cellosaurus
MCF10A/HER2	Human	Breast	Epithelial		TNBC(HER)	no	Cellosaurus
MDA-MB-231 LM24175	Human	Breast	Epithelial	Metastasis	TNBC-	no	Cellosaurus
4T1-Luc	Mus musculus (Mouse)	Mammary gland	Epithelial			no	Cellosaurus

MDA-MB231-LucD3H1	Human	Breast	Epithelial	Metastasis	TNBC-	no	Cellosaurus
MCF10AT	Human	Breast	Epithelial			no	Cellosaurus
SCP2	Mouse	Mammary gland	Epithelial	Metastasis	TNBC-	no	ATCC
HMLE	Human	Mammary gland	Epithelial			no	lonza
ZR75	Human	Mammary Gland	Epithelial	Primary			ATCC
A549	Human	Lung	Epithelial	Primary		yes	ATCC
A375	Human	skin	Epithelial	Primary		yes	ATCC
Hela	Human	Uterus; Cervix	Epithelial	Metastasis		yes	ATCC
Panc-1	Human	Pancreas; Duct	Epithelial	Primary		yes	ATCC
Hs766T	Human	Pancreas	Epithelial	Metastasis		yes	ATCC
293T	Human	kidney; Embryo	Epithelial	Primary		no	ATCC
HCT116	Human	Large intestine; Colon	Epithelial	Primary		yes	ATCC
U2OS	Human	Bone	Epithelial	Primary		yes	ATCC
HPAECs	Human	Pulmonary artery	endothelial cell			no	ATCC
HLMVECs	Human	Lung	endothelial cell			no	ols
U937	Human	Lung	monocyte	Metastasis		yes	ATCC
HCT15	Human	Large intestine; Colon	Epithelial	Primary		yes	ATCC
HEK293	Human	kidney; Embryo	Epithelial	Metastasis		no	ATCC
WI-38	Human	Lung	fibroblast			no	ATCC
H1299	Human	Lung	Epithelial	Metastasis		yes	ATCC
LoVo	Human	Large intestine; Colon	Epithelial	Metastasis		yes	ATCC
T24	Human	Urinary bladder	Epithelial	Primary		yes	ATCC
MKN-45	Human	Liver	Epithelial	Metastasis		yes	Cellosaurus
AGS	Human	Stomach	Epithelial	Primary		yes	ATCC
SKOV3	Human	Ovary; Ascites	Epithelial	Metastasis		yes	ATCC

HepG2	Human	Liver	Epithelial	Primary		yes	ATCC
HacaT	Human	skin	keratinocyte	Primary		no	Cellosaurus
MDCK	Canis familiaris, dog	kidney	Epithelial			no	ATCC
HuH7	Human	Liver	Epithelial	Primary		yes	Cellosaurus
BoM-1833 h	Human	Bone	Epithelial	Metastasis		no	Cellosaurus
Dr26	Human	Lung	Epithelial			no	radioprotection
SV40 MES 13	Mus musculus (Mouse)	kidney; Glomerulus	mesangial cell			no	ATCC
MDA-MB-435S	Human	skin	melanocyte	Metastasis		yes	ATCC

Supplementary References:

1 Araki, S. *et al.* TGF-beta1-induced expression of human Mdm2 correlates with late-stage metastatic breast cancer. *J Clin Invest* **120**, 290-302 (2010). https://doi.org/10.1172/JCI39194

2 Chen, Y. *et al.* MDM2 promotes epithelial-mesenchymal transition and metastasis of ovarian cancer SKOV3 cells. *Br J Cancer* **117**, 1192-1201 (2017). https://doi.org/10.1038/bjc.2017.265

3 Lu, X. *et al.* Mouse double minute 2 (MDM2) upregulates Snail expression and induces epithelial-to-mesenchymal transition in breast cancer cells in vitro and in vivo. *Oncotarget* **7**, 37177-37191 (2016). https://doi.org/10.18632/oncotarget.9287

4 Shin, I., Kim, S., Song, H., Kim, H. R. & Moon, A. H-Ras-specific activation of Rac-MKK3/6-p38 pathway: its critical role in invasion and migration of breast epithelial cells. *J Biol Chem* **280**, 14675-14683 (2005). https://doi.org/10.1074/jbc.M411625200

5 Song, H., Ki, S. H., Kim, S. G. & Moon, A. Activating transcription factor 2 mediates matrix metalloproteinase-2 transcriptional activation induced by p38 in breast epithelial cells. *Cancer Res* **66**, 10487-10496 (2006). https://doi.org/10.1158/0008-5472.CAN-06-1461

6 Zhou, Y. *et al.* Activation of p53 by MEG3 non-coding RNA. *J Biol Chem* **282**, 24731-24742 (2007). https://doi.org/10.1074/jbc.M702029200

7 Hao, Y., Baker, D. & Ten Dijke, P. TGF-beta-Mediated Epithelial-Mesenchymal Transition and Cancer Metastasis. *Int J Mol Sci* **20**, 2767 (2019). https://doi.org/10.3390/ijms20112767

8 Mondal, T. *et al.* MEG3 long noncoding RNA regulates the TGF-β pathway genes through formation of RNA–DNA triplex structures. *Nature Communications* **6**, 7743 (2015). https://doi.org/10.1038/ncomms8743

9 Kpetemey, M. *et al.* MIEN1, a novel interactor of Annexin A2, promotes tumor cell migration by enhancing AnxA2 cell surface expression. *Mol Cancer* **14**, 156 (2015). https://doi.org/10.1186/s12943-015-0428-8

10 Izumchenko, E. *et al.* The TGFbeta-miR200-MIG6 pathway orchestrates the EMT-associated kinase switch that induces resistance to EGFR inhibitors. *Cancer Res* **74**, 3995-4005 (2014). https://doi.org/10.1158/0008-5472.CAN-14-0110

11 Lei, R. *et al.* Suppression of MIM by microRNA-182 activates RhoA and promotes breast cancer metastasis. *Oncogene* **33**, 1287-1296 (2014). https://doi.org/10.1038/onc.2013.65

12 Walsh, L. A. & Damjanovski, S. IGF-1 increases invasive potential of MCF 7 breast cancer cells and induces activation of latent TGF-beta1 resulting in epithelial to mesenchymal transition. *Cell Commun Signal* **9**, 10 (2011). https://doi.org/10.1186/1478-811X-9-10

13 Leng, X. *et al.* Inhibition of lipocalin 2 impairs breast tumorigenesis and metastasis. *Cancer Res* **69**, 8579-8584 (2009). https://doi.org/10.1158/0008-5472.CAN-09-1934

14 Huber, M. A. *et al.* NF-kappaB is essential for epithelial-mesenchymal transition and metastasis in a model of breast cancer progression. *J Clin Invest* **114**, 569-581 (2004). https://doi.org/10.1172/JCI21358

15 Wang, X. *et al.* KLF8 promotes human breast cancer cell invasion and metastasis by transcriptional activation of MMP9. *Oncogene* **30**, 1901-1911 (2011). https://doi.org/10.1038/onc.2010.563
16 Mozdarani, H., Ezzatizadeh, V. & Rahbar Parvaneh, R. The emerging role of the long non-coding RNA HOTAIR in breast cancer development and treatment. *J Transl Med* **18**, 152 (2020). https://doi.org/10.1186/s12967-020-02320-0
17 Zhao, L., Pang, A. & Li, Y. Function of GCN5 in the TGF-beta1-induced epithelial-to-mesenchymal transition in breast cancer. *Oncol Lett* **16**, 3955-3963 (2018). https://doi.org/10.3892/ol.2018.9134
18 Fu, J. *et al.* The natural compound codonolactone attenuates TGF-beta1-mediated epithelial-to-mesenchymal transition and motility of breast cancer cells. *Oncol Rep* **35**, 117-126 (2016). https://doi.org/10.3892/or.2015.4394
19 Mali, A. V., Joshi, A. A., Hegde, M. V. & Kadam, S. S. Enterolactone modulates the ERK/NF-kappaB/Snail signaling pathway in triple-negative breast cancer cell line MDA-MB-231 to revert the TGF-beta-induced epithelial-mesenchymal transition. *Cancer Biol Med* **15**, 137-156 (2018). https://doi.org/10.20892/j.issn.2095-3941.2018.0012
20 Santibanez, J. F., Obradovic, H., Kukolj, T. & Krstic, J. Transforming growth factor-beta, matrix metalloproteinases, and urokinase-type plasminogen activator interaction in the cancer epithelial to mesenchymal transition. *Dev Dyn* **247**, 382-395 (2018). https://doi.org/10.1002/dvdy.24554
21 Simic, P. *et al.* SIRT1 suppresses the epithelial-to-mesenchymal transition in cancer metastasis and organ fibrosis. *Cell Rep* **3**, 1175-1186 (2013). https://doi.org/10.1016/j.celrep.2013.03.019
22 Kim, E.-S., Kim, M.-S. & Moon, A. TGF-β-induced upregulation of MMP-2 and MMP-9 depends on p38 MAPK, but not ERK signaling in MCF10A human breast epithelial cells. *International journal of oncology* **25**, 1375-1382 (2004).
23 Fujita, N. *et al.* MTA3, a Mi-2/NuRD complex subunit, regulates an invasive growth pathway in breast cancer. *Cell* **113**, 207-219 (2003). https://doi.org/10.1016/s0092-8674(03)00234-4
24 Ma, J. *et al.* LincRNA-RoR/miR-145 promote invasion and metastasis in triple-negative breast cancer via targeting MUC1. *Biochem Biophys Res Commun* **500**, 614-620 (2018). https://doi.org/10.1016/j.bbrc.2018.04.119
25 Zhou, C. X. *et al.* MiR-630 suppresses breast cancer progression by targeting metadherin. *Oncotarget* **7**, 1288-1299 (2016). https://doi.org/10.18632/oncotarget.6339
26 Wu, K. *et al.* DACH1 Inhibits Transforming Growth Factor-β Signaling through Binding Smad4. *Journal of Biological Chemistry* **278**, 51673-51684 (2003). https://doi.org/10.1074/jbc.m310021200
27 Burnett, J. P. *et al.* Trastuzumab resistance induces EMT to transform HER2(+) PTEN(-) to a triple negative breast cancer that requires unique treatment options. *Sci Rep* **5**, 15821 (2015). https://doi.org/10.1038/srep15821
28 Lee, J. 3,3'-Diindolylmethane Inhibits TNF-alpha- and TGF-beta-Induced Epithelial-Mesenchymal Transition in Breast Cancer Cells. *Nutr Cancer* **71**, 992-1006 (2019). https://doi.org/10.1080/01635581.2019.1577979
29 Liao, S. J. *et al.* TGF-beta1 and TNF-alpha synergistically induce epithelial to mesenchymal transition of breast cancer cells by enhancing TAK1 activation. *J Cell Commun Signal* **13**, 369-380 (2019). https://doi.org/10.1007/s12079-019-00508-8

30 Wu, W. *et al.* LncRNA NKILA suppresses TGF-beta-induced epithelial-mesenchymal transition by blocking NF-kappaB signaling in breast cancer. *Int J Cancer* **143**, 2213-2224 (2018). https://doi.org/10.1002/ijc.31605
31 Lee, Y. J., Park, J. H. & Oh, S. M. Activation of NF-kappaB by TOPK upregulates Snail/Slug expression in TGF-beta1 signaling to induce epithelial-mesenchymal transition and invasion of breast cancer cells. *Biochem Biophys Res Commun* **530**, 122-129 (2020). https://doi.org/10.1016/j.bbrc.2020.07.015
32 Li, S. *et al.* TWIST1 associates with NF-kappaB subunit RELA via carboxyl-terminal WR domain to promote cell autonomous invasion through IL8 production. *BMC Biol* **10**, 73 (2012). https://doi.org/10.1186/1741-7007-10-73
33 Han, L. *et al.* The Notch pathway inhibits TGFbeta signaling in breast cancer through HEYL-mediated crosstalk. *Cancer Res* **74**, 6509-6518 (2014). https://doi.org/10.1158/0008-5472.CAN-14-0816
34 Zavadil, J., Cermak, L., Soto-Nieves, N. & Bottinger, E. P. Integration of TGF-beta/Smad and Jagged1/Notch signalling in epithelial-to-mesenchymal transition. *EMBO J* **23**, 1155-1165 (2004). https://doi.org/10.1038/sj.emboj.7600069
35 Tuluhong, D. *et al.* FZD2 promotes TGF-beta-induced epithelial-to-mesenchymal transition in breast cancer via activating notch signaling pathway. *Cancer Cell Int* **21**, 199 (2021). https://doi.org/10.1186/s12935-021-01866-3
36 Wu, Y. *et al.* A83-01 inhibits TGF-beta-induced upregulation of Wnt3 and epithelial to mesenchymal transition in HER2-overexpressing breast cancer cells. *Breast Cancer Res Treat* **163**, 449-460 (2017). https://doi.org/10.1007/s10549-017-4211-y
37 Tobar, N., Guerrero, J., Smith, P. C. & Martinez, J. NOX4-dependent ROS production by stromal mammary cells modulates epithelial MCF-7 cell migration. *Br J Cancer* **103**, 1040-1047 (2010). https://doi.org/10.1038/sj.bjc.6605847
38 Boudreau, H. E., Casterline, B. W., Rada, B., Korzeniowska, A. & Leto, T. L. Nox4 involvement in TGF-beta and SMAD3-driven induction of the epithelial-to-mesenchymal transition and migration of breast epithelial cells. *Free radical biology & medicine* **53**, 1489-1499 (2012). https://doi.org/10.1016/j.freeradbiomed.2012.06.016
39 Hedrick, E. & Safe, S. Transforming Growth Factor beta/NR4A1-Inducible Breast Cancer Cell Migration and Epithelial-to-Mesenchymal Transition Is p38alpha (Mitogen-Activated Protein Kinase 14) Dependent. *Mol Cell Biol* **37**, e00306-00317 (2017). https://doi.org/10.1128/MCB.00306-17
40 Wu, R. S. *et al.* OVOL2 antagonizes TGF-beta signaling to regulate epithelial to mesenchymal transition during mammary tumor metastasis. *Oncotarget* **8**, 39401-39416 (2017). https://doi.org/10.18632/oncotarget.17031
41 Pellegrino, L. *et al.* miR-23b regulates cytoskeletal remodeling, motility and metastasis by directly targeting multiple transcripts. *Nucleic Acids Res* **41**, 5400-5412 (2013). https://doi.org/10.1093/nar/gkt245
42 Cui, Y. X. *et al.* MicroRNA-7 suppresses the homing and migration potential of human endothelial cells to highly metastatic human breast cancer cells. *Br J Cancer* **117**, 89-101 (2017). https://doi.org/10.1038/bjc.2017.156
43 Chou, J. *et al.* MALAT1 induced migration and invasion of human breast cancer cells by competitively binding miR-1 with cdc42. *Biochem Biophys Res Commun* **472**, 262-269 (2016). https://doi.org/10.1016/j.bbrc.2016.02.102

44 Yu, Y. *et al.* miR-190 suppresses breast cancer metastasis by regulation of TGF-beta-induced epithelial-mesenchymal transition. *Mol Cancer* **17**, 70 (2018). https://doi.org/10.1186/s12943-018-0818-9
45 Noman, M. Z. *et al.* The immune checkpoint ligand PD-L1 is upregulated in EMT-activated human breast cancer cells by a mechanism involving ZEB-1 and miR-200. *Oncoimmunology* **6**, e1263412 (2017). https://doi.org/10.1080/2162402X.2016.1263412
46 Fils-Aimé, N. *et al.* MicroRNA-584 and the Protein Phosphatase and Actin Regulator 1 (PHACTR1), a New Signaling Route through Which Transforming Growth Factor-β Mediates the Migration and Actin Dynamics of Breast Cancer Cells*. *Journal of Biological Chemistry* **288**, 11807-11823 (2013). https://doi.org/https://doi.org/10.1074/jbc.M112.430934
47 Basho, R. K. *et al.* Targeting the PI3K/AKT/mTOR Pathway for the Treatment of Mesenchymal Triple-Negative Breast Cancer: Evidence From a Phase 1 Trial of mTOR Inhibition in Combination With Liposomal Doxorubicin and Bevacizumab. *JAMA Oncol* **3**, 509-515 (2017). https://doi.org/10.1001/jamaoncol.2016.5281
48 Li, J. *et al.* Fisetin Inhibited Growth and Metastasis of Triple-Negative Breast Cancer by Reversing Epithelial-to-Mesenchymal Transition via PTEN/Akt/GSK3beta Signal Pathway. *Front Pharmacol* **9**, 772 (2018). https://doi.org/10.3389/fphar.2018.00772
49 Wang, H. *et al.* Anticancer Mechanisms of Salinomycin in Breast Cancer and Its Clinical Applications. *Front Oncol* **11**, 654428 (2021). https://doi.org/10.3389/fonc.2021.654428
50 Wang, S. E. *et al.* Transforming growth factor beta engages TACE and ErbB3 to activate phosphatidylinositol-3 kinase/Akt in ErbB2-overexpressing breast cancer and desensitizes cells to trastuzumab. *Mol Cell Biol* **28**, 5605-5620 (2008). https://doi.org/10.1128/MCB.00787-08
51 Lei, B. *et al.* miR-615-3p promotes the epithelial-mesenchymal transition and metastasis of breast cancer by targeting PICK1/TGFBRI axis. *J Exp Clin Cancer Res* **39**, 71 (2020). https://doi.org/10.1186/s13046-020-01571-5
52 Zhao, B. *et al.* PICK1 promotes caveolin-dependent degradation of TGF-beta type I receptor. *Cell Res* **22**, 1467-1478 (2012). https://doi.org/10.1038/cr.2012.92
53 Yeh, H. W. *et al.* PSPC1 mediates TGF-beta1 autocrine signalling and Smad2/3 target switching to promote EMT, stemness and metastasis. *Nat Cell Biol* **20**, 479-491 (2018). https://doi.org/10.1038/s41556-018-0062-y
54 Luo, M. *et al.* MiRNA-21 mediates the antiangiogenic activity of metformin through targeting PTEN and SMAD7 expression and PI3K/AKT pathway. *Sci Rep* **7**, 43427 (2017). https://doi.org/10.1038/srep43427
55 Yu, X. *et al.* Silencing of MicroRNA-21 confers the sensitivity to tamoxifen and fulvestrant by enhancing autophagic cell death through inhibition of the PI3K-AKT-mTOR pathway in breast cancer cells. *Biomedicine & Pharmacotherapy* **77**, 37-44 (2016). https://doi.org/10.1016/j.biopha.2015.11.005
56 Xu, J. *et al.* 14-3-3zeta turns TGF-beta's function from tumor suppressor to metastasis promoter in breast cancer by contextual changes of Smad partners from p53 to Gli2. *Cancer Cell* **27**, 177-192 (2015). https://doi.org/10.1016/j.ccell.2014.11.025
57 Fan, Y. *et al.* Rack1 mediates tyrosine phosphorylation of Anxa2 by Src and promotes invasion and metastasis in drug-resistant breast cancer cells. *Breast Cancer Res* **21**, 66 (2019). https://doi.org/10.1186/s13058-019-1147-7

58 Yang, Y. *et al.* Rack1 Mediates the Interaction of P-Glycoprotein with Anxa2 and Regulates Migration and Invasion of Multidrug-Resistant Breast Cancer Cells. *Int J Mol Sci* **17**, 1718 (2016). https://doi.org/10.3390/ijms17101718

59 Wu, J. *et al.* RBM38 is involved in TGF-beta-induced epithelial-to-mesenchymal transition by stabilising zonula occludens-1 mRNA in breast cancer. *Br J Cancer* **117**, 675-684 (2017). https://doi.org/10.1038/bjc.2017.204

60 Ma, L. *et al.* Therapeutic silencing of miR-10b inhibits metastasis in a mouse mammary tumor model. *Nat Biotechnol* **28**, 341-347 (2010). https://doi.org/10.1038/nbt.1618

61 Bhowmick, N. A. *et al.* Transforming growth factor-beta1 mediates epithelial to mesenchymal transdifferentiation through a RhoA-dependent mechanism. *Mol Biol Cell* **12**, 27-36 (2001). https://doi.org/10.1091/mbc.12.1.27

62 Zhu, X. *et al.* HMOX-1 inhibits TGF-beta-induced epithelial-mesenchymal transition in the MCF-7 breast cancer cell line. *Int J Mol Med* **40**, 411-417 (2017). https://doi.org/10.3892/ijmm.2017.3027

63 Li, Z. *et al.* LncRNA ANCR down-regulation promotes TGF-beta-induced EMT and metastasis in breast cancer. *Oncotarget* **8**, 67329-67343 (2017). https://doi.org/10.18632/oncotarget.18622

64 Yu, M. *et al.* A developmentally regulated inducer of EMT, LBX1, contributes to breast cancer progression. *Genes Dev* **23**, 1737-1742 (2009). https://doi.org/10.1101/gad.1809309

65 Adorno, M. *et al.* A Mutant-p53/Smad complex opposes p63 to empower TGFbeta-induced metastasis. *Cell* **137**, 87-98 (2009). https://doi.org/10.1016/j.cell.2009.01.039

66 Janda, E. *et al.* Ras and TGF[beta] cooperatively regulate epithelial cell plasticity and metastasis: dissection of Ras signaling pathways. *J Cell Biol* **156**, 299-313 (2002). https://doi.org/10.1083/jcb.200109037

67 Kong, W. *et al.* MicroRNA-155 is regulated by the transforming growth factor beta/Smad pathway and contributes to epithelial cell plasticity by targeting RhoA. *Mol Cell Biol* **28**, 6773-6784 (2008). https://doi.org/10.1128/MCB.00941-08

68 Wardhani, B. W. K., Louisa, M., Watanabe, Y., Setiabudy, R. & Kato, M. TGF-beta-Induced TMEPAI Promotes Epithelial-Mesenchymal Transition in Doxorubicin-Treated Triple-Negative Breast Cancer Cells via SMAD3 and PI3K/AKT Pathway Alteration. *Breast Cancer (Dove Med Press)* **13**, 529-538 (2021). https://doi.org/10.2147/BCTT.S325429

69 Watanabe, Y. *et al.* TMEPAI, a transmembrane TGF-beta-inducible protein, sequesters Smad proteins from active participation in TGF-beta signaling. *Mol Cell* **37**, 123-134 (2010). https://doi.org/10.1016/j.molcel.2009.10.028

70 Korkut, A. *et al.* A Pan-Cancer Analysis Reveals High-Frequency Genetic Alterations in Mediators of Signaling by the TGF-β Superfamily. *Cell Systems* **7**, 422-437.e427 (2018). https://doi.org/10.1016/j.cels.2018.08.010

71 Zhang, H. *et al.* MiR-7, inhibited indirectly by lincRNA HOTAIR, directly inhibits SETDB1 and reverses the EMT of breast cancer stem cells by downregulating the STAT3 pathway. *Stem Cells* **32**, 2858-2868 (2014). https://doi.org/10.1002/stem.1795

72 Yang, W. *et al.* SETDB1 induces epithelialmesenchymal transition in breast carcinoma by directly binding with Snail promoter. *Oncol Rep* **41**, 1284-1292 (2019). https://doi.org/10.3892/or.2018.6871

73 Liarte, S., Alonso-Romero, J. L. & Nicolas, F. J. SIRT1 and Estrogen Signaling Cooperation for Breast Cancer Onset and Progression. *Front Endocrinol (Lausanne)* **9**, 552 (2018). https://doi.org/10.3389/fendo.2018.00552

74 Smith, A. L. *et al.* The miR-106b-25 cluster targets Smad7, activates TGF-beta signaling, and induces EMT and tumor initiating cell characteristics downstream of Six1 in human breast cancer. *Oncogene* **31**, 5162-5171 (2012). https://doi.org/10.1038/onc.2012.11

75 Le Scolan, E. *et al.* Transforming growth factor-beta suppresses the ability of Ski to inhibit tumor metastasis by inducing its degradation. *Cancer Res* **68**, 3277-3285 (2008). https://doi.org/10.1158/0008-5472.CAN-07-6793

76 Tecalco-Cruz, A. C., Rios-Lopez, D. G., Vazquez-Victorio, G., Rosales-Alvarez, R. E. & Macias-Silva, M. Transcriptional cofactors Ski and SnoN are major regulators of the TGF-beta/Smad signaling pathway in health and disease. *Signal Transduct Target Ther* **3**, 15 (2018). https://doi.org/10.1038/s41392-018-0015-8

77 Luo, K. Ski and SnoN: negative regulators of TGF-beta signaling. *Curr Opin Genet Dev* **14**, 65-70 (2004). https://doi.org/10.1016/j.gde.2003.11.003

78 Heldin, C. H., Miyazono, K. & ten Dijke, P. TGF-beta signalling from cell membrane to nucleus through SMAD proteins. *Nature* **390**, 465-471 (1997). https://doi.org/10.1038/37284

79 Attisano, L. & Wrana, J. L. Mads and Smads in TGF beta signalling. *Curr Opin Cell Biol* **10**, 188-194 (1998). https://doi.org/10.1016/s0955-0674(98)80141-5

80 Johnsen, S. A., Subramaniam, M., Janknecht, R. & Spelsberg, T. C. TGFbeta inducible early gene enhances TGFbeta/Smad-dependent transcriptional responses. *Oncogene* **21**, 5783-5790 (2002). https://doi.org/10.1038/sj.onc.1205681

81 Zhou, T. *et al.* GDF10 inhibits proliferation and epithelial-mesenchymal transition in triple-negative breast cancer via upregulation of Smad7. *Aging (Albany NY)* **11**, 3298-3314 (2019). https://doi.org/10.18632/aging.101983

82 Yu, J. *et al.* MicroRNA-182 targets SMAD7 to potentiate TGFbeta-induced epithelial-mesenchymal transition and metastasis of cancer cells. *Nat Commun* **7**, 13884 (2016). https://doi.org/10.1038/ncomms13884

83 Zhang, L. *et al.* USP4 is regulated by AKT phosphorylation and directly deubiquitylates TGF-beta type I receptor. *Nat Cell Biol* **14**, 717-726 (2012). https://doi.org/10.1038/ncb2522

84 Ito, I. *et al.* Estrogen inhibits transforming growth factor beta signaling by promoting Smad2/3 degradation. *J Biol Chem* **285**, 14747-14755 (2010). https://doi.org/10.1074/jbc.M109.093039

85 Ding, X., Park, S. I., McCauley, L. K. & Wang, C. Y. Signaling between transforming growth factor beta (TGF-beta) and transcription factor SNAI2 represses expression of microRNA miR-203 to promote epithelial-mesenchymal transition and tumor metastasis. *J Biol Chem* **288**, 10241-10253 (2013). https://doi.org/10.1074/jbc.M112.443655

86 Bai, J. W. *et al.* The zinc-finger transcriptional factor Slug transcriptionally downregulates ERalpha by recruiting lysine-specific demethylase 1 in human breast cancer. *Oncogenesis* **6**, e330 (2017). https://doi.org/10.1038/oncsis.2017.38

87 Dhasarathy, A., Phadke, D., Mav, D., Shah, R. R. & Wade, P. A. The transcription factors Snail and Slug activate the transforming growth factor-beta signaling pathway in breast cancer. *PLoS One* **6**, e26514 (2011). https://doi.org/10.1371/journal.pone.0026514

88 Ni, T. *et al.* Snail1-dependent p53 repression regulates expansion and activity of tumour-initiating cells in breast cancer. *Nat Cell Biol* **18**, 1221-1232 (2016). https://doi.org/10.1038/ncb3425
89 Li, H. M. *et al.* A potent CBP/p300-Snail interaction inhibitor suppresses tumor growth and metastasis in wild-type p53-expressing cancer. *Sci Adv* **6**, eaaw8500 (2020). https://doi.org/10.1126/sciadv.aaw8500
90 Thuault, S. *et al.* Transforming growth factor-beta employs HMGA2 to elicit epithelial-mesenchymal transition. *J Cell Biol* **174**, 175-183 (2006). https://doi.org/10.1083/jcb.200512110
91 Yu, Y. Z. *et al.* miR-381-3p suppresses breast cancer progression by inhibition of epithelial-mesenchymal transition. *World J Surg Oncol* **19**, 230 (2021). https://doi.org/10.1186/s12957-021-02344-w
92 Zhang, J. *et al.* SOX4 induces epithelial-mesenchymal transition and contributes to breast cancer progression. *Cancer Res* **72**, 4597-4608 (2012). https://doi.org/10.1158/0008-5472.CAN-12-1045
93 Tiwari, N. *et al.* Sox4 is a master regulator of epithelial-mesenchymal transition by controlling Ezh2 expression and epigenetic reprogramming. *Cancer Cell* **23**, 768-783 (2013). https://doi.org/10.1016/j.ccr.2013.04.020
94 Zhao, Y. *et al.* TGF-β transactivates EGFR and facilitates breast cancer migration and invasion through canonical Smad3 and ERK/Sp1 signaling pathways. *Molecular Oncology* **12**, 305-321 (2018). https://doi.org/10.1002/1878-0261.12162
95 Yuan, J. *et al.* Tyr23 phosphorylation of Anxa2 enhances STAT3 activation and promotes proliferation and invasion of breast cancer cells. *Breast Cancer Res Treat* **164**, 327-340 (2017). https://doi.org/10.1007/s10549-017-4271-z
96 Saitoh, M. *et al.* STAT3 integrates cooperative Ras and TGF-β signals that induce Snail expression. *Oncogene* **35**, 1049-1057 (2016). https://doi.org/10.1038/onc.2015.161
97 Gyamfi, J., Lee, Y. H., Eom, M. & Choi, J. Interleukin-6/STAT3 signalling regulates adipocyte induced epithelial-mesenchymal transition in breast cancer cells. *Sci Rep* **8**, 8859 (2018). https://doi.org/10.1038/s41598-018-27184-9
98 Wrana, J. L., Attisano, L., Wieser, R., Ventura, F. & Massague, J. Mechanism of activation of the TGF-beta receptor. *Nature* **370**, 341-347 (1994). https://doi.org/10.1038/370341a0
99 Ozdamar, B. *et al.* Regulation of the polarity protein Par6 by TGFbeta receptors controls epithelial cell plasticity. *Science* **307**, 1603-1609 (2005). https://doi.org/10.1126/science.1105718
100 Miettinen, P. J., Ebner, R., Lopez, A. R. & Derynck, R. TGF-beta induced transdifferentiation of mammary epithelial cells to mesenchymal cells: involvement of type I receptors. *J Cell Biol* **127**, 2021-2036 (1994). https://doi.org/10.1083/jcb.127.6.2021
101 Bandyopadhyay, A. *et al.* Inhibition of pulmonary and skeletal metastasis by a transforming growth factor-beta type I receptor kinase inhibitor. *Cancer Res* **66**, 6714-6721 (2006). https://doi.org/10.1158/0008-5472.CAN-05-3565
102 Keklikoglou, I. *et al.* MicroRNA-520/373 family functions as a tumor suppressor in estrogen receptor negative breast cancer by targeting NF-kappaB and TGF-beta signaling pathways. *Oncogene* **31**, 4150-4163 (2012). https://doi.org/10.1038/onc.2011.571

103 Tu, C. F., Wu, M. Y., Lin, Y. C., Kannagi, R. & Yang, R. B. FUT8 promotes breast cancer cell invasiveness by remodeling TGF-beta receptor core fucosylation. *Breast Cancer Res* **19**, 111 (2017). https://doi.org/10.1186/s13058-017-0904-8
104 Galliher, A. J. & Schiemann, W. P. Beta3 integrin and Src facilitate transforming growth factor-beta mediated induction of epithelial-mesenchymal transition in mammary epithelial cells. *Breast Cancer Res* **8**, R42 (2006). https://doi.org/10.1186/bcr1524
105 Lei, X., Bandyopadhyay, A., Le, T. & Sun, L. Autocrine TGFbeta supports growth and survival of human breast cancer MDA-MB-231 cells. *Oncogene* **21**, 7514-7523 (2002). https://doi.org/10.1038/sj.onc.1205966
106 Li, C. W. *et al.* Epithelial-mesenchymal transition induced by TNF-alpha requires NF-kappaB-mediated transcriptional upregulation of Twist1. *Cancer Res* **72**, 1290-1300 (2012). https://doi.org/10.1158/0008-5472.CAN-11-3123
107 Liu, M. *et al.* p21CIP1 attenuates Ras- and c-Myc-dependent breast tumor epithelial mesenchymal transition and cancer stem cell-like gene expression in vivo. *Proc Natl Acad Sci U S A* **106**, 19035-19039 (2009). https://doi.org/10.1073/pnas.0910009106
108 Xue, G. *et al.* Akt/PKB-mediated phosphorylation of Twist1 promotes tumor metastasis via mediating cross-talk between PI3K/Akt and TGF-beta signaling axes. *Cancer Discov* **2**, 248-259 (2012). https://doi.org/10.1158/2159-8290.CD-11-0270
109 Li, G.-Y. *et al.* Long non-coding RNAs AC026904.1 and UCA1: a "one-two punch" for TGF-β-induced SNAI2 activation and epithelial-mesenchymal transition in breast cancer. *Theranostics* **8**, 2846-2861 (2018). https://doi.org/10.7150/thno.23463
110 Taylor, M. A. *et al.* Upregulated WAVE3 expression is essential for TGF-beta-mediated EMT and metastasis of triple-negative breast cancer cells. *Breast Cancer Res Treat* **142**, 341-353 (2013). https://doi.org/10.1007/s10549-013-2753-1
111 Bledzka, K. *et al.* The WAVE3-YB1 interaction regulates cancer stem cells activity in breast cancer. *Oncotarget* **8**, 104072-104089 (2017). https://doi.org/10.18632/oncotarget.22009
112 Feldker, N. *et al.* Genome-wide cooperation of EMT transcription factor ZEB1 with YAP and AP-1 in breast cancer. *EMBO J* **39**, e103209 (2020). https://doi.org/10.15252/embj.2019103209
113 Gupta, P. & Srivastava, S. K. HER2 mediated de novo production of TGFbeta leads to SNAIL driven epithelial-to-mesenchymal transition and metastasis of breast cancer. *Mol Oncol* **8**, 1532-1547 (2014). https://doi.org/10.1016/j.molonc.2014.06.006
114 Raza, U. *et al.* The miR-644a/CTBP1/p53 axis suppresses drug resistance by simultaneous inhibition of cell survival and epithelial-mesenchymal transition in breast cancer. *Oncotarget* **7**, 49859-49877 (2016). https://doi.org/10.18632/oncotarget.10489
115 Bracken, C. P. *et al.* A double-negative feedback loop between ZEB1-SIP1 and the microRNA-200 family regulates epithelial-mesenchymal transition. *Cancer Res* **68**, 7846-7854 (2008). https://doi.org/10.1158/0008-5472.CAN-08-1942
116 Wang, Y. *et al.* MiR-204/ZEB2 axis functions as key mediator for MALAT1-induced epithelial-mesenchymal transition in breast cancer. *Tumour Biol* **39**, 1010428317690998 (2017). https://doi.org/10.1177/1010428317690998
117 Agajanian, M., Runa, F. & Kelber, J. A. Identification of a PEAK1/ZEB1 signaling axis during TGFbeta/fibronectin-induced EMT in breast cancer. *Biochem Biophys Res Commun* **465**, 606-612 (2015). https://doi.org/10.1016/j.bbrc.2015.08.071

118 Lin, S. Y. *et al.* Beta-catenin, a novel prognostic marker for breast cancer: its roles in cyclin D1 expression and cancer progression. *Proc Natl Acad Sci U S A* **97**, 4262-4266 (2000). https://doi.org/10.1073/pnas.060025397
119 Ma, F. *et al.* MiR-23a promotes TGF-beta1-induced EMT and tumor metastasis in breast cancer cells by directly targeting CDH1 and activating Wnt/beta-catenin signaling. *Oncotarget* **8**, 69538-69550 (2017). https://doi.org/10.18632/oncotarget.18422
120 Chen, L. *et al.* cPLA2alpha mediates TGF-beta-induced epithelial-mesenchymal transition in breast cancer through PI3k/Akt signaling. *Cell Death Dis* **8**, e2728 (2017). https://doi.org/10.1038/cddis.2017.152
121 Johansson, J. *et al.* MiR-155-mediated loss of C/EBPbeta shifts the TGF-beta response from growth inhibition to epithelial-mesenchymal transition, invasion and metastasis in breast cancer. *Oncogene* **32**, 5614-5624 (2013). https://doi.org/10.1038/onc.2013.322
122 Cieply, B. *et al.* Suppression of the Epithelial–Mesenchymal Transition by Grainyhead-like-2. *Cancer Research* **72**, 2440-2453 (2012). https://doi.org/10.1158/0008-5472.can-11-4038
123 Cieply, B., Farris, J., Denvir, J., Ford, H. L. & Frisch, S. M. Epithelial–Mesenchymal Transition and Tumor Suppression Are Controlled by a Reciprocal Feedback Loop between ZEB1 and Grainyhead-like-2. *Cancer Research* **73**, 6299-6309 (2013). https://doi.org/10.1158/0008-5472.can-12-4082
124 Xiang, X. *et al.* Grhl2 Determines the Epithelial Phenotype of Breast Cancers and Promotes Tumor Progression. *PLoS ONE* **7**, e50781 (2012). https://doi.org/10.1371/journal.pone.0050781
125 Li, T. *et al.* Daintain/AIF-1 promotes breast cancer cell migration by up-regulated TNF-alpha via activate p38 MAPK signaling pathway. *Breast Cancer Res Treat* **131**, 891-898 (2012). https://doi.org/10.1007/s10549-011-1519-x
126 Liu, S. *et al.* Daintain/AIF-1 promotes breast cancer proliferation via activation of the NF-kappaB/cyclin D1 pathway and facilitates tumor growth. *Cancer Sci* **99**, 952-957 (2008). https://doi.org/10.1111/j.1349-7006.2008.00787.x
127 Padua, D. *et al.* TGFbeta primes breast tumors for lung metastasis seeding through angiopoietin-like 4. *Cell* **133**, 66-77 (2008). https://doi.org/10.1016/j.cell.2008.01.046
128 Kang, Y. *et al.* A multigenic program mediating breast cancer metastasis to bone. *Cancer Cell* **3**, 537-549 (2003). https://doi.org/10.1016/s1535-6108(03)00132-6
129 Kang, Y. *et al.* Breast cancer bone metastasis mediated by the Smad tumor suppressor pathway. *Proc Natl Acad Sci U S A* **102**, 13909-13914 (2005). https://doi.org/10.1073/pnas.0506517102
130 Sundqvist, A. *et al.* Specific interactions between Smad proteins and AP-1 components determine TGFβ-induced breast cancer cell invasion. *Oncogene* **32**, 3606-3615 (2013). https://doi.org/10.1038/onc.2012.370
131 Eades, G. *et al.* lincRNA-RoR and miR-145 regulate invasion in triple-negative breast cancer via targeting ARF6. *Mol Cancer Res* **13**, 330-338 (2015). https://doi.org/10.1158/1541-7786.MCR-14-0251
132 Serrano, I., McDonald, P. C., Lock, F. E. & Dedhar, S. Role of the integrin-linked kinase (ILK)/Rictor complex in TGFβ-1-induced epithelial–mesenchymal transition (EMT). *Oncogene* **32**, 50-60 (2013). https://doi.org/10.1038/onc.2012.30

133 Cheng, G. Z. *et al.* Twist transcriptionally up-regulates AKT2 in breast cancer cells leading to increased migration, invasion, and resistance to paclitaxel. *Cancer Res* **67**, 1979-1987 (2007). https://doi.org/10.1158/0008-5472.CAN-06-1479

134 Wang, T. *et al.* Anxa2 binds to STAT3 and promotes epithelial to mesenchymal transition in breast cancer cells. *Oncotarget* **6**, 30975-30992 (2015). https://doi.org/10.18632/oncotarget.5199

135 Galliher, A. J. & Schiemann, W. P. Src phosphorylates Tyr284 in TGF-beta type II receptor and regulates TGF-beta stimulation of p38 MAPK during breast cancer cell proliferation and invasion. *Cancer Res* **67**, 3752-3758 (2007). https://doi.org/10.1158/0008-5472.CAN-06-3851

136 Taylor, M. A., Sossey-Alaoui, K., Thompson, C. L., Danielpour, D. & Schiemann, W. P. TGF-beta upregulates miR-181a expression to promote breast cancer metastasis. *J Clin Invest* **123**, 150-163 (2013). https://doi.org/10.1172/JCI64946

137 Petrocca, F. *et al.* E2F1-regulated microRNAs impair TGFbeta-dependent cell-cycle arrest and apoptosis in gastric cancer. *Cancer Cell* **13**, 272-286 (2008). https://doi.org/10.1016/j.ccr.2008.02.013

138 Massague, J., Blain, S. W. & Lo, R. S. TGFbeta signaling in growth control, cancer, and heritable disorders. *Cell* **103**, 295-309 (2000). https://doi.org/10.1016/s0092-8674(00)00121-5

139 Smith, A. P. *et al.* A positive role for Myc in TGFbeta-induced Snail transcription and epithelial-to-mesenchymal transition. *Oncogene* **28**, 422-430 (2009). https://doi.org/10.1038/onc.2008.395

140 Gomis, R. R., Alarcon, C., Nadal, C., Van Poznak, C. & Massague, J. C/EBPbeta at the core of the TGFbeta cytostatic response and its evasion in metastatic breast cancer cells. *Cancer Cell* **10**, 203-214 (2006). https://doi.org/10.1016/j.ccr.2006.07.019

141 Li, J. & Zhou, B. P. Activation of beta-catenin and Akt pathways by Twist are critical for the maintenance of EMT associated cancer stem cell-like characters. *BMC Cancer* **11**, 49 (2011). https://doi.org/10.1186/1471-2407-11-49

142 Vijay, G. V. *et al.* GSK3beta regulates epithelial-mesenchymal transition and cancer stem cell properties in triple-negative breast cancer. *Breast Cancer Res* **21**, 37 (2019). https://doi.org/10.1186/s13058-019-1125-0

143 Sheridan, C. *et al.* CD44+/CD24- breast cancer cells exhibit enhanced invasive properties: an early step necessary for metastasis. *Breast Cancer Res* **8**, R59 (2006). https://doi.org/10.1186/bcr1610

144 Yu, Y., Walia, V. & Elble, R. C. Loss of CLCA4 promotes epithelial-to-mesenchymal transition in breast cancer cells. *PLoS One* **8**, e83943 (2013). https://doi.org/10.1371/journal.pone.0083943

145 Zhang, F. *et al.* A novel Anxa2-interacting protein Ebp1 inhibits cancer proliferation and invasion by suppressing Anxa2 protein level. *Mol Cell Endocrinol* **411**, 75-85 (2015). https://doi.org/10.1016/j.mce.2015.04.013

146 Zhang, H. *et al.* A double-negative feedback loop between DEAD-box protein DDX21 and Snail regulates epithelial-mesenchymal transition and metastasis in breast cancer. *Cancer Letters* **437**, 67-78 (2018). https://doi.org/https://doi.org/10.1016/j.canlet.2018.08.021

147 Dong, C. *et al.* G9a interacts with Snail and is critical for Snail-mediated E-cadherin repression in human breast cancer. *J Clin Invest* **122**, 1469-1486 (2012). https://doi.org/10.1172/JCI57349
148 Zhang, H. *et al.* Forkhead transcription factor foxq1 promotes epithelial-mesenchymal transition and breast cancer metastasis. *Cancer Res* **71**, 1292-1301 (2011). https://doi.org/10.1158/0008-5472.CAN-10-2825
149 van Nimwegen, M. J., Verkoeijen, S., van Buren, L., Burg, D. & van de Water, B. Requirement for focal adhesion kinase in the early phase of mammary adenocarcinoma lung metastasis formation. *Cancer Res* **65**, 4698-4706 (2005). https://doi.org/10.1158/0008-5472.CAN-04-4126
150 Vander Ark, A., Cao, J. & Li, X. TGF-β receptors: In and beyond TGF-β signaling. *Cell Signal* **52**, 112-120 (2018). https://doi.org/10.1016/j.cellsig.2018.09.002
151 Huse, M., Chen, Y. G., Massague, J. & Kuriyan, J. Crystal structure of the cytoplasmic domain of the type I TGF beta receptor in complex with FKBP12. *Cell* **96**, 425-436 (1999). https://doi.org/10.1016/s0092-8674(00)80555-3
152 Huse, M. *et al.* The TGFβ Receptor Activation Process: An Inhibitor- to Substrate-Binding Switch. *Molecular Cell* **8**, 671-682 (2001). https://doi.org/https://doi.org/10.1016/S1097-2765(01)00332-X
153 Mani, S. A. *et al.* Mesenchyme Forkhead 1 (FOXC2) plays a key role in metastasis and is associated with aggressive basal-like breast cancers. *Proc Natl Acad Sci U S A* **104**, 10069-10074 (2007). https://doi.org/10.1073/pnas.0703900104
154 Zeng, Y. *et al.* MicroRNA-455-3p mediates GATA3 tumor suppression in mammary epithelial cells by inhibiting TGF-beta signaling. *J Biol Chem* **294**, 15808-15825 (2019). https://doi.org/10.1074/jbc.RA119.010800
155 Hartwell, K. A. *et al.* The Spemann organizer gene, Goosecoid, promotes tumor metastasis. *Proc Natl Acad Sci U S A* **103**, 18969-18974 (2006). https://doi.org/10.1073/pnas.0608636103
156 Peinado, H., Ballestar, E., Esteller, M. & Cano, A. Snail mediates E-cadherin repression by the recruitment of the Sin3A/histone deacetylase 1 (HDAC1)/HDAC2 complex. *Mol Cell Biol* **24**, 306-319 (2004). https://doi.org/10.1128/MCB.24.1.306-319.2004
157 Peng, J. *et al.* Hypoxia-Inducible Factor 1alpha Regulates the Transforming Growth Factor beta1/SMAD Family Member 3 Pathway to Promote Breast Cancer Progression. *J Breast Cancer* **21**, 259-266 (2018). https://doi.org/10.4048/jbc.2018.21.e42
158 Zhang, Z. G., Chen, W. X., Wu, Y. H., Liang, H. F. & Zhang, B. X. MiR-132 prohibits proliferation, invasion, migration, and metastasis in breast cancer by targeting HN1. *Biochem Biophys Res Commun* **454**, 109-114 (2014). https://doi.org/10.1016/j.bbrc.2014.10.049
159 Fang, Y. *et al.* Long non-coding RNA HOXA-AS2 promotes proliferation and invasion of breast cancer by acting as a miR-520c-3p sponge. *Oncotarget; Vol 8, No 28* (2017).
160 Nam, J.-S. *et al.* Transforming growth factor β subverts the immune system into directly promoting tumor growth through interleukin-17. *Cancer research* **68**, 3915-3923 (2008). https://doi.org/10.1158/0008-5472.CAN-08-0206
161 Boareto, M. *et al.* Notch-Jagged signalling can give rise to clusters of cells exhibiting a hybrid epithelial/mesenchymal phenotype. *Journal of the Royal Society Interface* **13**, 20151106 (2016). https://doi.org/10.1098/rsif.2015.1106

162 Li, J. *et al.* LincK contributes to breast tumorigenesis by promoting proliferation and epithelial-to-mesenchymal transition. *J Hematol Oncol* **12**, 19 (2019). https://doi.org/10.1186/s13045-019-0707-8
163 Yang, J. *et al.* Lipocalin 2 promotes breast cancer progression. *Proc Natl Acad Sci U S A* **106**, 3913-3918 (2009). https://doi.org/10.1073/pnas.0810617106
164 Wendt, M. K., Smith, J. A. & Schiemann, W. P. p130Cas is required for mammary tumor growth and transforming growth factor-beta-mediated metastasis through regulation of Smad2/3 activity. *J Biol Chem* **284**, 34145-34156 (2009). https://doi.org/10.1074/jbc.M109.023614
165 Parfenyev, S. *et al.* Interplay between p53 and non-coding RNAs in the regulation of EMT in breast cancer. *Cell Death Dis* **12**, 17 (2021). https://doi.org/10.1038/s41419-020-03327-7
166 Avizienyte, E. & Frame, M. C. Src and FAK signalling controls adhesion fate and the epithelial-to-mesenchymal transition. *Curr Opin Cell Biol* **17**, 542-547 (2005). https://doi.org/10.1016/j.ceb.2005.08.007
167 Guo, X. & Wang, X. F. Signaling cross-talk between TGF-beta/BMP and other pathways. *Cell Res* **19**, 71-88 (2009). https://doi.org/10.1038/cr.2008.302
168 Wang, Z. & Wang, X. miR-122-5p promotes aggression and epithelial-mesenchymal transition in triple-negative breast cancer by suppressing charged multivesicular body protein 3 through mitogen-activated protein kinase signaling. *Journal of Cellular Physiology* **235**, 2825-2835 (2020). https://doi.org/https://doi.org/10.1002/jcp.29188
169 Zhou, Y., Wang, C., Liu, X., Wu, C. & Yin, H. Long non-coding RNA HOTAIR enhances radioresistance in MDA-MB231 breast cancer cells. *Oncol Lett* **13**, 1143-1148 (2017). https://doi.org/10.3892/ol.2017.5587
170 Zhao, W., Geng, D., Li, S., Chen, Z. & Sun, M. LncRNA HOTAIR influences cell growth, migration, invasion, and apoptosis via the miR-20a-5p/HMGA2 axis in breast cancer. *Cancer Med* **7**, 842-855 (2018). https://doi.org/10.1002/cam4.1353
171 Li, R. H. *et al.* Long noncoding RNA ATB promotes the epithelial-mesenchymal transition by upregulating the miR-200c/Twist1 axe and predicts poor prognosis in breast cancer. *Cell Death Dis* **9**, 1171 (2018). https://doi.org/10.1038/s41419-018-1210-9
172 Zhang, Y. *et al.* Down-regulation of lncRNA-ATB inhibits epithelial-mesenchymal transition of breast cancer cells by increasing miR-141-3p expression. *Biochem Cell Biol* **97**, 193-200 (2019). https://doi.org/10.1139/bcb-2018-0168
173 Liang, Y. *et al.* Long noncoding RNA LINP1 acts as an oncogene and promotes chemoresistance in breast cancer. *Cancer Biol Ther* **19**, 120-131 (2018). https://doi.org/10.1080/15384047.2017.1394543
174 Zhang, Y. *et al.* Long noncoding RNA LINP1 regulates repair of DNA double-strand breaks in triple-negative breast cancer. *Nat Struct Mol Biol* **23**, 522-530 (2016). https://doi.org/10.1038/nsmb.3211
175 Zhang, W. *et al.* LncRNA MEG3 inhibits cell epithelial-mesenchymal transition by sponging miR-421 targeting E-cadherin in breast cancer. *Biomed Pharmacother* **91**, 312-319 (2017). https://doi.org/10.1016/j.biopha.2017.04.085
176 Zheng, R. *et al.* Long non-coding RNA XIST inhibited breast cancer cell growth, migration, and invasion via miR-155/CDX1 axis. *Biochem Biophys Res Commun* **498**, 1002-1008 (2018). https://doi.org/10.1016/j.bbrc.2018.03.104

177 Liu, Y. *et al.* MicroRNA-10b targets E-cadherin and modulates breast cancer metastasis. *Med Sci Monit* **18**, BR299-308 (2012). https://doi.org/10.12659/msm.883262
178 Ma, L., Teruya-Feldstein, J. & Weinberg, R. A. Tumour invasion and metastasis initiated by microRNA-10b in breast cancer. *Nature* **449**, 682-688 (2007). https://doi.org/10.1038/nature06174
179 Ji, H., Sang, M., Liu, F., Ai, N. & Geng, C. miR-124 regulates EMT based on ZEB2 target to inhibit invasion and metastasis in triple-negative breast cancer. *Pathol Res Pract* **215**, 697-704 (2019). https://doi.org/10.1016/j.prp.2018.12.039
180 Liang, Y. J. *et al.* MiR-124 targets Slug to regulate epithelial-mesenchymal transition and metastasis of breast cancer. *Carcinogenesis* **34**, 713-722 (2013). https://doi.org/10.1093/carcin/bgs383
181 Wang, S. *et al.* MicroRNA133b targets TGFbeta receptor I to inhibit TGFbetainduced epithelialtomesenchymal transition and metastasis by suppressing the TGFbeta/SMAD pathway in breast cancer. *Int J Oncol* **55**, 1097-1109 (2019). https://doi.org/10.3892/ijo.2019.4879
182 Neves, R. *et al.* Role of DNA methylation in miR-200c/141 cluster silencing in invasive breast cancer cells. *BMC Res Notes* **3**, 219 (2010). https://doi.org/10.1186/1756-0500-3-219
183 Ding, Y. *et al.* miR-145 inhibits proliferation and migration of breast cancer cells by directly or indirectly regulating TGF-beta1 expression. *Int J Oncol* **50**, 1701-1710 (2017). https://doi.org/10.3892/ijo.2017.3945
184 Xu, Q. *et al.* A regulatory circuit of miR-148a/152 and DNMT1 in modulating cell transformation and tumor angiogenesis through IGF-IR and IRS1. *J Mol Cell Biol* **5**, 3-13 (2013). https://doi.org/10.1093/jmcb/mjs049
185 Wang, J., Liang, S. & Duan, X. Molecular mechanism of miR-153 inhibiting migration, invasion and epithelial-mesenchymal transition of breast cancer by regulating transforming growth factor beta (TGF-beta) signaling pathway. *J Cell Biochem* **120**, 9539-9546 (2019). https://doi.org/10.1002/jcb.28230
186 Wang, Y. *et al.* Transforming growth factor-beta regulates the sphere-initiating stem cell-like feature in breast cancer through miRNA-181 and ATM. *Oncogene* **30**, 1470-1480 (2011). https://doi.org/10.1038/onc.2010.531
187 Gregory, P. A. *et al.* An autocrine TGF-beta/ZEB/miR-200 signaling network regulates establishment and maintenance of epithelial-mesenchymal transition. *Mol Biol Cell* **22**, 1686-1698 (2011). https://doi.org/10.1091/mbc.E11-02-0103
188 Wang, C., Zheng, X., Shen, C. & Shi, Y. MicroRNA-203 suppresses cell proliferation and migration by targeting BIRC5 and LASP1 in human triple-negative breast cancer cells. *J Exp Clin Cancer Res* **31**, 58 (2012). https://doi.org/10.1186/1756-9966-31-58
189 Yan, L. X. *et al.* MicroRNA miR-21 overexpression in human breast cancer is associated with advanced clinical stage, lymph node metastasis and patient poor prognosis. *RNA* **14**, 2348-2360 (2008). https://doi.org/10.1261/rna.1034808
190 Siemens, H. *et al.* miR-34 and SNAIL form a double-negative feedback loop to regulate epithelial-mesenchymal transitions. *Cell Cycle* **10**, 4256-4271 (2011). https://doi.org/10.4161/cc.10.24.18552
191 Imani, S. *et al.* MicroRNA-34a targets epithelial to mesenchymal transition-inducing transcription factors (EMT-TFs) and inhibits breast cancer cell migration and invasion. *Oncotarget* **8**, 21362-21379 (2017). https://doi.org/10.18632/oncotarget.15214

192 Huang, Q. *et al.* The microRNAs miR-373 and miR-520c promote tumour invasion and metastasis. *Nat Cell Biol* **10**, 202-210 (2008). https://doi.org/10.1038/ncb1681
193 Corcoran, C. *et al.* miR-630 targets IGF1R to regulate response to HER-targeting drugs and overall cancer cell progression in HER2 over-expressing breast cancer. *Mol Cancer* **13**, 71 (2014). https://doi.org/10.1186/1476-4598-13-71
194 Ma, L. *et al.* miR-9, a MYC/MYCN-activated microRNA, regulates E-cadherin and cancer metastasis. *Nat Cell Biol* **12**, 247-256 (2010). https://doi.org/10.1038/ncb2024
195 Yu, L., Hebert, M. C. & Zhang, Y. E. TGF-beta receptor-activated p38 MAP kinase mediates Smad-independent TGF-beta responses. *EMBO J* **21**, 3749-3759 (2002). https://doi.org/10.1093/emboj/cdf366
